# Biomarker analysis of the MITO2 phase III trial of first-line treatment in ovarian cancer: predictive value of DNA-PK and phosphorylated ACC

**DOI:** 10.18632/oncotarget.12056

**Published:** 2016-09-15

**Authors:** Francesco Perrone, Gustavo Baldassarre, Stefano Indraccolo, Simona Signoriello, Gennaro Chiappetta, Franca Esposito, Gabriella Ferrandina, Renato Franco, Delia Mezzanzanica, Maura Sonego, Elisabetta Zulato, Gian F. Zannoni, Vincenzo Canzonieri, Giovanni Scambia, Roberto Sorio, Antonella Savarese, Enrico Breda, Paolo Scollo, Antonella Ferro, Stefano Tamberi, Antonio Febbraro, Donato Natale, Massimo Di Maio, Daniela Califano, Giosuè Scognamiglio, Domenica Lorusso, Silvana Canevari, Simona Losito, Ciro Gallo, Sandro Pignata

**Affiliations:** ^1^ Istituto Nazionale per lo Studio e la Cura dei Tumori - Fondazione G. Pascale, IRCCS, Napoli, Italy; ^2^ Centro di Riferimento Oncologico, IRCCS, Aviano (PN), Italy; ^3^ Istituto Oncologico Veneto, IRCCS, Padova, Italy; ^4^ Dipartimento di Salute Mentale, Fisica e Medicina Preventiva, Statistica Medica, Seconda Università, Napoli, Italy; ^5^ Università di Napoli Federico II, Napoli, Italy; ^6^ Catholic University, Roma, Italy; ^7^ Fondazione IRCCS Istituto Nazionale dei Tumori, Milano, Italy; ^8^ Istituto Nazionale Tumori Regina Elena, IRCCS, Roma, Italy; ^9^ Ospedale S. Giovanni Calibita Fatebenefratelli, Roma, Italy; ^10^ Ospedale Cannizzaro, Catania, Italy; ^11^ Ospedale S. Chiara, Trento, Italy; ^12^ Ospedale Civile, Faenza, Italy; ^13^ Ospedale Fatebenefratelli, Benevento, Italy; ^14^ Ospedale S. Massimo, Penne (PE), Italy; ^15^ Dipartimento di Salute mentale, Fisica e Medicina Preventiva, Anatomia Patologica, Seconda Università, Napoli Italy; ^16^ Università degli Studi, Torino, Italy

**Keywords:** ovarian cancer, phase 3 clinical trial, predictive factors, pACC, DNA-PK

## Abstract

**Background:**

No biomarker is available to predict prognosis of patients with advanced ovarian cancer (AOC) and guide the choice of chemotherapy. We performed a prospective-retrospective biomarker study within the MITO2 trial on the treatment of AOC.

Patients and methods: MITO2 is a randomised multicentre phase 3 trial conducted with 820 AOC patients assigned carboplatin/paclitaxel (carboplatin: AUC5, paclitaxel: 175 mg/m², every 3 weeks for 6 cycles) or carboplatin/PLD-pegylated liposomal doxorubicin (carboplatin: AUC5, PLD: 30 mg/m², every 3 weeks for 6 cycles) as first line treatment. Sixteen biomarkers (pathways of adhesion/invasion, apoptosis, transcription regulation, metabolism, and DNA repair) were studied in 229 patients, in a tissue microarray. Progression-free and overall survival were analysed with multivariable Cox model.

**Results:**

After 72 months median follow-up, 594 progressions and 426 deaths were reported; there was no significant difference between the two arms in the whole trial. No biomarker had significant prognostic value. Statistically significant interactions with treatment were found for DNA-dependent protein kinase (DNA-PK) and phosphorylated acetyl-coenzymeA carboxylase (pACC), both predicting worse outcome for patients receiving carboplatin/paclitaxel.

**Conclusion:**

These data show that in presence of DNA-PK or pACC overexpression, carboplatin/paclitaxel might be less effective than carboplatin/PLD as first line treatment of ovarian cancer patients. Further validation of these findings is warranted.

## INTRODUCTION

Ovarian cancer includes a heterogeneous group of neoplasms, commonly classified according to clinical, morphological and molecular features. [1] Nevertheless, medical treatment of all types of epithelial ovarian cancer includes carboplatin/paclitaxel as standard backbone, also in combination with bevacizumab. [2-4] Therefore, biomarkers able to identify patients who may benefit from standard or alternative chemotherapy might be useful in clinical practice.

The possibility of grouping ovarian cancer based on molecular portrait has been proposed, [5, 6] and biochemical and molecular markers have been studied as prognostic or predictive factors. [7-9] However, only few immunohistochemistry (IHC) studies have been conducted in large series, through consortia or as meta-analysis. [8, 10, 11] Yet, with the exception of BRCA1/2 mutations to guide administration of PARP inhibitors, [12, 13] no biomarker is used in clinical practice.

In 2011, with a 40 months median follow-up, the MITO2 trial showed that carboplatin/PLD was not superior to carboplatin/paclitaxel as first line treatment of ovarian cancer patients. [14] Here, with long-term follow up data, we tested the prognostic and/or predictive role of some biomarkers according to a prospective-retrospective design. [15]

## RESULTS

### Long-term efficacy analysis in the whole study population

After 72 months median follow-up (interquartile range: 60-85), in the whole population of 820 patients, there were 594 events for PFS and 426 events for OS analysis. PFS HR for the carboplatin/PLD *vs* carboplatin/paclitaxel arm was 0.99 (95%CI 0.84-1.17; *p* = 0.93); median PFS was 18 (95%CI 16-23) and 17 months (95%CI 15-19) in the two arms, respectively. OS HR for the carboplatin/PLD *vs* carboplatin/paclitaxel arm was 0.94 (95%CI 0.77-1.13; *p* = 0.49); median OS was 61 (95%CI 51-77) and 53 months (95%CI 42-70) in the two arms, respectively (Figure S1 online).

**Figure 1:**
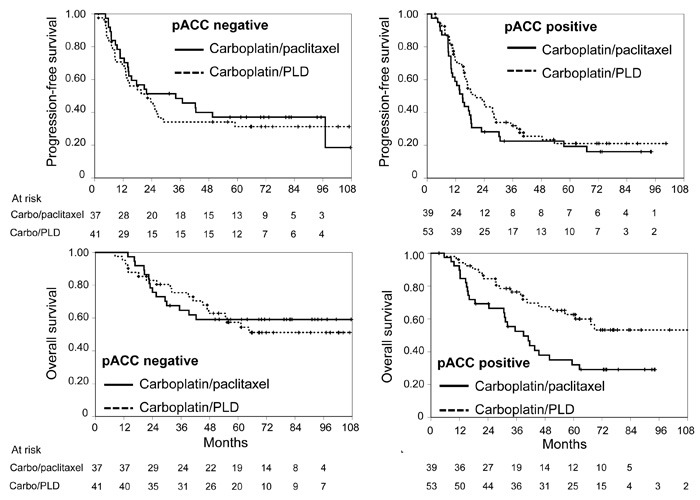
Kaplan-Meier estimated curves of progression-free survival (top) and overall survival (bottom) according to pACC status (negative: graphs on the left, positive: graphs on the right) Solid line: carboplatin/paclitaxel; dashed line: carboplatin/PLD. Vertical lines represent censoring.

### Biomarker analyses

Seventeen centres, enrolling 549 patients (67% of the whole study), supplied paraffin blocks from the primary tumor of 258 patients; 28 were excluded because tumor had been collected during interval debulking surgery after initiation of chemotherapy or date of collection was unknown. Therefore, 230 were eligible of whom 229 (42% of patients enrolled in the centres participating to the biomarker study and 28% of the whole study) had at least one biomarker tested and represented the biomarker population (Figure S2 online).

The biomarker population was similar to the overall study population, with a slightly lower rate of patients who were not operated at baseline and who had stage IV disease at diagnosis (Table S2 online). Consistently, both PFS and OS were slightly better in the biomarker population compared to the overall MITO2 population and to all patients enrolled in the centres participating to the biomarker study (Figure S3 online).

Distribution of biomarkers by treatment arm is reported in Table S3 online; due to core losses from the slides during IHC procedure, the number of cases tested ranged from 153 (pAMPK) to 223 (CDK6). No imbalances were observed between treatment arms at the planned significance level of 0.01.

Moderate pairwise associations (Table S4 online) were only found between pACC and either DNA-PK (Cramer's V = 0.487, *p* < 0.001) or pAMPK (Cramer's V = 0.433, *p* < 0.001).

There were no statistically significant associations at the level of 0.001 between patients' or tumors' characteristics and biomarkers' categories (Tables S5-S23 online) except for DNA-PK and residual disease (Table S23 online). Univariate PFS and OS Kaplan-Meier curves for each biomarker are depicted in Figures S4-S41 online.

No biomarker had statistically significant (predefined significance level of 0.01) prognostic value for PFS and OS at multivariable analyses (Table 1).

**Table 1:** Summary of multivariable analyses with candidate prognostic biomarkers

*Pathway*		Progression-free survival		Overall survival	
Biomarker		HR (95% CI)	*P* value	HR (95% CI)	*P* value
***Adhesion and invasion***					
ALCAM	membrane vs cytoplasm	1.16 (0.82-1.66)	0.40	0.84 (0.52-1.33)	0.46
MCAM	positive vs negative	0.93 (0.65-1.31)	0.66	0.79 (0.50-1.24)	0.31
CAV1					
Tumor	positive vs negative	0.90 (0.62-1.32)	0.60	0.76 (0.45-1.28)	0.30
Stroma	positive vs negative	1.01 (0.75-1.35)	0.97	0.82 (0.56-1.19)	0.29
Claudin3	high vs low	1.07 (0.77-1.50)	0.67	1.12 (0.73-1.70)	0.61
***Apoptosis***					
cFLIP	positive vs negative	0.97 (0.68-1.40)	0.87	1.00 (0.64-1.58)	0.99
TRAP	positive vs negative	0.85 (0.60-1.20)	0.35	0.77 (0.49-1.19)	0.24
BAG3			0.28		0.14
	2 vs 0-1	1.54 (0.91-2.62)		1.94 (0.997-3.76)	
	3 vs 0-1	1.23 (0.80-1.89)		1.22 (0.69-2.13)	
***Transcription regulation***					
HOX B13					
Cytoplasmic	positive vs negative	1.21 (0.86-1.69)	0.27	1.07 (0.70-1.64)	0.76
Nuclear	positive vs negative	1.09 (0.80-1.51)	0.58	1.34 (0.89-2.02)	0.16
HMGA2	2/3 vs 0/1	1.24 (0.85-1.81)	0.26	1.32 (0.82-2.10)	0.25
CDK6					
Intensity			0.82		0.11
	high vs low/moderate	0.88 (0.57-1.35)		0.60 (0.35-1.03)	
	very high vs low/moderate	0.97 (0.62-1.50)		0.94 (0.56-1.58)	
Cellular localization			0.21		0.20
	cytoplasm/membrane vs cytoplasm	1.40 (0.94-2.10)		1.60 (0.98-2.63)	
	cytoplasm/nucles vs cytoplasm	0.86 (0.44-1.65)		1.15 (0.52-2.56)	
***Metabolism***					
Leptin receptor			0.31		0.048
	10%/50% vs <10%	1.35 (0.83-2.21)		1.98 (1.10-3.56)	
	>50% vs <10%	0.92 (0.64-1.31)		0.95 (0.59-1.53)	

**Figure 2:**
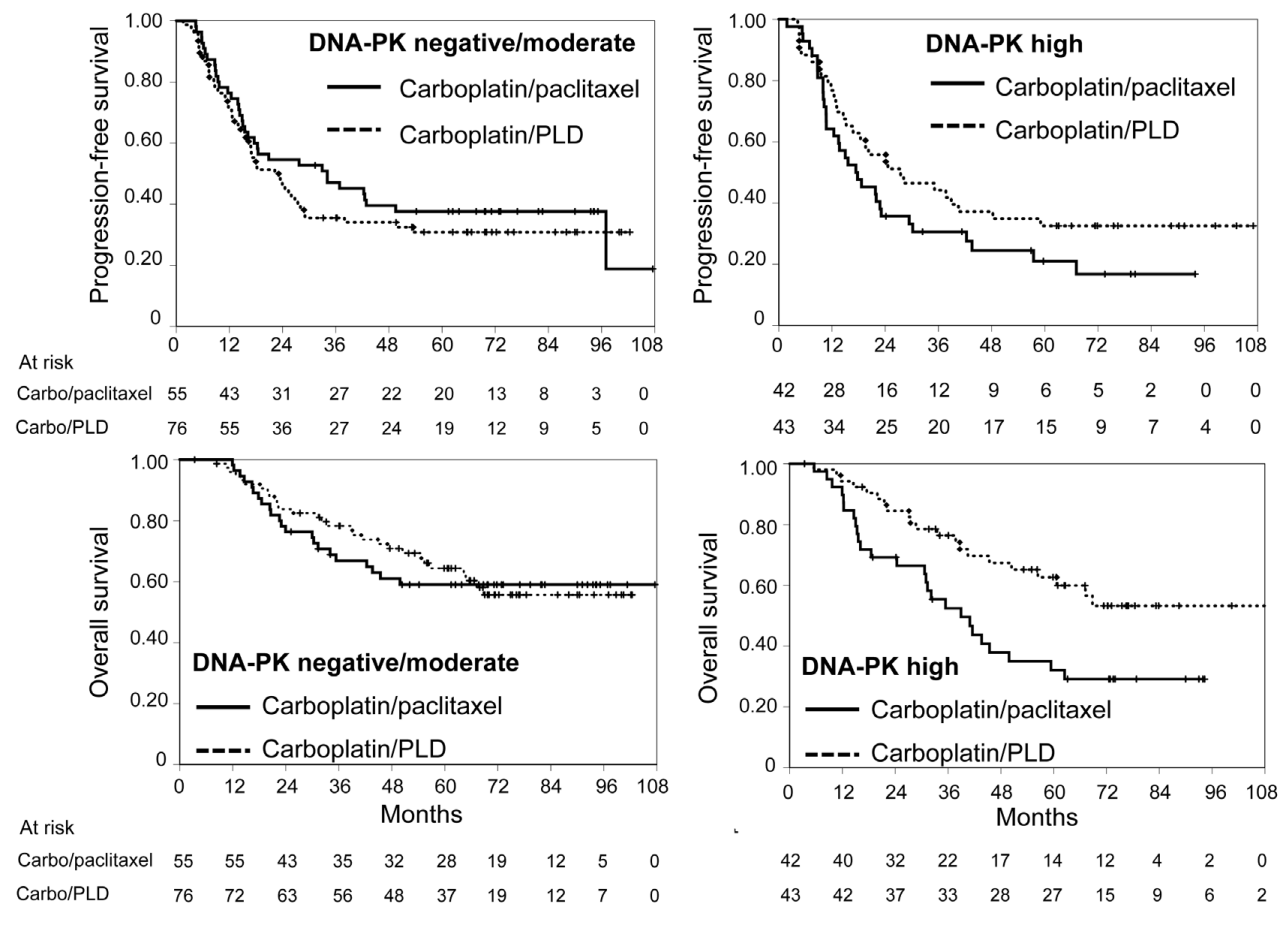
Kaplan-Meier estimated curves of progression-free survival (top) and overall survival (bottom) according to DNA-PK status (negative/moderate: graphs on the left, high: graphs on the right) Solid line: carboplatin/paclitaxel; dashed line: carboplatin/PLD. Vertical lines represent censoring.

At multivariable analyses of putative predictive biomarkers (Table 2), the predictive value role was confirmed for pACC (for both PFS and OS) and for DNA-PK (only for PFS). In both cases the presence of the biomarker appears to reduce the effect of the carboplatin/paclitaxel combination (Figures 1 and 2).

**Table 2:** Summary of multivariable analyses with candidate prognostic and predictive biomarkers

*Pathway*		Progression-free survival			Overall survival		
Biomarker		HR (95% CI)	*P* value	Interaction *P* value	HR (95% CI)	*P* value	Interaction *P* value
**Apoptosis**							
P53	high vs low	0.71 (0.51-0.99)	0.045	0.74	1.00 (0.64-1.58)	0.99	0.59
***Metabolism***							
pAMPK	positive vs negative	1.00 (0.67-1.50)	0.99	0.73	1.13 (0.67-1.89)	0.65	0.75
pACC	positive vs negative	1.15 (0.79-1.68)	0.47	**0.03**	1.21 (0.77-1.91)	0.41	**0.005**
	arm: carboplatin/paclitaxel	1.79 (1.00-3.20)			2.34 (1.20-4.58)		
	arm: carboplatin/PLD	0.77 (0.45-1.31)			0.69 (0.36-1.33)		
***DNA repair***							
Stathmin	high vs neg/moderate intensity	0.88 (0.62-1.25)	0.47	0.68	0.88 (0.55-1.41)	0.59	0.25
DNA-PK	high vs neg/moderate intensity	0.74 (0.52-1.06	0.10	**0.048**	0.88 (0.57-1.35)	0.55	0.09
	arm: carboplatin/paclitaxel	1.12 (0.65-1.95)					
	arm: carboplatin/PLD	0.51 (0.30-0.86)					

bold *P* values are considered as statistically significant

## DISCUSSION

We tried to find out molecular tumor characteristics that may ultimately be useful for prognostic prevision and to guide the choice of chemotherapy, within the MITO-2 randomized clinical trial of first-line chemotherapy for patients with AOC. None of 16 putative biomarkers had significant independent prognostic value; the expression of pACC and DNA-PK had a statistically significant interaction with treatment identifying patients who had less benefit from the carboplatin/paclitaxel combination as compared to the carboplatin/PLD one.

A biomarker analysis performed within a prospective clinical trial, with a common protocol for archival tumor samples management and statistical analysis independent of the laboratories (reducing risk of *ex-post* hypotheses adjustment), has a level of evidence immediately behind that of trials prospectively planned for biomarker analysis. [15] Nevertheless, our study has *a priori* limitations. First, previous evidence had several weaknesses, deriving from small, retrospective, selected, non-protocol-driven data sets. Second, tumor collection was not mandatory in MITO2, and an attrition bias was expected, that further increased due to random missing data produced by technical limitations of TMA technology; therefore, our study is powered for medium-large prognostic effects. Third, a conservative statistical approach was required to reduce the risk of false positive results coming from multiple testing.

**Figure 3:**
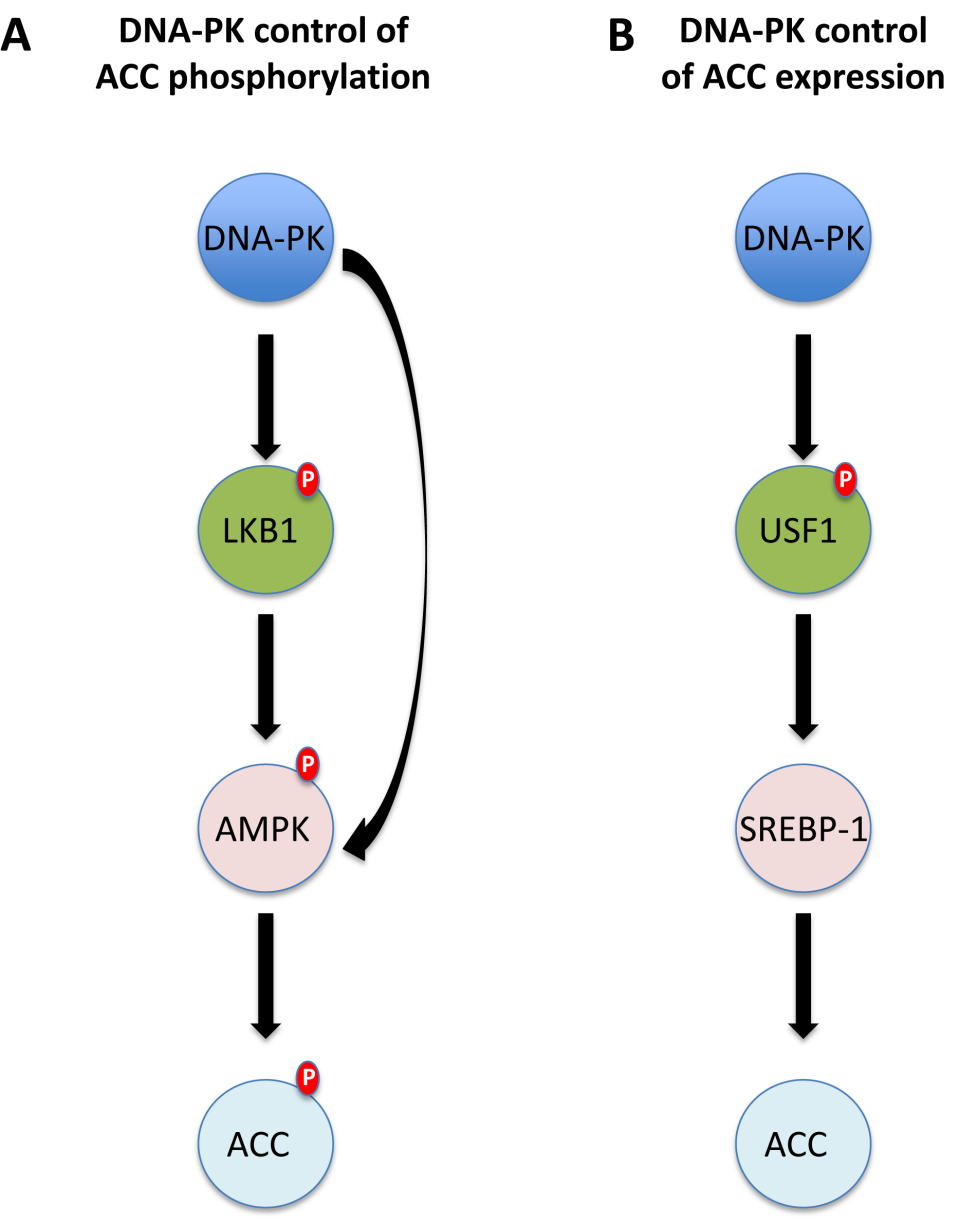
Possible molecular links between DNA-PKs and pACC expression **A.** DNA-PK could phosphorylate and activate AMPK directly, or indirectly *via* LKB1. AMPK activation eventually results in high ACC phosphorylation. **B.** DNA-PK phosphorylates the transcription factor USF1. This event is necessary for the proper expression of FAS (and ACC) by SREBP-1. Higher levels of pACC are in this case the consequence of higher levels of the total protein.

Two biomarkers, DNA-PK and pACC, had a statistically significant interaction with treatment, their overexpression being predictive of worse efficacy of the carboplatin/paclitaxel treatment. This is consistent with the hypothesis that high DNA-PK expression should identify patients more resistant to paclitaxel therapy. [16] DNA-PK and pACC expression have different functions, since DNA-PK is the catalytic subunit of a complex necessary for the Non-Homologous End Joining (NHEJ) DNA repair activity, [17] while ACC is the enzyme that converts acetyl-CoA in malonyl-CoA; the latter is phosphorylated and inhibited by AMP-activated protein kinase (AMPK), a sensor of cellular energy charge and a metabolic master switch. [18] In the present study, DNA-PK and pACC expression were correlated (Cramer's V = 0.487, *p* < 0.001) and there are at least two likely explanations for this correlation (Figure 3). First, DNA-PK could activate both LKB1 and AMPK, resulting in high ACC phosphorylation. [17, 19] Second, DNA-PK is a master regulator of fatty acids synthesis and its expression and activity is necessary for the proper regulation of the transcription factors USF1 and SERBP1 that control the expression of ACC and FAS (Fatty Acid Synthase). [20] In any case, it is now clear that DNA-PK is necessary for several biological functions other than the control of NHEJ DNA-repair and, among these, regulation of cellular metabolism *via* transcription regulation seems to be of primary relevance. [17, 21] AMPK also plays an important role in this setting; its activation, indeed, is necessary for the proper induction of doxorubicin-mediated death in several models, possibly through the control of autophagy; [22, 23] on the contrary, the same mechanism has been shown to protect cells from paclitaxel induced cell death, enhancing glycolysis during mitosis and preventing mitotic cell death. [24] These data strongly suggest that higher DNA-PK expression can prompt higher AMPK activity, causing higher pACC levels, and ultimately identifies ovarian cancer cells resistant to paclitaxel and sensitive to doxorubicin treatment.

Alternatively, it should be considered that paclitaxel action on interphase microtubules alters proteins transport and re-localization and may prevent nuclear localization of the enzymes involved in DNA-repair activity including DNA-PK, potentiating the activity of cytotoxic drug like platinum. [25-27] It is therefore possible that cells expressing high levels of DNA-PK are less sensitive to paclitaxel-induced DNA-PK cytoplasmic retention and more resistant to the effect of the carboplatin/paclitaxel combination.

In conclusion, we believe that our findings unveil a new pathway in ovarian cancer that might play an important role in the response of therapy. Additional work is needed to substantiate this hypothesis and to definitely clarify the involved mechanisms; also, further validation in larger series or prospective trials is warranted. DNA-PK and pACC might be first-in-kind biomarkers for personalizing the choice of chemotherapy regimen in ovarian cancer.

## MATERIALS AND METHODS

MITO2 is registered at clinicaltrials.gov NCT00326456. Trial details have been previously reported. [14] In the present long-term efficacy analysis, PFS and OS curves were reported according to the Kaplan-Meier method and were compared with the Log-rank test. Median estimates and HR, with 95% confidence intervals (CI) were also reported. All the analyses were performed according to intention-to-treat.

Following the approval of study amendments by Ethical Committee and patients' consent, tissue microarrays (TMA), prepared at the Pathology Unit of the NCI Naples (see page 5 online), were sent to seven laboratories involved in the study to test 16 putative biomarkers, some of which had more than one measurement, leading to 19 analyses. Biomarkers were classified according to their prevalent biological activity in 5 different groups: adhesion/invasion, apoptosis, transcription regulation, metabolism, DNA repair. Expected/hypothesized role and evidence or preliminary data for each studied biomarker are detailed online (pages 5-10 online). Methods for biomarker testing procedures are detailed in Table S1 online. Investigators involved in TMA preparation and analysis were blinded to the assigned treatment and patients' outcome.

The biomarker population was defined as the subgroup of patients for whom at least one biomarker result was available. Different two-tailed significance levels were applied to different biomarker analyses to partially adjust for multiple comparisons. Three planned conventional levels of P value, more or less conservative, were used according to the specific aim and the number of planned tests (0.001, 0.01, 0.05).

Baseline characteristics, and PFS and OS outcomes of the biomarker population were described, without statistical testing, to allow an informal comparison with the whole study population and the population of patients enrolled by the centres that participated in tumor block collection.

Association of biomarkers with baseline characteristics of the patients and the tumors were described and tested with the Chi-square test or the Wilcoxon rank sum test or the Kruskal-Wallis test, as appropriate, using a significance level of 0.001. PFS and OS curves were drawn with the Kaplan-Meier method.

Biomarkers were categorized in two or more levels according to predefined cut-offs. Pairwise associations between biomarkers were assessed by means of Cramer's V ranging from 0 (no association) to 1 (two variables fully concordant, give the same information). Statistical significance of pairwise associations was assessed with Chi-square test, using a significance level of 0.001.

The analysis of the prognostic role of each biomarker was adjusted by predefined clinical prognostic factors. Endpoints were PFS and OS. Each biomarker was added to a multivariable Cox's model with treatment arm, stage, residual disease, grading and age as covariates and prognostic effect was tested at a significance level of 0.01.

For P53, pAMPK, pACC, Stathmin, and DNA-PK previous data suggested a modifying effect on the treatment with taxane/anthracycline (see appendix online). Therefore, a predictive role had to be assessed and it was planned a priori to test the biomarker*treatment interaction, using the conventional significance level of 0.05. If the interaction test was not statistically significant the prognostic effect was evaluated as above.

## SUPPLEMENTARY MATERIALS FIGURES AND TABLES


