# Supplementary material for: Biomarker analysis of the MITO2 phase III trial of first-line treatment in ovarian cancer: predictive value of DNA-PK and phosphorylated ACC
Source: Oncotarget. 2016 Sep 15;7(45):72654–61. doi: 10.18632/oncotarget.12056 (PMC5341934; doi:10.18632/oncotarget.12056)
Supplement: Supplementary file 1 [file oncotarget-07-72654-s001.docx]

Biomarker analysis of the MITO2 phase III trial of first-line treatment in ovarian cancer: predictive value of DNA-PK and phosphorylated ACC

**Appendix**

Summary

[Long-term analysis of PFS and OS 4](#_Toc451945552)

[Figure S1: Progression-free and overall survival curves in the whole study population 4](#_Toc451945553)

[Preparation of tissue micro-array 5](#_Toc451945554)

[Biomarkers 5](#_Toc451945555)

[List of abbreviations 5](#_Toc451945556)

[ALCAM 5](#_Toc451945557)

[MCAM 5](#_Toc451945558)

[CAV1 6](#_Toc451945559)

[Claudin3 6](#_Toc451945560)

[p53 6](#_Toc451945561)

[cFLIP 7](#_Toc451945562)

[TRAP1 7](#_Toc451945563)

[BAG3 7](#_Toc451945564)

[HOXB13 8](#_Toc451945565)

[HMGA2 8](#_Toc451945566)

[CDK6 8](#_Toc451945567)

[Leptin receptor 9](#_Toc451945568)

[pAMPK 9](#_Toc451945569)

[pACC 9](#_Toc451945570)

[Stathmin 10](#_Toc451945571)

[DNA-PK 10](#_Toc451945572)

[Biomarker testing methods 11](#_Toc451945573)

[Table S1 11](#_Toc451945574)

[Biomarker population 14](#_Toc451945575)

[Figure S2. Flow of patients’ selection for biomarkers study. 14](#_Toc451945576)

[Table S2. Characteristics of 229 patients with at least 1 biomarker evaluable, compared with the whole MITO2 population 15](#_Toc451945577)

[Figure S3: Progression-free and overall survival curves in the whole study and the biomarker population 16](#_Toc451945578)

[Table S3. Distribution of biomarker reactivity by treatment arm 17](#_Toc451945579)

[Table S4 - Pairwise associations between biomarkers 19](#_Toc451945580)

[ALCAM 20](#_Toc451945581)

[Table S5.Characteristics of patients according to ALCAM category 20](#_Toc451945582)

[Figure S4. Progression-free survival 21](#_Toc451945583)

[Figure S5. Overall survival 22](#_Toc451945584)

[MCAM 23](#_Toc451945585)

[Table S6. Characteristics of patients according to MCAM category 23](#_Toc451945586)

[Figure S6. Progression-free survival 24](#_Toc451945587)

[Figure S7. Overall survival 25](#_Toc451945588)

[CAV1 (Tumor) 26](#_Toc451945589)

[Table S7. Characteristics of patients according to CAV1 (tumor) category 26](#_Toc451945590)

[Figure S8. Progression-free survival 27](#_Toc451945591)

[Figure S9. Overall survival 28](#_Toc451945592)

[CAV1 (Stroma) 29](#_Toc451945593)

[Table S8. Characteristics of patients according to CAV1 (stroma) category 29](#_Toc451945594)

[Figure S10. Progression-free survival 30](#_Toc451945595)

[Figure S11. Overall survival 31](#_Toc451945596)

[Claudin3 32](#_Toc451945597)

[Table S9. Characteristics of patients according to Claudin3 category 32](#_Toc451945598)

[Figure S12. Progression-free survival 33](#_Toc451945599)

[Figure S13. Overall survival 34](#_Toc451945600)

[p53 35](#_Toc451945601)

[Table S10. Characteristics of patients according to p53 category 35](#_Toc451945602)

[Figure S14. Progression-free survival 36](#_Toc451945603)

[Figure S15. Overall survival 37](#_Toc451945604)

[cFLIP 38](#_Toc451945605)

[Table S11. Characteristics of patients according to FLIP category 38](#_Toc451945606)

[Figure S16. Progression-free survival 39](#_Toc451945607)

[Figure S17. Overall survival 40](#_Toc451945608)

[TRAP1 41](#_Toc451945609)

[Table S12. Characteristics of patients according to TRAP1 category 41](#_Toc451945610)

[Figure S18. Progression-free survival 42](#_Toc451945611)

[Figure S19. Overall survival 43](#_Toc451945612)

[BAG3 44](#_Toc451945613)

[Table S13. Characteristics of patients according to BAG3 category 44](#_Toc451945614)

[Figure S20. Progression-free survival 45](#_Toc451945615)

[Figure S21. Overall survival 46](#_Toc451945616)

[HOXB13 (cytoplasm) 47](#_Toc451945617)

[Table S14. Characteristics of patients according to HOXB13 (cytoplasm) category 47](#_Toc451945618)

[Figure S22. Progression-free survival 48](#_Toc451945619)

[Figure S23. Overall survival 49](#_Toc451945620)

[HOXB13 (nucleus) 50](#_Toc451945621)

[Table S15. Characteristics of patients according to HOX B13 (nuclear expression) category 50](#_Toc451945622)

[Figure S24. Progression-free survival 51](#_Toc451945623)

[Figure S25. Overall survival 52](#_Toc451945624)

[HMGA2 53](#_Toc451945625)

[Table S16. Characteristics of patients according to HMGA2 category 53](#_Toc451945626)

[Figure S26. Progression-free survival 54](#_Toc451945627)

[Figure S27. Overall survival 55](#_Toc451945628)

[CDK6 (intensity) 56](#_Toc451945629)

[Table S17. Characteristics of patients according to CDK6 intensity category 56](#_Toc451945630)

[Figure S28. Progression-free survival 57](#_Toc451945631)

[Figure S29. Overall survival 58](#_Toc451945632)

[CDK6 (localization) 59](#_Toc451945633)

[Table S18. Characteristics of patients according to CDK6 localization category 59](#_Toc451945634)

[Figure S30. Progression-free survival 60](#_Toc451945635)

[Figure S31. Overall survival 61](#_Toc451945636)

[Leptin receptor 62](#_Toc451945637)

[Table S19. Characteristics of patients according to Leptin receptor category 62](#_Toc451945638)

[Figure S32. Progression-free survival 63](#_Toc451945639)

[Figure S33. Overall survival 64](#_Toc451945640)

[pAMPK 65](#_Toc451945641)

[Table S20. Characteristics of patients according to pAMPK category 65](#_Toc451945642)

[Figure S34. Progression-free survival 66](#_Toc451945643)

[Figure S35. Overall survival 67](#_Toc451945644)

[pACC 68](#_Toc451945645)

[Table S21. Characteristics of patients according to pACC category 68](#_Toc451945646)

[Figure S36. Progression-free survival 69](#_Toc451945647)

[Figure S37. Overall survival 70](#_Toc451945648)

[Stathmin 71](#_Toc451945649)

[Table S22. Characteristics of patients according to Stathmin category 71](#_Toc451945650)

[Figure S38. Progression-free survival 72](#_Toc451945651)

[Figure S39. Overall survival 73](#_Toc451945652)

[DNA-PK 74](#_Toc451945653)

[Table S23. Characteristics of patients according to DNA-PK category 74](#_Toc451945654)

[Figure S40. Progression-free survival 75](#_Toc451945655)

[Figure S41. Overall survival 76](#_Toc451945656)

[Investigators who participated in MITO2 77](#_Toc451945657)

[Writing committee (alphabetical order) 77](#_Toc451945658)

[Centralization, preparation and validation of TMA 77](#_Toc451945659)

[Analysis of ALCAM, MCAM, CAV1, cFLIP 77](#_Toc451945660)

[Analysis of Stathmin, DNA-PK, CDK6 77](#_Toc451945661)

[Analysis of pAMPK, pACC 77](#_Toc451945662)

[Analysis of p53 77](#_Toc451945663)

[Analysis of TRAP1 77](#_Toc451945664)

[Analysis of HMGA2, BAG3 77](#_Toc451945665)

[Analysis of Claudin3, Leptin receptor, HOXB13 77](#_Toc451945666)

[Statistical analysis 77](#_Toc451945667)

[Pathologists 77](#_Toc451945668)

[Clinical Investigators 77](#_Toc451945669)

[Data managers 78](#_Toc451945670)

[Research nurses 78](#_Toc451945671)

[References 79](#_Toc451945672)

# Long-term analysis of PFS and OS

##
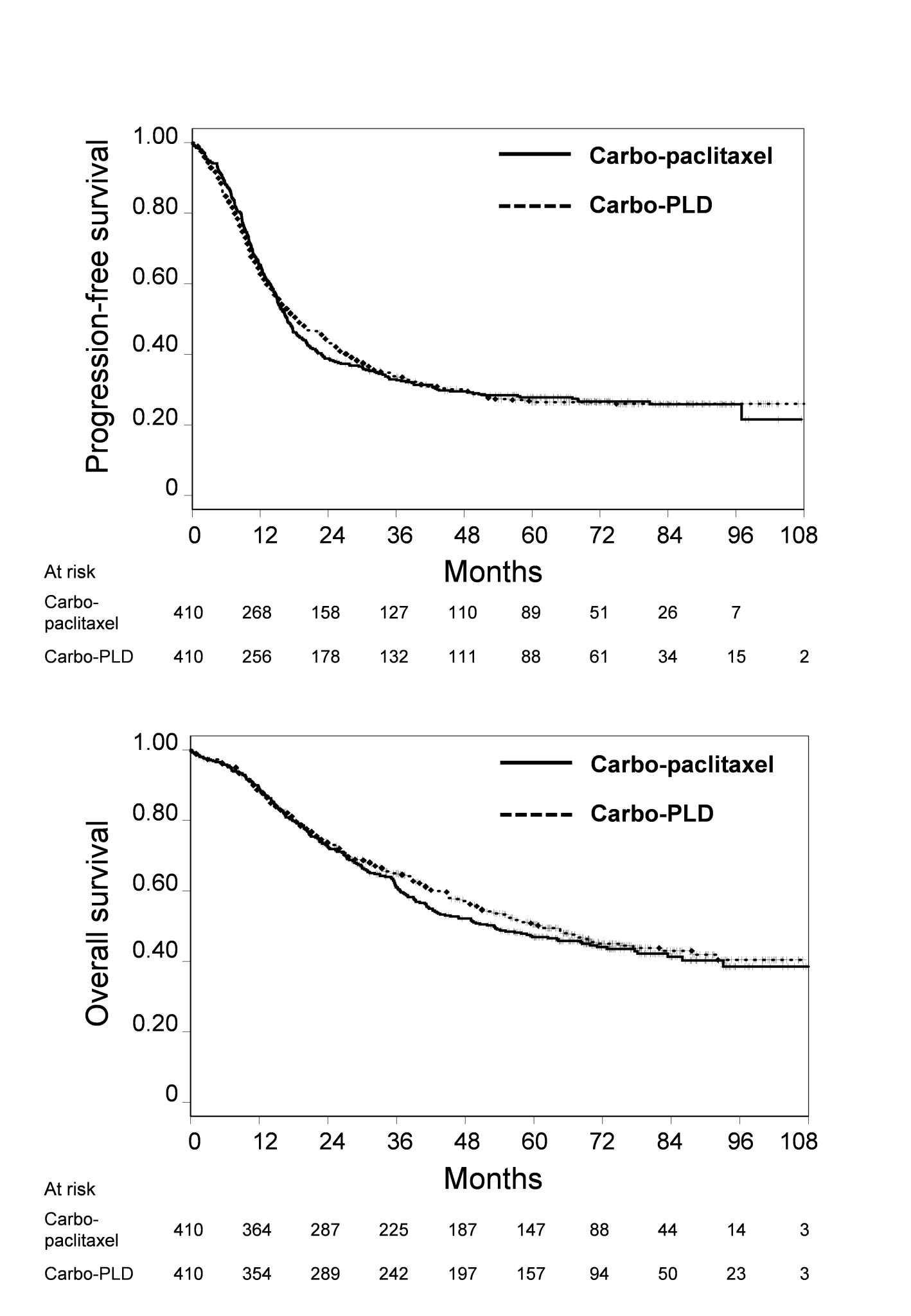
Figure S1: Progression-free and overall survival curves in the whole study population

# Preparation of tissue micro-array

Tissue micro-array (TMA) was built taking the most representative areas from each single case. Whole hematoxylin and eosin stained (H&E) section from each tumor block was carefully examined by the pathologist. The selected areas of interest presented more than 50% of cancer cells, without necrosis. Two cores (1 mm) were collected from each of the 230 eligible tumor blocks and arrayed into a recipient paraffin block (35 mm×20 mm) using a semiautomatic tissue arrayer instrument (Galileo CK3500 TMA, ISENET, Milan, Italy).[[1](#_ENREF_1), [2](#_ENREF_2)] At the end, six recipient blocks were constructed and cut into 4 μm thick sections. The presence of tumour cells on the arrayed specimens was verified using H&E staining.

# Biomarkers

## List of abbreviations

ALCAM = activated leukocyte cell adhesion molecule

MCAM = melanoma cell adhesion molecule

CAV1 = Caveolin1

cFLIP = cellular FLICE-like inhibitory protein

TRAP1 = TNF receptor-associated protein

HMGA2 = High Mobility Group A 2

BAG3 = Bcl2-associated athanogene 3

HOX-B13 = homeobox B13

CDK6 = Cyclin-dependent kinase 6

pAMPK = phospho AMP-activated protein kinase

DNA-PK = DNA-dependent protein kinase catalytic subunit

pACC = phospho acetyl-CoA carboxylases

## ALCAM

*Function*

Member of the immunoglobulin-like cell-adhesion superfamily, it promotes heterophilic (ALCAM-CD6) and homophilic (ALCAM-ALCAM) cell-cell interactions being involved in cell adhesion, cell migration and guided outgrowth in neurogenesis, hematopoiesis and tumor progression.

*Expected/hypothesized role in ovarian cancer*

ALCAM is highly expressed on the membrane of ovarian cancer cell lines having a role in rearrangement of cell-cell contacts and impacting on cell motility. Our hypothesis is that a loss of ALCAM membrane expression may be associated to tumor progression.

*Evidence or preliminary data*

Loss of ovarian cancer cell anchorage is accompanied by a loss of ALCAM expression at membrane level in both cell lines and primary ovarian tumor cells derived from ovarian cancer patients’ ascites. Decrease/loss of ALCAM membrane expression resulted a marker of poorer outcome in EOC patients. [[3-6](#_ENREF_3)]

## MCAM

*Function*

Member of the immunoglobulin-like cell-adhesion superfamily, MCAM is a Ca2+-independent cell adhesion molecule that plays an important role in implantation and placentation during embryogenesis and in tumor progression.

*Expected/hypothesized role in ovarian cancer*

MCAM is expressed on ovarian cancer cells and its expression appears related to ovarian cancer progression. We hypothesized a possible MCAM involvement in tumor prognosis.

*Evidence or preliminary data*

MCAM expression was found significantly associated with advanced tumor stage, serous and undifferentiated histotype and p53 protein accumulation. In the subgroup of advanced-stage patients responding to front-line therapy, MCAM expression was a marker stronger than residual disease in predicting early tumor relapse and an independent marker of poor prognosis.[[7](#_ENREF_7)]

## CAV1

*Function*

Structural component of the caveolae, it plays dual role in cancer. In early stages of disease the protein functions predominantly as a tumor suppressor by negatively regulating intracellular signaling, whereas at later stages, CAV1 expression is associated with tumor progression and metastasis. A role as regulator of tumor- surrounding tissue remodelling and desmoplastic processes has been proposed too.

*Expected/hypothesized role in ovarian cancer*

CAV1 protein is low/absent in many ovarian cancer *in vitro* models but its expression is induced after treatment with platinum or taxane and it is highly expressed in the intrinsically resistant ones. We hypothesized that re-expression of CAV1 protein in ovarian cancer is associated with a more aggressive and drug resistant disease.

*Evidence or preliminary data*

Preliminary analysis of a consecutive series of 207 cases of ovarian cancer revealed a quite heterogeneous expression of the protein and its association with unfavorable prognosis.[[8](#_ENREF_8)]

## Claudin3

*Function*

The claudin family consists of 27 members with different tissue-specific expression. Claudin3 has been reported to be more expressed in ovarian carcinoma compared to normal ovarian surface epithelium.

*Expected/hypothesized role in ovarian cancer*

We hypothesized that Claudin3 low expression could be related to poor prognosis, due to the loss of cell-cell adhesion.

*Evidence or preliminary data*

Conflicting results have been reported about the role of Claudin3 in ovarian cancer progression.[[9](#_ENREF_9)] In fact, low Claudin3 protein expression was associated with a trend toward poorer survival in an analysis of 115 primary ovarian carcinomas.[[10](#_ENREF_10)] In contrast, in another study high Claudin3 expression in ovarian serous adenocarcinoma is related with shorter survival.[[11](#_ENREF_11)] Interestingly it has been reported that deregulation of Claudin3 in ovarian cell cancer improves activity of cisplatin *in vitro*.[[12](#_ENREF_12)]

## p53

*Function*

The tumor suppressor gene p53 is frequently mutated in ovarian cancer, especially in high grade serous carcinoma. In high percentage of cases p53 mutation confer to the protein additional oncogenic functions and in these cases p53 usually resulted overexpressed.[[13](#_ENREF_13)]

*Expected/hypothesized role in ovarian cancer*

We hypothesized that ovarian cancer over-expressing p53 (i.e. the tumors expressing high levels of p53 in more that 30% of the cells) represented a subgroup with specific features in tumor spreading and response to therapy likely due to the described gain of function of p53 missense mutants. The cut off was chosen based on previous publications to identify cases with mutant p53 gain of function. [[14-16](#_ENREF_14)]

*Evidence or preliminary data*

As a master regulator of proliferation, p53 has been described to interact with many other oncologic relevant markers including some of the putative biomarkers here investigated. In particular, the long isoform of cFLIP (cFLIPL) was identified as directly involved in the impairment of apoptotic signaling in the context of functional p53;[[17](#_ENREF_17)] while mutant p53 expression correlates with the expression of stathmin and DNA-PK and, in the presence of mutant p53 (i.e. high p53 expression in ≥30% of tumor cells), DNA-PK and/or stathmin are a marker of poor prognosis and therapy response.[[18](#_ENREF_18)]

## cFLIP

*Function*

The cellular FLICE inhibitory protein (cFLIP) interferes with efficient death-inducing signaling complex formation by inhibiting caspase8 recruitment and processing during extrinsic apoptotic pathway execution.

*Expected/hypothesized role in ovarian cancer*

cFLIP inhibition is relevant in receptor-dependent apoptosis and in sensitizing cancer cells to conventional chemotherapy. We hypothesized a prognostic and/or predictive role for cFLIP expression.

*Evidence or preliminary data*

We identified cFLIPL expression as an independent prognostic factor for adverse outcome in a series of 207 ovarian cancer patients. [[17](#_ENREF_17), [19-21](#_ENREF_19)]

## TRAP1

*Function*

TRAP1, the mitochondrial isoform of HSP90, is a component of a mitochondrial pathway selectively up-regulated in tumor cells. TRAP1 is an antiapoptotic chaperon involved in stress-adaptive response of cancer cells.[[22](#_ENREF_22)]

*Expected/hypothesized role in ovarian cancer*

Our group has previously demonstrated TRAP1-dependent attenuation of protein synthesis and cell migration in cancer cells and tissues.[[23](#_ENREF_23)] A role of TRAP1 in ovarian cancer is expected since TRAP1 downregulation increases at translational level the expression of p70S6K, a kinase involved in the control of cell migration in ovarian cancer.

*Evidence or preliminary data*

Increased expression of TRAP1 has been observed in several cancer types and our group has previously demonstrated TRAP1-dependent attenuation of protein synthesis and cell migration in cancer cells and tissues. However, preliminary evidence shows a significant inverse correlation with tumor stage in ovarian, cervical and bladder cancer. These observations candidate TRAP1 and its protein “signature” as promising biomarkers in OC, opening new scenarios to link chemoresistance to metastatic potential and metabolic remodelling.[[24](#_ENREF_24)]

## BAG3

*Function*

The antiapoptotic protein BAG3, member the HSP70 co-chaperones family, has been shown to play a relevant role in the survival, growth and invasiveness of different tumor types.[[25-28](#_ENREF_25)]

*Expected/hypothesized role in ovarian cancer*

BAG3 protein sustains anaplastic thyroid cancer growth *in vitro* and *in vivo*. The underlying molecular mechanism appears to rely on BAG3 binding to BRAF, thus protecting it from proteasome-dependent degradation.[[29](#_ENREF_29), [30](#_ENREF_30)] We hypothesized a prognostic role for BAG3 protein expression.

*Evidence or preliminary data*

Our *in vitro* unpublished data show that BAG3 play a role in epithelial-mesenchymal transition of ovarian cancer cells.

## HOXB13

*Function*

HOXB13 belongs to the Homeobox gene family and is normally involved in epidermal differentiation. Moreover, it is overexpressed in several tumors such as prostate, endometrial, ovarian, cervical, and breast cancer and transitional cell tumor, suggesting its contribution to carcinogenesis and tumor progression.[[31](#_ENREF_31)]

*Expected/hypothesized role in ovarian cancer*

HOXB13 promotes ovarian cell lines invasion. Thus, we hypothesized that HOXB13 overexpression could be related to more aggressive ovarian cancer.

*Evidence or preliminary data*

HOXB13 promotes cell proliferation and anchorage-independent growth in mouse ovarian cancer cell lines that contain genetic alterations in p53, myc, and ras and it confers resistance to tamoxifen-mediated apoptosis.[[32](#_ENREF_32)] In addition, *in vitro* data indicate that upon HOXB13 antisense introduction, ovarian cancer cells show reduced invasion ability.[[33](#_ENREF_33)]

## HMGA2

*Function*

HMGA2 is a small, non-histone, chromatin-associated protein with a key role in tumorigenesis and adipogenesis. Indeed, HMGA2 overexpression has been frequently detected in several malignant neoplasms. The role of HMGA2 protein in cell transformation is essentially based on its ability to down- or up- regulate expression of genes that have a critical role in control of cell proliferation and invasion.

*Expected/hypothesized role in ovarian cancer*

We hypothesized that HMGA2 might be a negative prognostic factor.

*Evidence or preliminary data*

HMGA2 is overexpressed in high-grade papillary serous carcinoma and overexpression correlates with an adverse prognosis.[[34](#_ENREF_34), [35](#_ENREF_35)] Our preliminary data on 117 EOC patients demostrated that HMGA2 overexpression and BMI can aid in the clinical management of EOC by identifying a subset of patients with poor prognosis.[[36](#_ENREF_36)]

## CDK6

*Function*

CDK6 is a cyclin dependent kinase involved in the regulation of G1/S phase transition. It directly phosphorylates RB eventually regulating its transcriptional actvity.[[37](#_ENREF_37)] It is frequently altered in human sarcomas and leukemia. It may have additional functions directly related to gene transcription.[[38](#_ENREF_38)]

*Expected/hypothesized role in ovarian cancer*

Since in ovarian cancer CDK6 is mainly located into the cytoplasm, we hypothesized that its subcellular distribution could be associated with different functions respect to the control of G1 to S phase transition (considered the typical function of nuclear CDK6/cyclin D complex).

*Evidence or preliminary data*

Our preliminary data and published data suggest a role for CDK6 in regulating platinum sensitivity through a transcriptional regulation.[[39](#_ENREF_39), [40](#_ENREF_40)]

## Leptin receptor

*Function*

Interest is growing on the potential role of adipokines in the development of gynecological cancer. It seems that a role in this scenario is played by interaction between the adipokine leptin, produced in higher quantity in white adipose tissue *in vivo*, and its receptor.[[41](#_ENREF_41)]

*Expected/hypothesized role in ovarian cancer*

We hypothesized that the expression of the leptin receptor is related to a poor prognosis.

*Evidence or preliminary data*

*In vitro* studies on human cell lines of ovarian and endometrial cancer indicate that leptin leads to the activation of signaling pathways, such as JAK2/STAT3, MAPK/ERK and PI3K/AKT, which stimulate cell proliferation and inhibit apoptosis.[[41](#_ENREF_41)] In our preliminary data, Leptin receptor shows a variable expression in ovarian cancer, being expressed at higher levels in serous-papillary carcinoma.

## pAMPK

*Function*

AMPK is a heterotrimeric serine/threonine protein kinase, and is activated following its phosphorylation by its upstream kinase LKB1 in response to an increase in cellular AMP/ATP ratio.[[42](#_ENREF_42)] AMPK plays a central role in the regulation of energy metabolism in all eukaryotes, and governs glucose and lipid metabolism in response to alterations in nutrients and intracellular energy levels, contributing to maintain the steady-state levels of intracellular ATP.[[43](#_ENREF_43)] AMPK is also emerging as a novel genomic stress sensor, which participates in the DNA damage response pathway.[[44](#_ENREF_44)] This might connect AMPK activation (and thus ACC phosphorylation) with DNA-PK expression and resistance to taxanes. [[45](#_ENREF_45)] On the other hand, AMPK activation is necessary for the proper induction of doxorubicin-mediated death in several models, likely through the control of autophagy. [[46](#_ENREF_46)]

*Expected/hypothesized role in ovarian cancer*

In line with the tumor suppressor role of LKB1/AMPK axis described in several studies investigating the prognostic role of AMPK in human malignancies,[[47](#_ENREF_47)] we hypothesized that in ovarian cancer low levels of AMPK activation could correlate with poor prognosis.[[48](#_ENREF_48)]

*Evidence or preliminary data*

A previous study investigated the prognostic impact of AMPK expression in ovarian carcinoma in a small cohort of 70 patients.[[48](#_ENREF_48)] Results suggest that loss of AMPK expression is associated with shorter overall survival in the subgroup of patients with serous ovarian carcinoma and in advanced tumor stages.

## pACC

*Function*

Phosphorylation of ACC by AMPK contributes to regulation of fatty-acid metabolism and is considered an indirect read-out of AMPK activation.[[43](#_ENREF_43)] AMPK is a major regulator of fatty acid catabolism through phosphorylating and inactivating ACC1 and ACC2 that catalyze acetyl-CoA carboxylation to produce malonyl-CoA, a substrate for fatty acid biosynthesis. In addition, AMPK-dependent ACC1/2 inactivation increases NADPH levels due to its decreased consumption during fatty acid synthesis and increased NADPH production through β-oxidation.[[49](#_ENREF_49)]

*Expected/hypothesized role in ovarian cancer*

We hypothesized that the cooperative effects of increased expression of pAMPK and pACC, by supplying energy source and by providing protection from ROS production, could contribute to increased cell survival.

*Evidence or preliminary data*

Previous *in vitro* studies disclosed that ACC phosphorylation is associated with modulation of taxol-induced ovarian cancer cell death.[[50](#_ENREF_50)]

## Stathmin

*Function*

Stathmin is a microtubules destabilizing protein overexpressed in many type of cancer including ovarian cancer.[[51](#_ENREF_51)] In ovarian cancer its expression correlated with the expression of DNA-PK that is necessary for a proper response to platinum.[[18](#_ENREF_18)] High stathmin expression has been related to the resistance of ovarian cancer cells to taxanes.[[39](#_ENREF_39), [52](#_ENREF_52)]

*Expected/hypothesized role in ovarian cancer*

We hypothesized that a high expression of stathmin, especially in the context of p53 mutation, could identify ovarian cancer patients with poor prognosis and resistance to the platinum-paclitaxel combination. Moreover, we expected that stathmin expression should tightly correlate with DNA-PK expression and share the same prognostic/predictive role, although, based on the proposed mechanism of action, DNA-PK should represent a more promising biomarker.[[18](#_ENREF_18)]

*Evidence or preliminary data*

Evidence already reported include: the prognostic role of stathmin;[[18](#_ENREF_18), [39](#_ENREF_39)] its co-expression with p53 and DNA-PK;[[18](#_ENREF_18), [53](#_ENREF_53)] the role of stathmin in DNA damage response and resistance to taxanes.[[18](#_ENREF_18), [39](#_ENREF_39), [51](#_ENREF_51), [52](#_ENREF_52)]

## DNA-PK

*Function*

DNA-PK catalytic subunit (DNA-PKcs here referred as DNA-PK) is the master regulator of Non-Homologous End Joining (NHEJ) DNA repair activity following double strand breaks both in dividing and non dividing cells.[[54](#_ENREF_54)]

*Expected/hypothesized role in ovarian cancer*

We hypothesized that: a high expression of DNA-PK, especially in the context of p53 mutation, could identify ovarian cancer patients with poor prognosis and resistance to the platinum-paclitaxel combination. DNA-PK should tightly correlate with stathmin expression and share the same prognostic/predictive role.

*Evidence or preliminary data*

DNA-PK participates to the response to platinum in ovarian cancer where it regulates the stability and transcriptional activity of mutant p53 in concert with stathmin.[[18](#_ENREF_18)] Its co-expression with p53 and stathmin and the role of DNA-PK in DNA damage response and resistance to taxanes have been also described.[[18](#_ENREF_18), [54](#_ENREF_54)]

# Biomarker testing methods

## Table S1

| **Biomarker** | **Antibody Clone, company** | **Dilution** | **Antigen retrieval** | **Secondary antibody** | **Immunohistochemistry reading and scoring (references)** |
| --- | --- | --- | --- | --- | --- |
| **ALCAM** | Mouse anti-ALCAM (CD166), Novocastra | 1:80 | Citrate buffer 10mM pH6, at 121°C for 2min | biotinylated anti-mouse (DAKO) 1:100 + streptavidin DAKO 1:300 | Slides were evaluated by two independent observers blinded to patient characteristics. Staining was recorded by a subjective grading system, considering both staining localization (membranous versus cytoplasmic) and the proportion of cells showing a membrane-positive reaction, as reported.[[4](#_ENREF_4)] Images were acquired and stored by APERIO SCAN SCOPE xT software (Leica Microsystems, Germany). |
| **MCAM** | Mouse anti-MCAM (CD146), Novocastra | 1:50 | Citrate buffer 10mM pH6, at 96°C for 6 min | biotinylated anti-mouse (DAKO) 1:100 + streptavidin DAKO 1:300 | Slides were evaluated by two independent observers blinded for clinical and biochemical data. A final IHC score (negative/positive) was obtained as reported.[[7](#_ENREF_7)] Images were acquired and stored by APERIO SCAN SCOPE xT. |
| **CAV1 (tumor)** | Rabbit anti Cav1 (N20), Santa Cruz | 1ug/ml | no | biotinylated anti rabbit (DAKO)1:200 + streptavidin DAKO 1:300 | Slides were evaluated by two independent observers blinded to patient characteristics. Staining of each biopsy sample was evaluated as previously described.[[8](#_ENREF_8)] A final IHC score was obtained by multiplying the intensity and the percentage scores. Tumors were considered positive for caveolin expression if they had a score >3. Images were acquired and stored by APERIO SCAN SCOPE xT. |
| **CAV1 (stroma)** | Rabbit anti Cav1 (N20), Santa Cruz | 1ug/ml | no | biotinylated anti rabbit (DAKO)1:200 + streptavidin DAKO 1:300 | Staining intensity of fibroblast cells was evaluated as undetectable, faint, moderate or intense. A final IHC score was obtained by multiplying the intensity and the percentage scores. Tumors were considered positive for caveolin expression if they had a score >3. Images were acquired and stored by APERIO SCAN SCOPE xT. |
| **Claudin3** | Ab15102, AbCAM | 1:100 | Tris-EDTA-buffer pH7a.8 in pressure cooker for 30 min | Visualization Reagent (Dako SK001) | Tumors with less than 5% claudin3 expression were considered negative regardless of the intensity described. The intensity of the claudin3 staining was classified into four grades: negative and weak (coded as “low”) and moderate and strong (coded as “high”).[[55](#_ENREF_55)] |

Table S1 - continued

| **Biomarker** | **Antibody Clone, company** | **Dilution** | **Antigen retrieval** | **Secondary antibody** | **Immunohistochemistry reading and scoring (references)** |
| --- | --- | --- | --- | --- | --- |
| **p53** | Mouse monoclonal antibody against p53 (clone DO7) (DAKO) | 1:100 | Citrate buffer 10 mM pH6, at 96 C° for 15 min | biotinylated goat antibody anti-mouse IgG + ABC complex (DAKO) | IHC reaction was interpreted by an expert pathologist. p53 positive tumors were defined as the tumors in which at least 30% cells displayed a strong nuclear p53 staining.[[14](#_ENREF_14)] |
| **cFLIP** | Goat anti-FLIP (C19), Santa Cruz | 2ug/ml | Citrate buffer 10mM pH6, at 121°C for 2 min | biotinylated anti-goat DAKO 1:200 + streptavidin DAKO 1:300 | Slides were evaluated by two independent observers blinded to patient characteristics. Tumors were considered positive for c-FLIP-L expression if they had a score equal/greater than 3 as reported.[[7](#_ENREF_7), [19](#_ENREF_19)] Images were acquired and stored by APERIO SCAN SCOPE xT. |
| **TRAP1** | Mouse anti-HSP 75(TR-1A), Santa Cruz | 1:100 | Citrate buffer 10mM pH6 at 96°C for 15 min | biotinylated goat anti-rabbit/anti-mouse IgG (Dako LSAB2 System) | IHC reaction was interpreted by an expert pathologist and the final score obtained taking into account the percentage of positive cells (Score 0-6) and the intensity of the staining (score 0-3).[[24](#_ENREF_24)] Scores≥ 1were defined as “positive”. |
| **BAG3** | Rabbit anti BAG3 (TOS2), Alexis Biochemicals | 1:200 | no | biotinylated goat anti-rabbit/anti-mouse IgG (Dako LSAB2 System) | IHC reaction was interpreted by an expert patholgist and the final score obtained taking into account the percentage of positive cells (score 0-3).[[56](#_ENREF_56)] |
| **HOXB13** | Ab53931, AbCAM | 1:450 | Tris-EDTA-buffer pH7.8 in pressure cooker for 30 min | EnVision Flex (Dako K8002):  I)/Mouse Linker II)/HRP | The intensity of the HOXB13 staining was classified into one of four grades: absent and weak (coded as “negative”) and moderate and strong (coded as “positive”). Tumors with less than 5% HOXB13 expression were considered negative regardless of the intensity described.[[57](#_ENREF_57)] |
| **HMGA2** | Rabbit anti-HMGA2-P1, BioCheck | 1:300 | Citrate buffer 10 mM pH 6 at 96°C for 15 min | Biotinylated goat anti-rabbit/anti-mouse IgG (Dako LSAB2 System) | Staining was considered positive when localized to the nucleus. Staining extent was scored on a scale of 0-3, as follows: 0=no staining, 1=1-10% , 2=11-49%, 3=50-100% stained tumor cells as previously reported.[[36](#_ENREF_36)] |
| **CDK6** | Rabbit anti-CDK6 (C21), Santa Cruz | 1:800 | Citrate buffer 10 mM pH6 at 96°C for 15 min | Biotinylated goat anti-rabbit/anti-mouse IgG (Dako LSAB2 System) | IHC reaction was interpreted by an expert pathologist and the final intensity score was obtained taking into account the percentage of positive cells (score 0-4) and the intensity of the staining (score 1-3) as previously reported.[[58](#_ENREF_58)] Scores <6 were coded as “low/moderate”, 6 as “high”, 7 as “very high”. |

Table S1 - continued

| **Biomarker** | **Antibody Clone, company** | **Dilution** | **Antigen retrieval** | **Secondary antibody** | **Immunohistochemistry reading and scoring (references)** |
| --- | --- | --- | --- | --- | --- |
| **Leptin receptor** | Ab60042, AbCAM | 1:350 | Tris-EDTA-buffer pH7.8 in pressure cooker for 30 min | Visualization Reagent (Dako SK001) | The expression of leptin receptor in cancer samples was classified using a four-point scale: <10% positive cells; 10 to 50% positive cells with weak staining; >50% positive cells with weak staining; >50% positive cells with strong staining.[[59](#_ENREF_59)] |
| **pAMPK** | #2535, Cell Signaling Technology | 1:150 | EDTA 1 mM pH8 at 96°C for 45 min | Biotinylated anti-rabbit antibody for 30 min, followed by Vectastain® ABC reagent (Vector Labs, Peterborough, U.K.) for additional 30 min. | IHC reaction was interpreted by an expert pathologist and the final score obtained taking into account both the percentage of positive cells and intensity of the staining.[[47](#_ENREF_47)] Tumor samples were considered positive for pAMPK expression if they had a score >5. |
| **pACC** | #3661, Cell Signaling Technology | 1: 100 | EDTA 1 mM pH8 at 96°C for 45 min | Biotinylated anti-rabbit antibody for 30 min, followed by Vectastain® ABC reagent (Vector Labs, Peterborough, U.K.) for additional 30 min. | IHC reaction was interpreted by an expert pathologist and the final score obtained taking into account both the percentage of positive cells and intensity of the staining.[[47](#_ENREF_47)] Tumor samples were considered positive for pACC expression if they had a score >5. |
| **Stathmin** | Rabbit anti-stathmin, Cell Signaling Technology | 1:50 | Citrate buffer 10 mM pH6 at 96°C for 15 min | Biotinylated goat anti-rabbit/anti-mouse IgG (Dako LSAB2 System) | IHC reaction was interpreted by an expert pathologist and the final score obtained taking into account the percentage of positive cells (Score 0-4) and the intensity of the staining (score 1-3).[[18](#_ENREF_18), [58](#_ENREF_58)] A score <5 was defined as “negative/moderate intensity”; ≥5 as “high intensity”. |
| **DNA-PK** | Mouse anti DNA-PKcs (42-psc), Neomarkers | 1:800 | Citrate buffer 10 mM pH6 at 78°C for 24 h | Biotinylated goat anti-rabbit/anti-mouse IgG (Dako LSAB2 System) | IHC reaction was interpreted by an expert pathologist and the final score obtained taking into account the percentage of positive cells (Score 0-4) and the Intensity of the staining (score 1-3).[[58](#_ENREF_58)] A score <5 was defined as “negative/moderate intensity”; ≥5 as “high intensity”. |

# Biomarker population

## Figure S2. Flow of patients’ selection for biomarkers study.

##
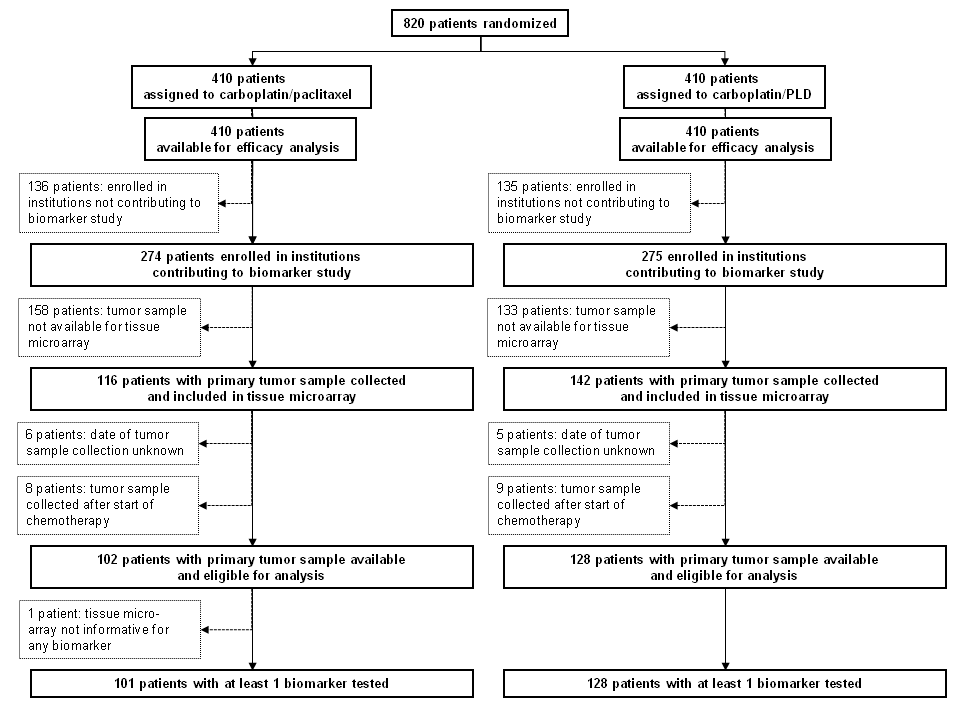


## Table S2. Characteristics of 229 patients with at least 1 biomarker evaluable, compared with the whole MITO2 population

|  | **Patients with ≥1 biomarker assessed**  **(n=229)** | | **Centers**  **with biopsies**  **(n=549)** | | **Whole**  **study**  **(n=820)** | |  |
| --- | --- | --- | --- | --- | --- | --- | --- |
| **Age** |  |  |  |  |  |  |  |
| median (range) | 57 | (28-77) | 57 | (25-77) | 57 | (21-77) |  |
| < 70 yrs | 200 | (87%) | 486 | (89%) | 721 | (88%) |  |
| > 70 yrs | 29 | (13%) | 63 | (11%) | 99 | (12%) |  |
| **ECOG performance status** |  |  |  |  |  |  |  |
| 0-1 | 222 | (97%) | 529 | (96%) | 795 | (97%) |  |
| 2 | 7 | (3%) | 20 | (4%) | 25 | (3%) |  |
| **Residual disease** |  |  |  |  |  |  |  |
| None | 94 | (41%) | 220 | (40%) | 298 | (36%) |  |
| ≤ 1 cm | 50 | (22%) | 94 | (17%) | 149 | (18%) |  |
| > 1 cm | 65 | (28%) | 132 | (24%) | 227 | (28%) |  |
| Not operated | 20 | (9%) | 103 | (19%) | 146 | (18%) |  |
| **FIGO stage** |  |  |  |  |  |  |  |
| Ic | 21 | (9%) | 47 | (9%) | 74 | (9%) |  |
| II | 18 | (8%) | 54 | (10%) | 79 | (10%) |  |
| III | 157 | (69%) | 335 | (61%) | 493 | (60%) |  |
| IV | 32 | (14%) | 113 | (21%) | 174 | (21%) |  |
| **Tumor histology** |  |  |  |  |  |  |  |
| Serous | 160 | (70%) | 363 | (66%) | 530 | (65%) |  |
| Endometrioid | 29 | (13%) | 72 | (13%) | 98 | (12%) |  |
| Clear cell | 7 | (3%) | 16 | (3%) | 27 | (3%) |  |
| Mucinous | 2 | (1%) | 15 | (3%) | 25 | (3%) | |
| Undifferentiated | 13 | (6%) | 40 | (7%) | 60 | (7%) | |
| Mixed or other | 15 | (7%) | 29 | (5%) | 44 | (5%) | |
| Missing information | 3 | (1%) | 14 | (3%) | 36 | (4%) | |
| **Grading** |  |  |  |  |  |  | |
| G1 | 7 | (3%) | 16 | (3%) | 32 | (4%) | |
| G2 | 35 | (15%) | 94 | (17%) | 137 | (17%) | |
| G3 | 156 | (68%) | 317 | (58%) | 453 | (55%) | |
| Undifferentiated | 13 | (6%) | 40 | (7%) | 60 | (7%) | |
| Missing information | 18 | (8%) | 82 | (15%) | 138 | (17%) | |
| **Treatment assigned** |  |  |  |  |  |  | |
| Carboplatin - paclitaxel | 101 | (44%) | 274 | (50%) | 410 | (50%) | |
| Carboplatin - Caelyx | 128 | (56%) | 275 | (50%) | 410 | (50%) | |

## Figure S3: Progression-free and overall survival curves in the whole study and the biomarker population

#
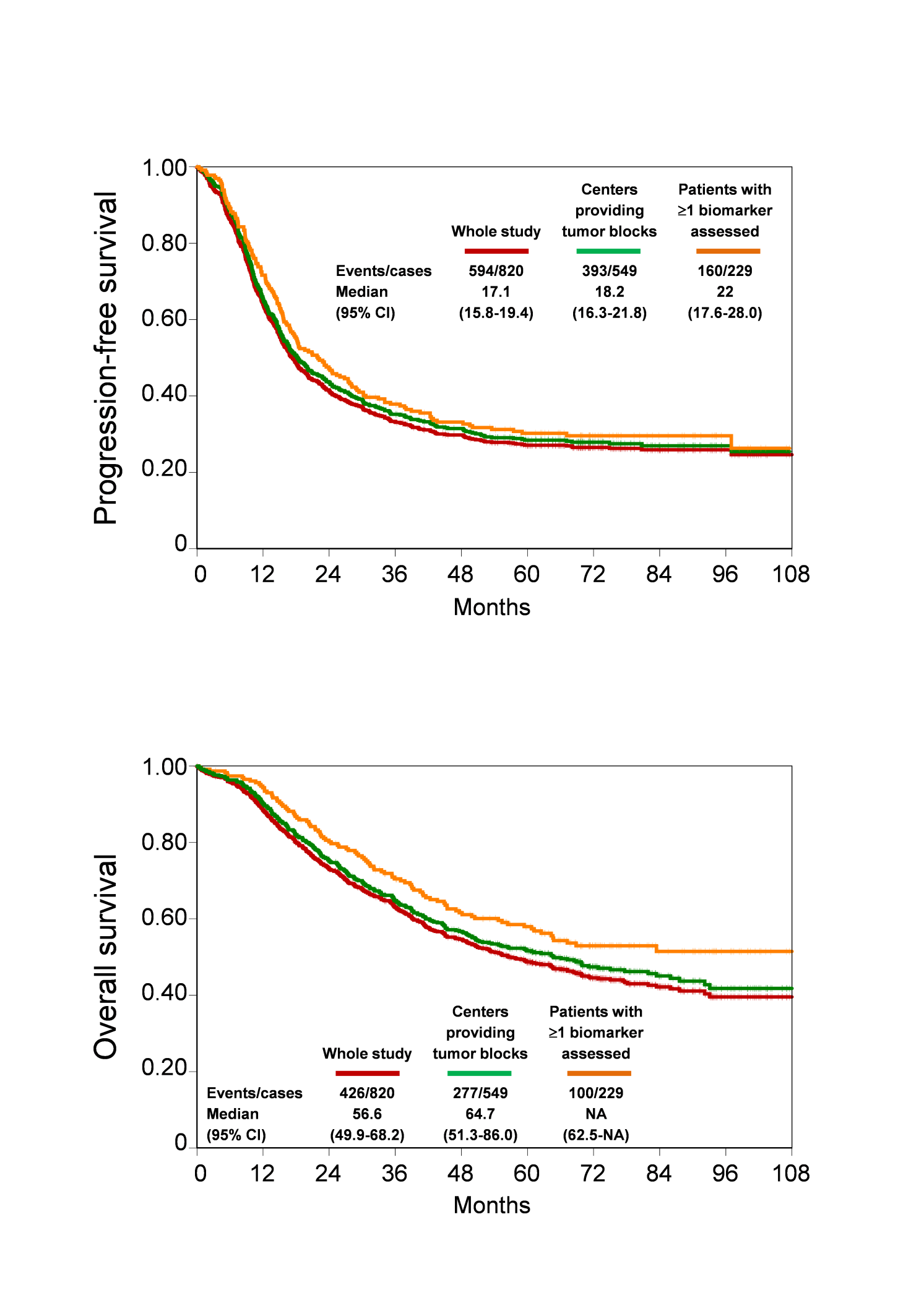


# Table S3. Distribution of biomarker reactivity by treatment arm

|  | **Carboplatin**  **Paclitaxel** | **Carboplatin**  **PLD** | **Both arms** | **P** |
| --- | --- | --- | --- | --- |
| ***Biomarkers associated with adhesion and invasion*** |  |  |  |  |
| **ALCAM** |  |  |  | 0.90 |
| Cytoplasm | 56/92 (61%) | 66/110 (60%) | 122/202 (60%) |  |
| Membrane | 36/92 (39%) | 44/110 (40%) | 80/202 (40%) |  |
| **MCAM** |  |  |  | 0.09 |
| Negative | 60/93 (65%) | 58/110 (53%) | 118/203 (58%) |  |
| Positive | 33/93 (35%) | 52/110 (47%) | 85/203 (42%) |  |
| **CAV1** |  |  |  |  |
| Tumor |  |  |  | 0.65 |
| Negative | 67/91 (74%) | 80/113 (71%) | 147/204 (72%) |  |
| Positive | 24/91 (26%) | 33/113 (29%) | 57/204 (28%) |  |
| Stroma |  |  |  | 0.73 |
| Negative | 43/93 (46%) | 50/114 (44%) | 93/207 (45%) |  |
| Moderate/positive | 50/93 (54%) | 64/114 (56%) | 114/207 (55%) |  |
| **Claudin3** |  |  |  | 0.51 |
| Low | 47/98 (48%) | 64/122 (52%) | 111/220 (50%) |  |
| High | 51/98 (52%) | 58/122 (48%) | 109/220 (50%) |  |
| ***Biomarkers associated with apoptosis*** |  |  |  |  |
| **P53** |  |  |  | 0.03 |
| Low (0%-30%) | 43/98 (44%) | 71/121 (59%) | 114/219 (52%) |  |
| High (>30%) | 55/98 (56%) | 50/121 (41%) | 105/219 (48%) |  |
| **cFLIP** |  |  |  | 0.90 |
| Negative | 57/89 (64%) | 72/111(65%) | 129/200(65%) |  |
| Positive | 32/89(36%) | 39/111(35%) | 71/200 (35%) |  |
| **TRAP** |  |  |  | 0.11 |
| Negative | 32/89 (36%) | 53/112 (47%) | 85/201 (42%) |  |
| Positive | 57/89 (64%) | 59/112 (53%) | 116/201 (58%) |  |
| **BAG3** |  |  |  | 0.87 |
| 0 -1 | 21/98 (21 %) | 23/114 (20%) | 44/212 (20%) |  |
| 2 | 22/98 (23%) | 23/114 (20%) | 45/212 (22%) |  |
| 3 | 55/98 (56%) | 68/114 (60%) | 123/212 (58%) |  |
| ***Biomarkers associated with transcription regulation*** |  |  |  |  |
| **HOX B13** |  |  |  |  |
| Cytoplasm |  |  |  | 0.30 |
| Negative | 44/96 (46%) | 65/123 (53%) | 109/219 (50%) |  |
| Positive | 52/96 (54%) | 58/123 (47%) | 110/219 (50%) |  |
| Nucleus |  |  |  | 0.53 |
| Negative | 49/96 (51%) | 68/123 (55%) | 117/219 (53%) |  |
| Positive | 47/96 (49%) | 55/123 (45%) | 102/219 (47%) |  |
| **HMGA2** |  |  |  | 0.19 |
| 0 - 1 | 78/100 (78%) | 85/121(70%) | 163/221(74%) |  |
| 2 - 3 | 22/100(22%) | 36/121(30%) | 58/221(26%) |  |
| **CDK6** |  |  |  |  |
| Intensity |  |  |  | 0.76 |
| Low/moderate | 22/99 (22%) | 25/124 (20%) | 47/223 (21%) |  |
| High | 48/99 (49%) | 57/124 (46%) | 105/223 (47%) |  |
| Very high | 29/99 (29%) | 42/124 (34%) | 71/223 (32%) |  |
| Localization |  |  |  | 0.95 |
| Cytoplasm | 67/99 (68%) | 79/124 (64%) | 156/223 (70%) |  |
| Cytoplasm/membrane | 24/99 (24%) | 27/124 (22%) | 51/223 (23%) |  |
| Cytoplasm/nucleus | 8/99 (8%) | 8/124 (6%) | 16/223 (7%) |  |

| **Table S3. Distribution of biomarker reactivity by treatment arm (continued)** | | | | |
| --- | --- | --- | --- | --- |
|  | **Carboplatin**  **Paclitaxel** | **Carboplatin**  **PLD** | **Both arms** | **P** |
| ***Biomarkers associated with metabolism*** |  |  |  |  |
| **Leptin receptor** |  |  |  | 0.42 |
| <10% | 30/97 (31%) | 48/122 (39%) | 78/219 (36%) |  |
| 10-50% | 16/97 (16%) | 19/122 (16%) | 35/219 (16%) |  |
| >50% | 51/97 (53%) | 55/122 (45%) | 106/219 (48%) |  |
| **pAMPK** |  |  |  | 0.52 |
| Negative | 30/66 (45%) | 35/87 (40%) | 65/153 (42%) |  |
| Positive | 36/66 (55%) | 52/87 (60%) | 88/153 (58%) |  |
| **pACC** |  |  |  | 0.51 |
| Negative | 37/76 (49%) | 41/94 (44%) | 78/170 (46%) |  |
| Positive | 39/76 (51%) | 53/94 (56%) | 92/170 (54%) |  |
| ***Biomarkers associated with DNA repair*** |  |  |  |  |
| **Stathmin** |  |  |  | 0.16 |
| Negative/moderate intensity | 72/96 (75%) | 78/118 (66%) | 150/214 (70%) |  |
| High intensity | 24/96 (25%) | 40/118 (34%) | 64/214 (30%) |  |
| **DNA-PK** |  |  |  | 0.28 |
| Negative/moderate intensity | 55/97 (57%) | 76/119 (66%) | 131/216 (60%) |  |
| High intensity | 42/97 (43%) | 43/119 (36%) | 85/216 (40%) |  |

# Table S4 - Pairwise associations between biomarkers

|  | **ALCAM** | **MCAM** | **CAV1 tumor** | **CAV1 stroma** | **Claudin3** | **p53** | **cFLIP** | **TRAP1** | **HMGA2** | **BAG3** | **HOXB13 cytoplasm** | **HOXB13 nucleus** | **CDK6 intensity** | **CDK6 localization** | **pAMPK** | **Leptin receptor** | **Stathmin** | **DNA-PK** | **pACC** |
| --- | --- | --- | --- | --- | --- | --- | --- | --- | --- | --- | --- | --- | --- | --- | --- | --- | --- | --- | --- |
| **ALCAM** | 1.000 | 0.079 | 0.134 | 0.099 | 0.016 | 0.033 | 0.051 | 0.139 | 0.106 | 0.100 | 0.055 | 0.142 | 0.186 | 0.170 | 0.162 | 0.168 | **0.320** | 0.159 | 0.016 |
| **MCAM** | 0.275 | 1.000 | 0.029 | 0.109 | 0.012 | 0.028 | 0.036 | 0.036 | 0.068 | 0.071 | 0.155 | 0.047 | 0.049 | 0.085 | 0.027 | 0.095 | 0.074 | 0.103 | 0.016 |
| **CAV1 tumor** | 0.067 | 0.684 | 1.000 | **0.276** | 0.165 | 0.071 | 0.084 | 0.110 | 0.093 | 0.065 | 0.153 | 0.026 | **0.301** | 0.156 | 0.034 | 0.122 | 0.080 | 0.185 | 0.111 |
| **CAV1 stroma** | 0.395 | 0.316 | **0.000** | 1.000 | 0.090 | 0.076 | 0.145 | 0.211 | 0.103 | 0.098 | 0.226 | 0.232 | 0.119 | 0.068 | 0.202 | 0.065 | 0.150 | 0.205 | 0.271 |
| **Claudin3** | 0.817 | 0.868 | 0.019 | 0.437 | 1.000 | 0.061 | 0.124 | 0.181 | 0.024 | 0.106 | 0.187 | 0.112 | 0.052 | 0.110 | 0.069 | 0.090 | 0.108 | 0.082 | 0.146 |
| **p53** | 0.645 | 0.695 | 0.314 | 0.558 | 0.374 | 1.000 | 0.122 | 0.026 | 0.099 | 0.078 | 0.104 | 0.159 | 0.083 | 0.097 | 0.140 | 0.076 | 0.162 | 0.183 | 0.124 |
| **cFLIP** | 0.488 | 0.624 | 0.249 | 0.134 | 0.081 | 0.086 | 1.000 | 0.169 | 0.108 | 0.132 | 0.029 | 0.117 | 0.204 | 0.038 | 0.082 | 0.030 | 0.177 | 0.044 | 0.122 |
| **TRAP1** | 0.059 | 0.624 | 0.137 | 0.016 | 0.011 | 0.713 | 0.023 | 1.000 | 0.018 | 0.050 | 0.131 | 0.118 | 0.232 | 0.067 | 0.154 | 0.088 | 0.085 | 0.246 | 0.219 |
| **HMGA2** | 0.135 | 0.333 | 0.185 | 0.335 | 0.726 | 0.146 | 0.131 | 0.801 | 1.000 | 0.055 | 0.027 | 0.054 | 0.110 | **0.266** | 0.104 | 0.166 | 0.175 | 0.072 | 0.227 |
| **BAG3** | 0.379 | 0.610 | 0.661 | 0.418 | 0.309 | 0.530 | 0.185 | 0.789 | 0.728 | 1.000 | 0.041 | 0.058 | 0.181 | 0.129 | 0.214 | **0.210** | 0.062 | 0.089 | **0.286** |
| **HOXB13 cytoplasm** | 0.445 | 0.029 | 0.031 | 0.006 | 0.006 | 0.131 | 0.686 | 0.067 | 0.693 | 0.841 | 1.000 | **0.289** | **0.248** | 0.201 | 0.072 | 0.210 | 0.142 | 0.224 | 0.201 |
| **HOXB13 nucleus** | 0.047 | 0.514 | 0.711 | 0.004 | 0.102 | 0.021 | 0.100 | 0.098 | 0.428 | 0.708 | **0.000** | 1.000 | 0.148 | 0.095 | 0.158 | 0.054 | 0.127 | 0.173 | 0.159 |
| **CDK6 intensity** | 0.031 | 0.786 | **0.000** | 0.215 | 0.742 | 0.471 | 0.016 | 0.005 | 0.267 | 0.008 | **0.001** | 0.095 | 1.000 | 0.169 | 0.129 | 0.120 | 0.125 | 0.080 | 0.139 |
| **CDK6 localization** | 0.056 | 0.484 | 0.084 | 0.750 | 0.265 | 0.358 | 0.864 | 0.639 | **0.000** | 0.135 | 0.013 | 0.381 | 0.012 | 1.000 | 0.089 | 0.098 | 0.171 | 0.052 | 0.115 |
| **pAMPK** | 0.051 | 0.752 | 0.685 | 0.050 | 0.396 | 0.084 | 0.326 | 0.070 | 0.204 | 0.033 | 0.381 | 0.055 | 0.280 | 0.549 | 1.000 | 0.234 | **0.313** | **0.388** | **0.433** |
| **Leptin receptor** | 0.060 | 0.407 | 0.222 | 0.785 | 0.418 | 0.540 | 0.914 | 0.469 | 0.052 | **0.001** | 0.009 | 0.734 | 0.183 | 0.382 | 0.016 | 1.000 | 0.062 | 0.168 | 0.215 |
| **Stathmin** | **0.000** | 0.583 | 0.536 | 0.062 | 0.291 | 0.065 | 0.046 | 0.497 | 0.038 | 0.816 | 0.124 | 0.186 | 0.157 | 0.014 | **0.001** | 0.805 | 1.000 | **0.331** | 0.122 |
| **DNA-PK** | 0.082 | 0.350 | 0.034 | 0.002 | 0.491 | 0.030 | 0.830 | 0.003 | 0.574 | 0.521 | 0.005 | 0.042 | 0.599 | 0.886 | **0.000** | 0.018 | **0.000** | 1.000 | **0.487** |
| **pACC** | 0.841 | 0.842 | 0.165 | 0.003 | 0.060 | 0.111 | 0.131 | 0.006 | 0.003 | **0.001** | 0.010 | 0.043 | 0.197 | 0.328 | **0.000** | 0.021 | 0.300 | **0.000** | 1.000 |

Values above the diagonal represent Cramer's V to measure pairwise associations. Values under the diagonal represent the corresponding p-values (in bold p-values ≤0.001).

# ALCAM

## Table S5.Characteristics of patients according to ALCAM category

|  | **ALCAM cytoplasm**  **(n=122)** | | **ALCAM membrane**  **(n=80)** | | **P value** |
| --- | --- | --- | --- | --- | --- |
| **Median age** (range) | 59 | (28-77) | 54 | (34-75) | 0.009 |
| Age < 70 yrs | 106 | (87%) | 72 | (90%) | 0.50 |
| Age > 70 yrs | 16 | (13%) | 8 | (10%) |  |
| **ECOG performance status** |  |  |  |  | 0.75 |
| 0-1 | 118 | (97%) | 78 | (98%) |  |
| 2 | 4 | (3%) | 2 | (2%) |  |
| **Residual disease** |  |  |  |  | 0.11 |
| None | 40 | (33%) | 39 | (49%) |  |
| ≤ 1 cm | 30 | (25%) | 18 | (22%) |  |
| > 1 cm | 41 | (34%) | 19 | (24%) |  |
| Not operated | 11 | (9%) | 4 | (5%) |  |
| **FIGO stage** |  |  |  |  | 0.09 |
| Ic | 10 | (8%) | 7 | (8%) |  |
| II | 4 | (3%) | 10 | (12%) |  |
| III | 89 | (73%) | 53 | (66%) |  |
| IV | 19 | (16%) | 10 | (12%) |  |
| **Tumor histology** |  |  |  |  | 0.44 |
| Serous | 88 | (72%) | 55 | (69%) |  |
| Endometrioid | 14 | (11%) | 10 | (12%) |  |
| Clear cell | 4 | (3%) | 2 | (3%) |  |
| Mucinous | 1 | (1%) | 1 | (1%) |  |
| Undifferentiated | 8 | (7%) | 3 | (4%) |  |
| Mixed or other | 7 | (6%) | 6 | (8%) |  |
| Missing information | - | - | 3 | (4%) |  |
| **Grading** |  |  |  |  | 0.45 |
| G1 | 6 | (5%) | 1 | (1%) |  |
| G2 | 18 | (15%) | 9 | (11%) |  |
| G3 | 87 | (71%) | 62 | (78%) |  |
| G4 | 8 | (7%) | 4 | (5%) |  |
| Missing information | 3 | (2%) | 4 | (5%) |  |

## Figure S4. Progression-free survival


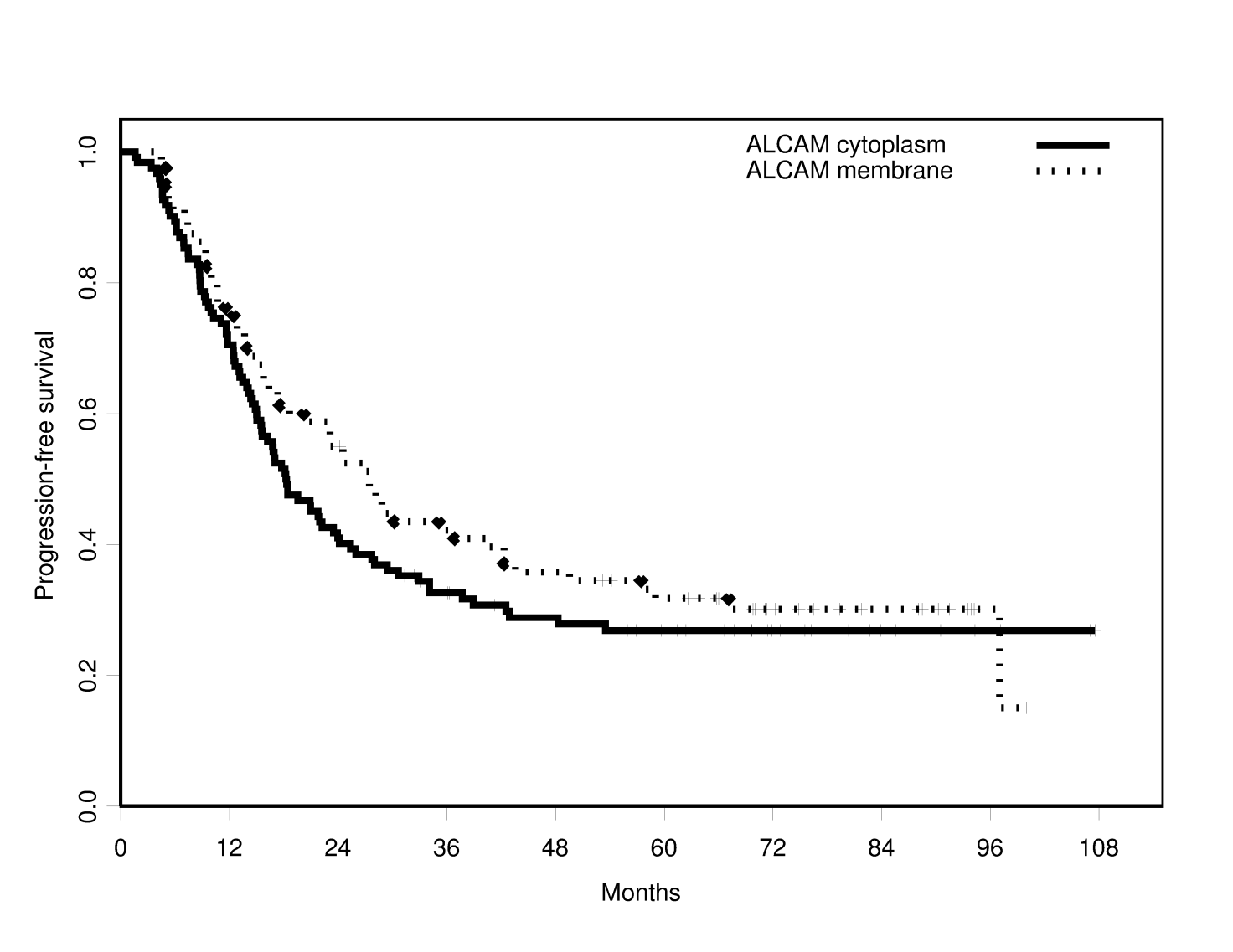


| **ALCAM expression** | **N. pts** | **Events** | **Median PFS (months)** | **95%CI** |
| --- | --- | --- | --- | --- |
| Cytoplasm | 122 | 88 | 18.2 | 15.6 - 24.1 |
| Membrane | 80 | 56 | 27.4 | 20.2 - 42.3 |

## Figure S5. Overall survival

**
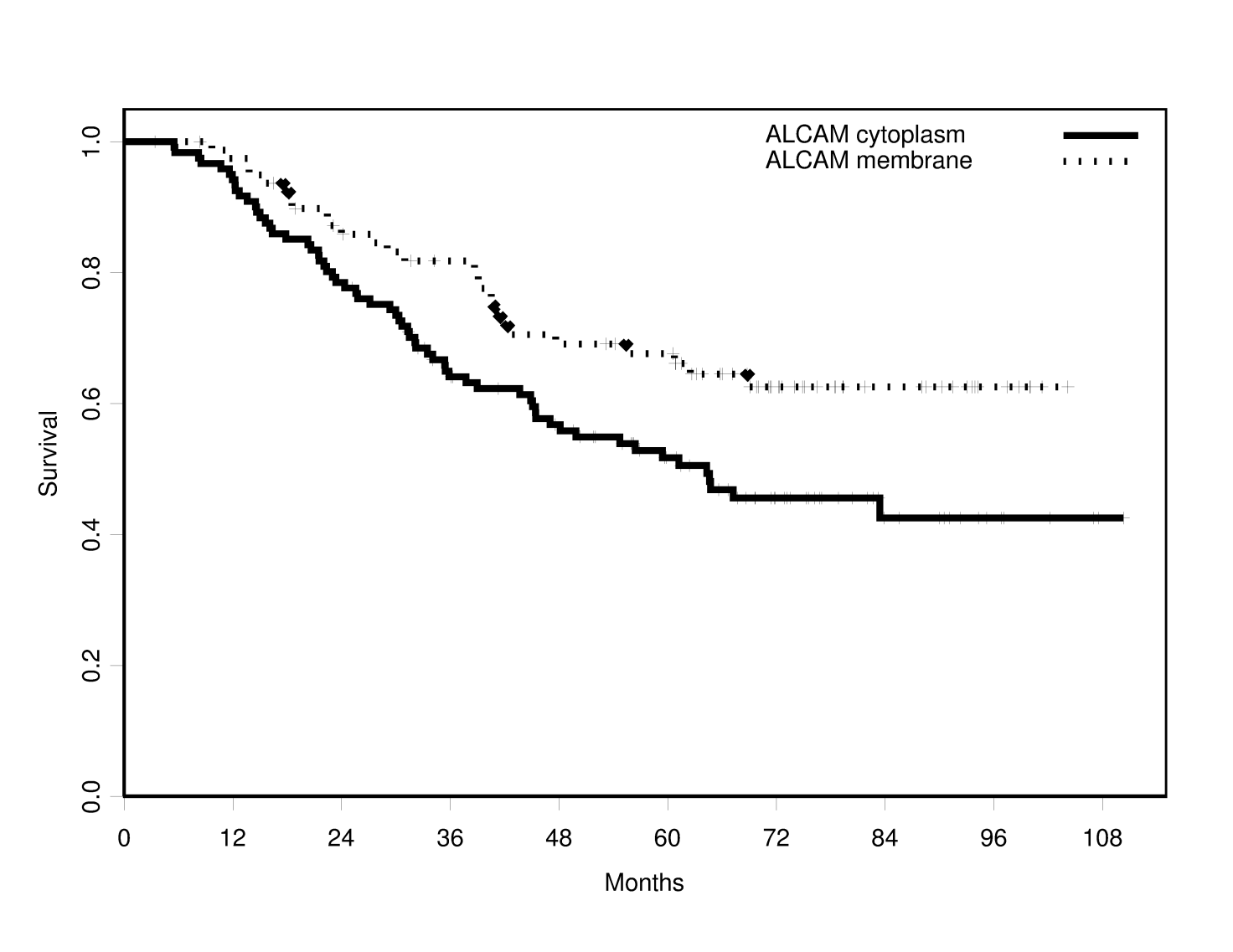
**

| **ALCAM expression** | **N. pts** | **Events** | **Median OS (months)** | **95%CI** |
| --- | --- | --- | --- | --- |
| Cytoplasm | 122 | 62 | 64.3 | 45.4 - n.a. |
| Membrane | 80 | 27 | n.a. | n.a. - n.a. |

# MCAM

## Table S6. Characteristics of patients according to MCAM category

|  | **MCAM negative**  **(n=118)** | | **MCAM positive**  **(n=85)** | | **P value** |
| --- | --- | --- | --- | --- | --- |
| **Median age** (range) | 56 | (28 - 77) | 58 | (30 - 74) | 0.20 |
| Age < 70 yrs | 104 | (88%) | 73 | (86%) | 0.64 |
| Age > 70 yrs | 14 | (12%) | 12 | (14%) |  |
| **ECOG performance status** |  |  |  |  | 0.20 |
| 0-1 | 113 | (96%) | 84 | (99%) |  |
| 2 | 5 | (4%) | 1 | (1%) |  |
| **Residual disease** |  |  |  |  | 0.08 |
| None | 56 | (47%) | 26 | (31%) |  |
| ≤ 1 cm | 22 | (19%) | 23 | (27%) |  |
| > 1 cm | 34 | (29%) | 28 | (33%) |  |
| Not operated | 6 | (5%) | 8 | (9%) |  |
| **FIGO stage** |  |  |  |  | 0.02 |
| Ic | 13 | (11%) | 3 | (4%) |  |
| II | 12 | (10%) | 2 | (2%) |  |
| III | 76 | (64%) | 67 | (79%) |  |
| IV | 17 | (14%) | 13 | (15%) |  |
| **Tumor histology** |  |  |  |  | 0.19 |
| Serous | 75 | (64%) | 69 | (81%) |  |
| Endometrioid | 18 | (15%) | 7 | (8%) |  |
| Clear cell | 5 | (4%) | 1 | (1%) |  |
| Mucinous | 2 | (2%) | 0 | - |  |
| Undifferentiated | 7 | (6%) | 4 | (5%) |  |
| Mixed or other | 9 | (8%) | 3 | (4%) |  |
| Missing information | 2 | (2%) | 1 | (1%) |  |
| **Grading** |  |  |  |  | 0.73 |
| G1 | 3 | (2.5%) | 4 | (5%) |  |
| G2 | 16 | (14%) | 11 | (13%) |  |
| G3 | 87 | (74%) | 62 | (73%) |  |
| G4 | 9 | (8%) | 4 | (5%) |  |
| Missing information | 3 | (2.5%) | 4 | (5%) |  |

## Figure S6. Progression-free survival

**
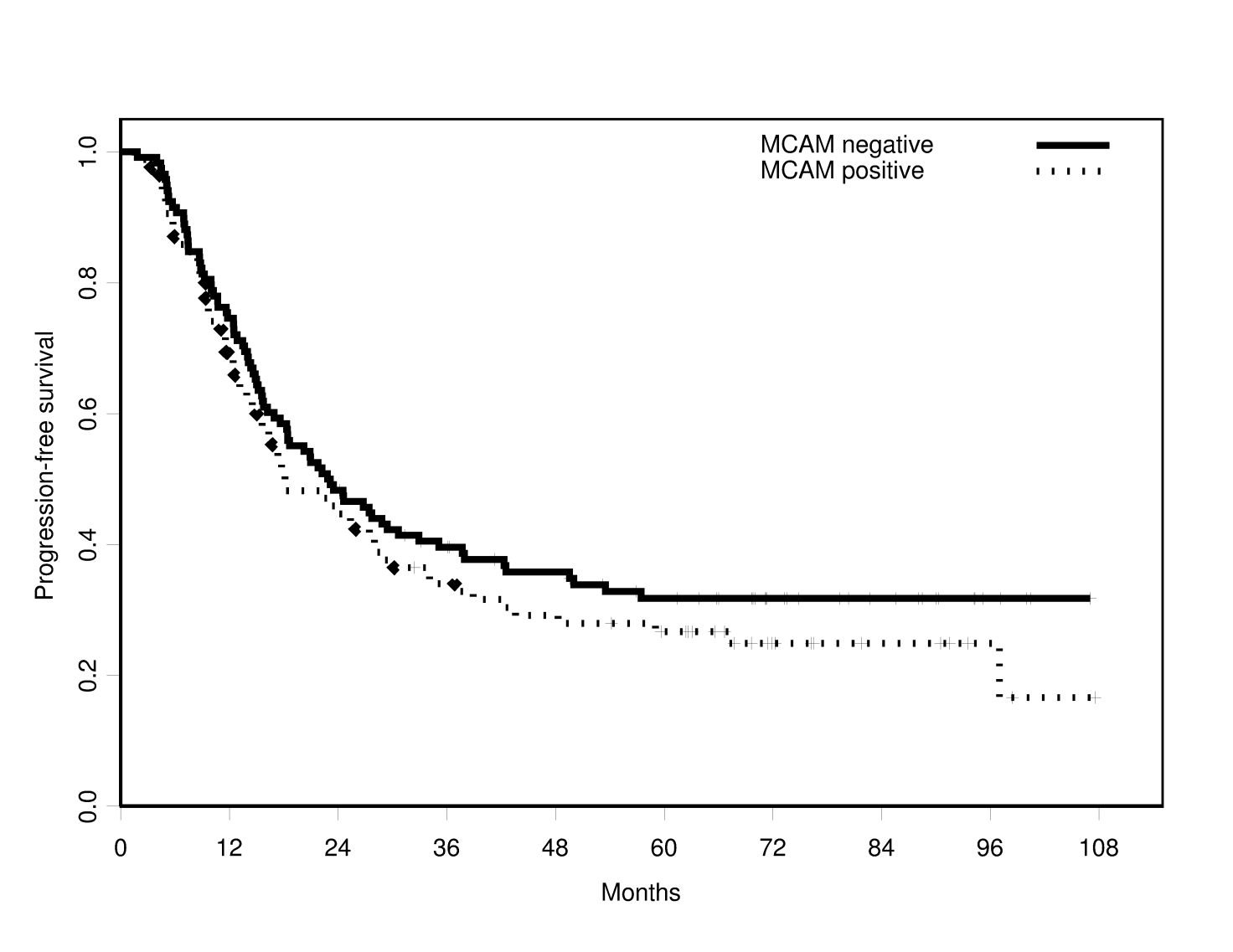
**

| **MCAM expression** | **N. pts** | **Events** | **Median PFS (months)** | **95%CI** |
| --- | --- | --- | --- | --- |
| Negative | 118 | 79 | 23.1 | 18.3 - 35.1 |
| Positive | 85 | 64 | 18.2 | 15.0 - 29.0 |

## Figure S7. Overall survival

**
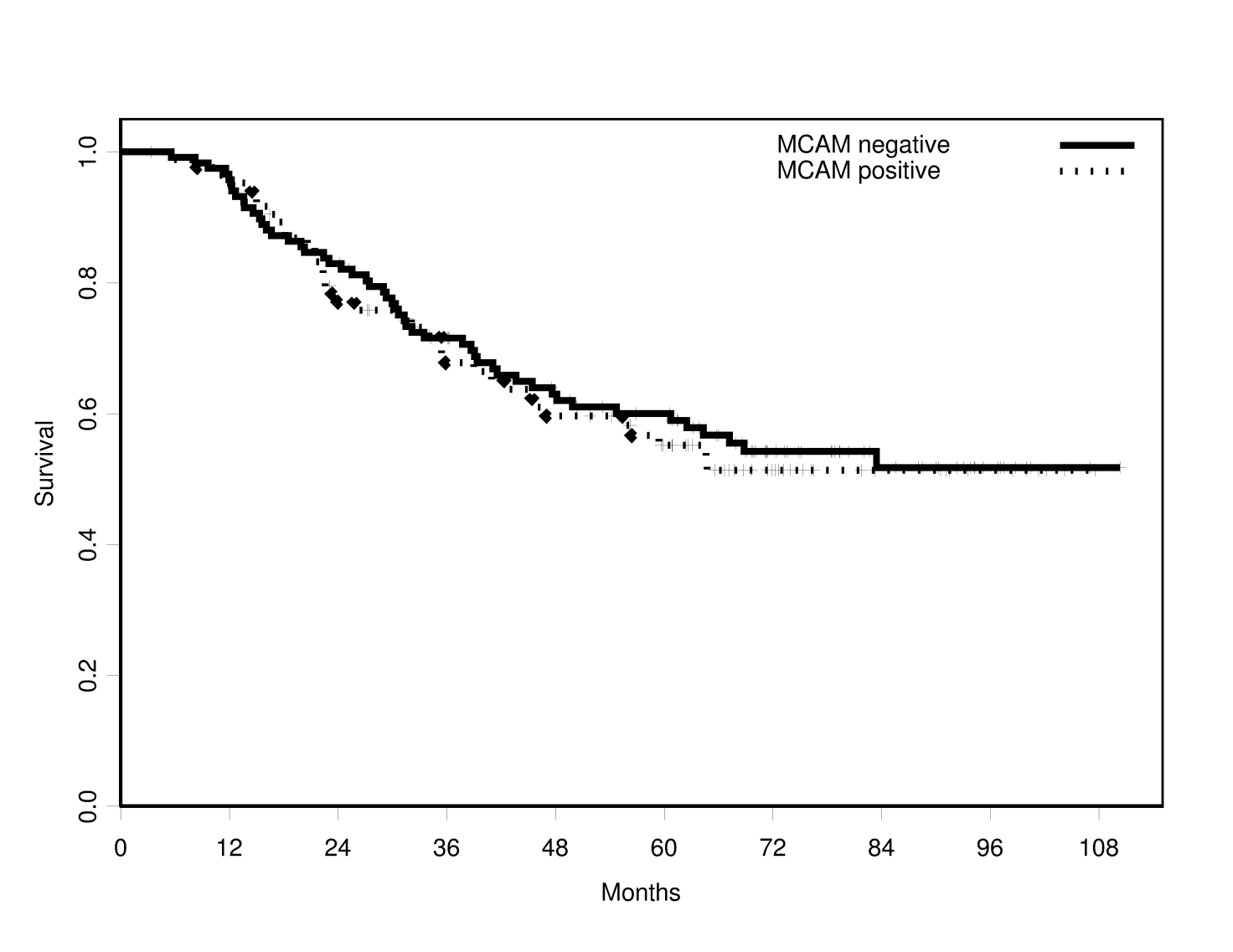
**

| **MCAM expression** | **N. pts** | **Events** | **Median OS (months)** | **95%CI** |
| --- | --- | --- | --- | --- |
| Negative | 118 | 51 | n.a. | 62.5 - n.a. |
| Positive | 85 | 37 | n.a. | 47.0 - n.a. |

# CAV1 (Tumor)

## Table S7. Characteristics of patients according to CAV1 (tumor) category

|  | **CAV1 tumor negative**  **(n=147)** | | **CAV1 tumor positive**  **(n=57)** | | **P value** |
| --- | --- | --- | --- | --- | --- |
| **Median age** (range) | 57 | (28-77) | 56 | (32-76) | 0.77 |
| Age < 70 yrs | 129 | (88%) | 50 | (88%) | 0.99 |
| Age > 70 yrs | 18 | (12%) | 7 | (12%) |  |
| **ECOG performance status** |  |  |  |  | 0.22 |
| 0-1 | 144 | (98%) | 54 | (95%) |  |
| 2 | 3 | (2%) | 3 | (5%) |  |
| **Residual disease** |  |  |  |  | 0.48 |
| None | 61 | (41%) | 24 | (42%) |  |
| ≤ 1 cm | 36 | (24%) | 9 | (16%) |  |
| > 1 cm | 39 | (27%) | 20 | (35%) |  |
| Not operated | 11 | (8%) | 4 | (7%) |  |
| **FIGO stage** |  |  |  |  | 0.33 |
| Ic | 13 | (9%) | 3 | (5%) |  |
| II | 15 | (10%) | 2 | (4%) |  |
| III | 100 | (68%) | 43 | (75%) |  |
| IV | 19 | (13%) | 9 | (16%) |  |
| **Tumor histology** |  |  |  |  | 0.24 |
| Serous | 101 | (69%) | 44 | (77%) |  |
| Endometrioid | 21 | (14%) | 4 | (7%) |  |
| Clear cell | 5 | (3%) | 1 | (2%) |  |
| Mucinous | 2 | (1%) | - | - |  |
| Undifferentiated | 10 | (7%) | 1 | (2%) |  |
| Mixed or other | 7 | (5%) | 6 | (11%) |  |
| Missing information | 1 | (1%) | 1 | (2%) |  |
| **Grading** |  |  |  |  | 0.78 |
| G1 | 4 | (3%) | 1 | (2%) |  |
| G2 | 24 | (16%) | 7 | (12%) |  |
| G3 | 106 | (72%) | 43 | (75%) |  |
| G4 | 8 | (5%) | 5 | (9%) |  |
| Missing information | 5 | (3%) | 1 | (2%) |  |

## Figure S8. Progression-free survival

**
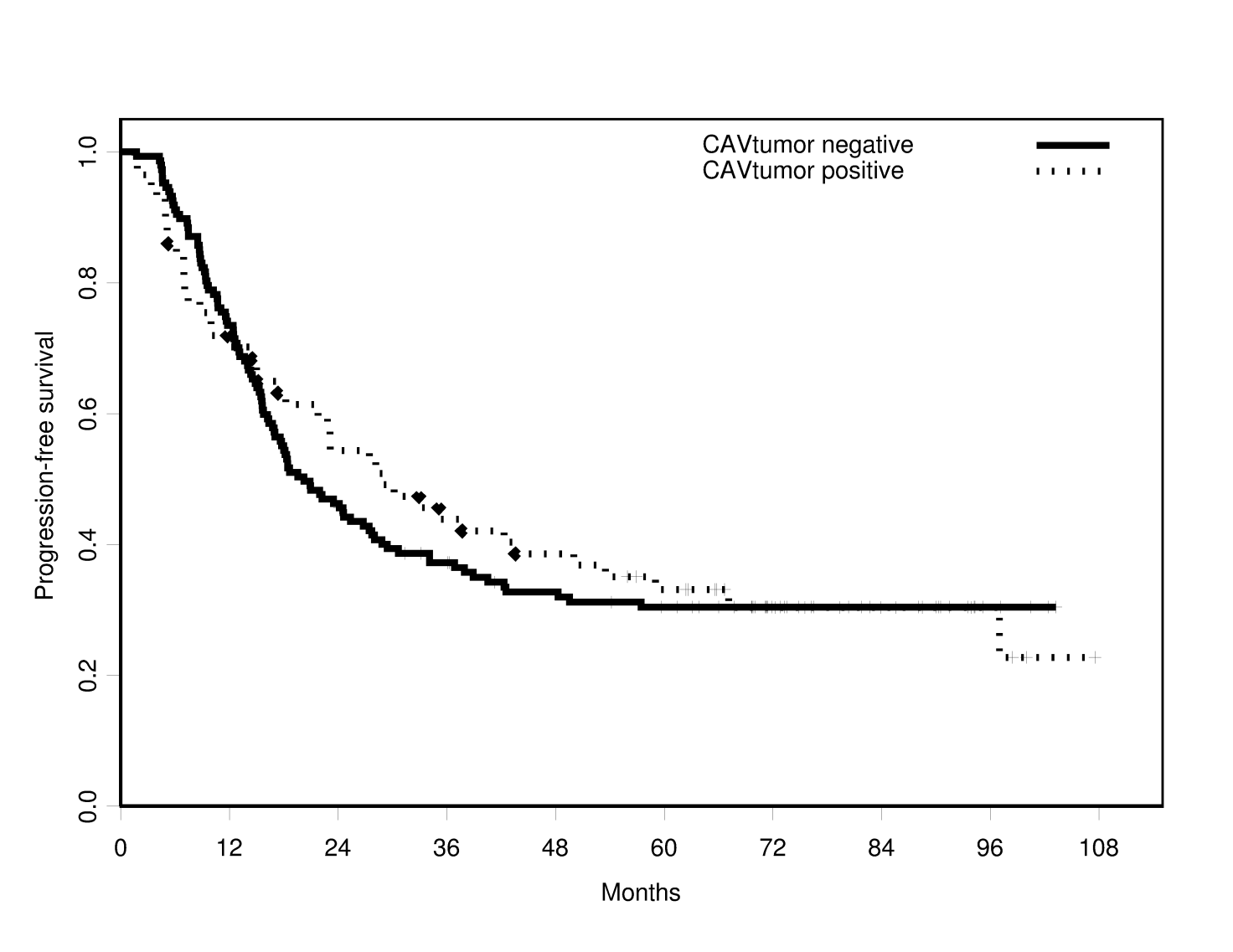
**

| **CAV1 tumor expression** | **N. pts** | **Events** | **Median PFS (months)** | **95%CI** |
| --- | --- | --- | --- | --- |
| Negative | 147 | 101 | 20.2 | 16.9 - 28.0 |
| Positive | 57 | 40 | 29.0 | 18.2 - 53.5 |

## Figure S9. Overall survival

**
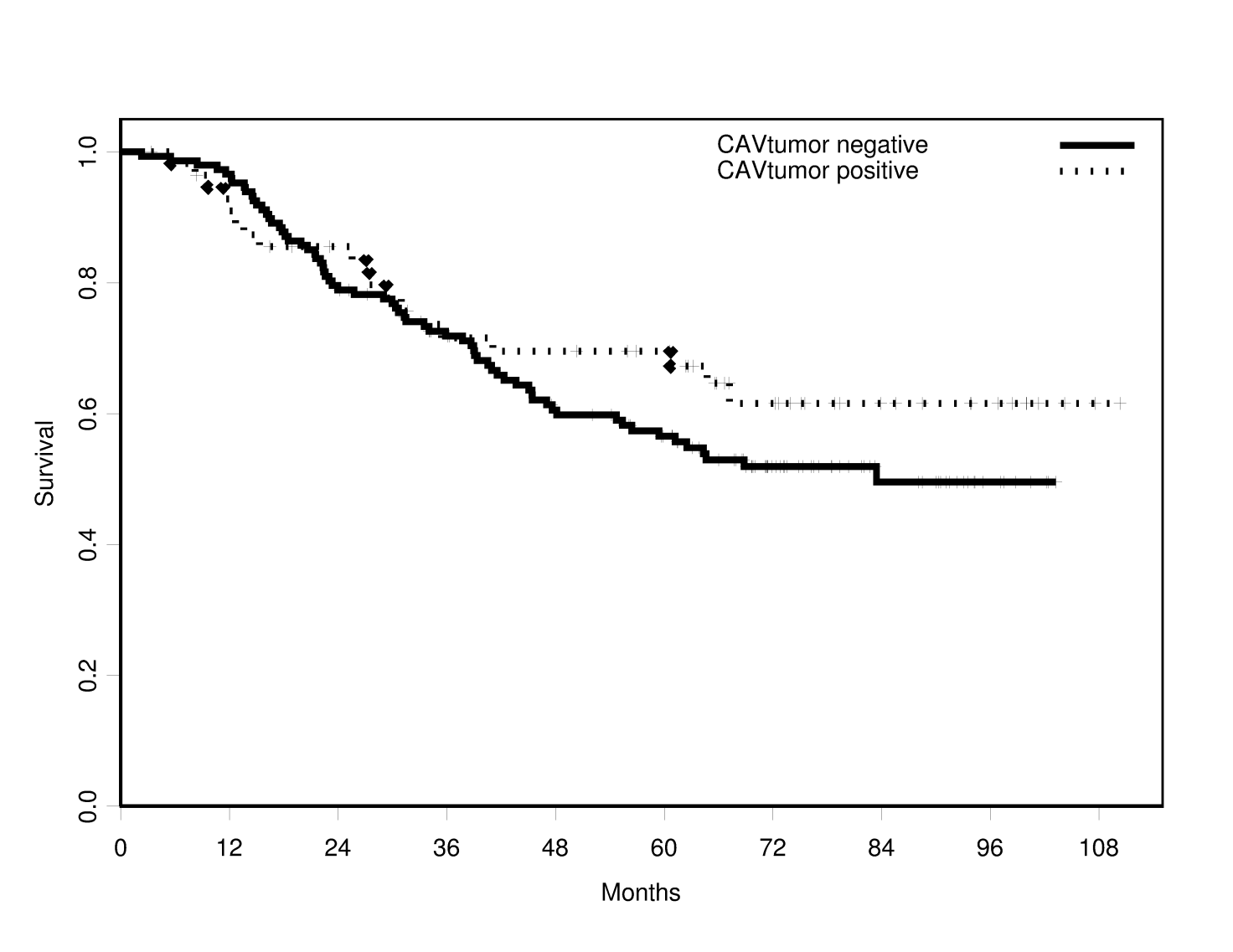
**

| **CAV1 tumor expression** | **N. pts** | **Events** | **Median OS (months)** | **95%CI** |
| --- | --- | --- | --- | --- |
| Negative | 147 | 67 | 83.4 | 56.4 - n.a. |
| Positive | 57 | 19 | n.a. | 67.2 - n.a. |

# CAV1 (Stroma)

## Table S8. Characteristics of patients according to CAV1 (stroma) category

|  | **CAV1 stroma negative**  **(n=93)** | | **CAV1 stroma moderate / positive**  **(n=114)** | | **P value** | |
| --- | --- | --- | --- | --- | --- | --- |
| **Median age** (range) | 57 | (28 - 77) | 57 | (28 - 76) | | 0.13 |
| Age < 70 yrs | 78 | (84%) | 104 | (91%) | | 0.11 |
| Age > 70 yrs | 15 | (16%) | 10 | (9%) | |  |
| **ECOG performance status** |  |  |  |  | | 0.80 |
| 0-1 | 90 | (97%) | 111 | (97%) | |  |
| 2 | 3 | (3%) | 3 | (3%) | |  |
| **Residual disease** |  |  |  |  | | 0.01 |
| None | 31 | (33%) | 56 | (49%) | |  |
| ≤ 1 cm | 22 | (24%) | 24 | (21%) | |  |
| > 1 cm | 28 | (30%) | 31 | (27%) | |  |
| Not operated | 12 | (13%) | 3 | (3%) | |  |
| **FIGO stage** |  |  |  |  | | 0.39 |
| Ic | 7 | (8%) | 10 | (9%) | |  |
| II | 8 | (9%) | 10 | (9%) | |  |
| III | 69 | (74%) | 74 | (65%) | |  |
| IV | 9 | (10%) | 20 | (18%) | |  |
| **Tumor histology** |  |  |  |  | | 0.17 |
| Serous | 67 | (72%) | 80 | (70%) | |  |
| Endometrioid | 13 | (14%) | 13 | (11%) | |  |
| Clear cell | 3 | (3%) | 3 | (3%) | |  |
| Mucinous | 2 | (2%) | - | - | |  |
| Undifferentiated | 3 | (3%) | 8 | (7%) | |  |
| Mixed or other | 3 | (3%) | 10 | (9%) | |  |
| Missing information | 2 | (2%) | - | - | |  |
| **Grading** |  |  |  |  | | 0.82 |
| G1 | 3 | (3%) | 3 | (3%) | |  |
| G2 | 15 | (16%) | 17 | (15%) | |  |
| G3 | 66 | (71%) | 84 | (74%) | |  |
| G4 | 5 | (5%) | 8 | (7%) | |  |
| Missing information | 4 | (4%) | 2 | (2%) | |  |

## Figure S10. Progression-free survival

**
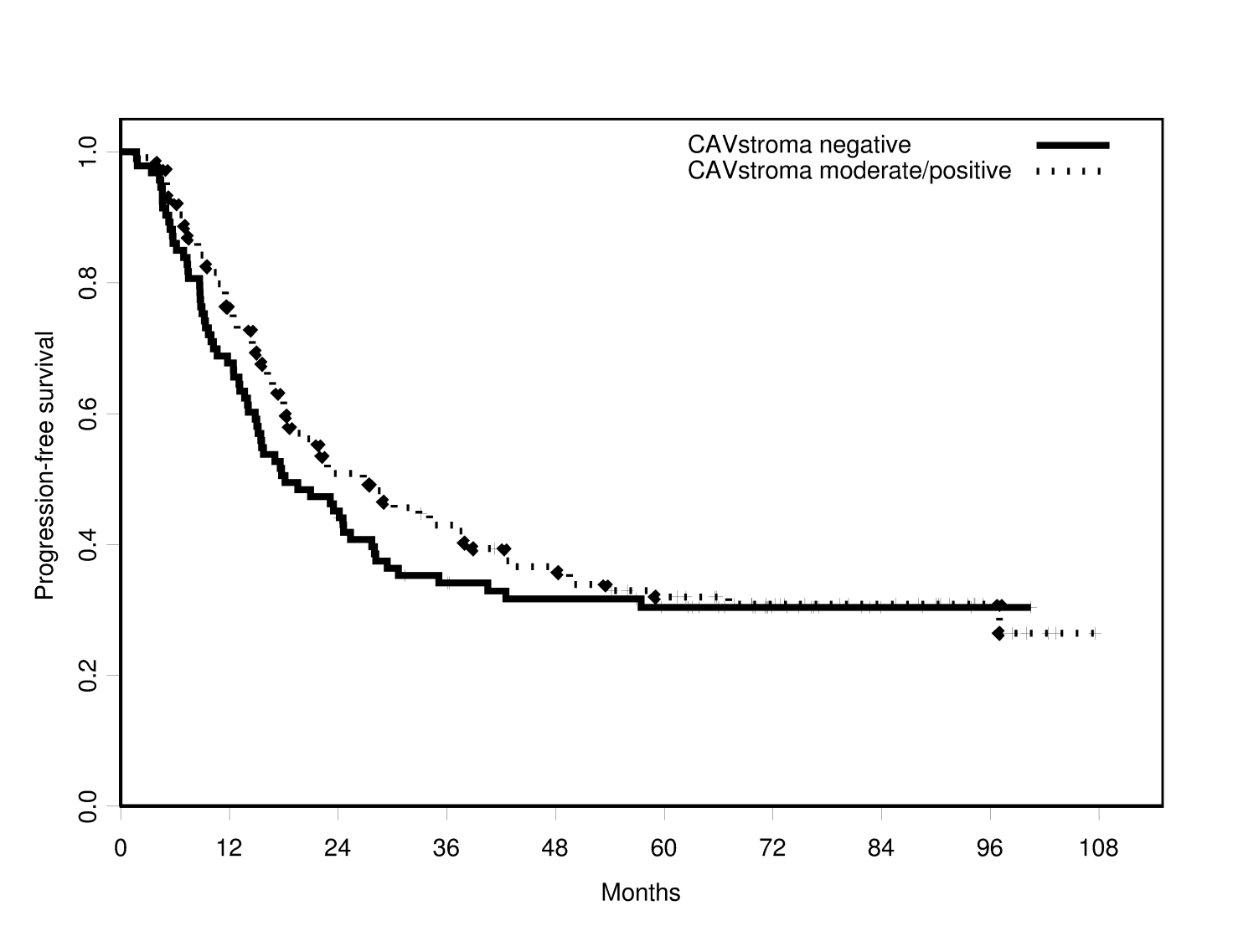
**

| **CAV1 stroma**  **expression** | **N. pts** | **Events** | **Median PFS (months)** | **95%CI** |
| --- | --- | --- | --- | --- |
| Negative | 93 | 64 | 18.2 | 14.9 - 28.0 |
| Moderate/positive | 114 | 79 | 27.1 | 18.5 - 38.9 |

## Figure S11. Overall survival

**
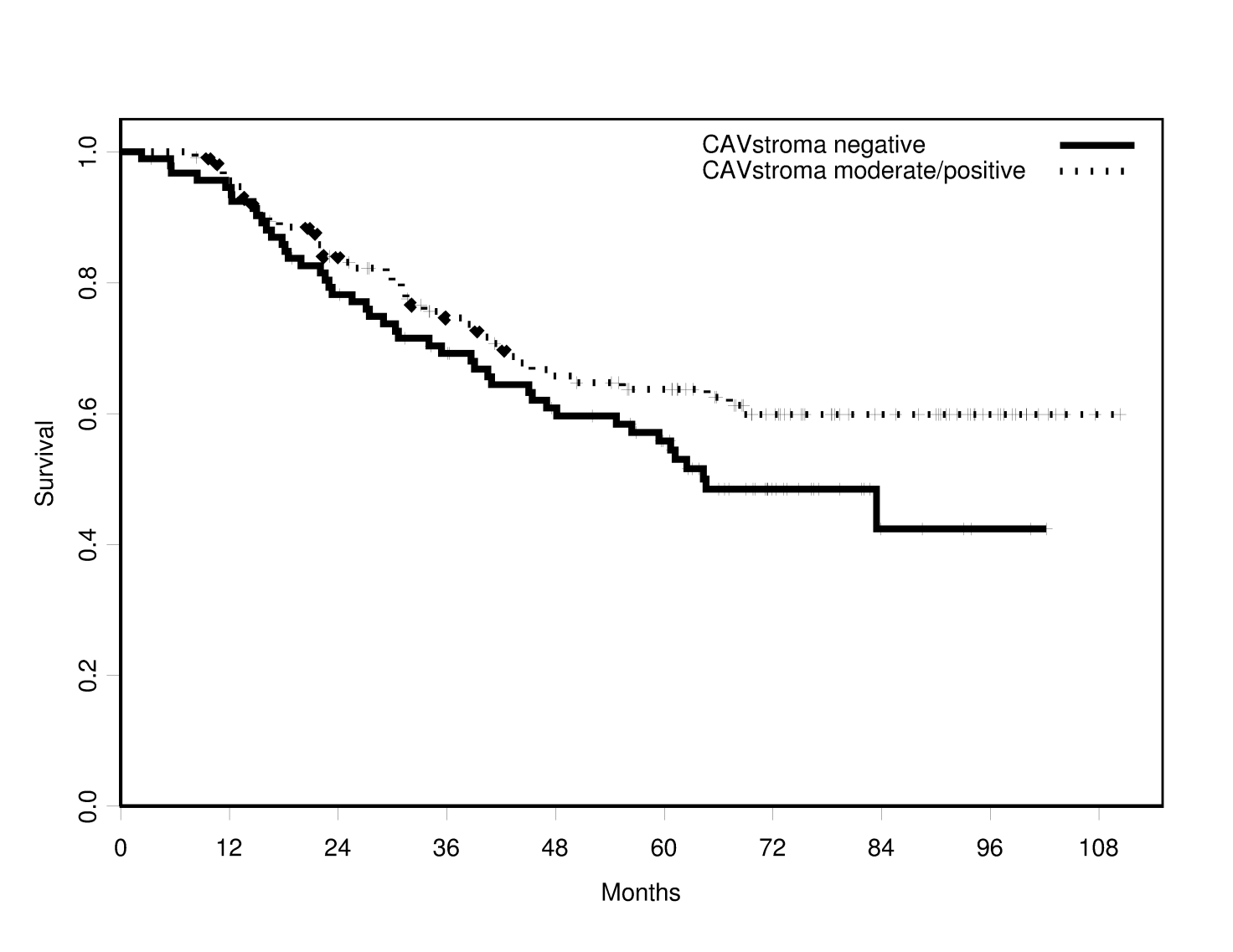
**

| **CAV1 stroma**  **expression** | **N. pts** | **Events** | **Median OS (months)** | **95%CI** |
| --- | --- | --- | --- | --- |
| Negative | 93 | 45 | 64.6 | 54.7 - n.a. |
| Moderate / positive | 114 | 42 | n.a. | n.a. - n.a. |

# Claudin3

## Table S9. Characteristics of patients according to Claudin3 category

|  | **Claudin3 low**  **(negative-weak)**  **(n=111)** | | **Claudin3 high**  **(moderate-strong)**  **(n=109)** | | **P value** |
| --- | --- | --- | --- | --- | --- |
| **Median age** (range) | 58 | (28-77) | 57 | (37-77) | 0.94 |
| Age < 70 yrs | 100 | (90%) | 93 | (85%) | 0.28 |
| Age > 70 yrs | 11 | (10%) | 16 | (15%) |  |
| **ECOG performance status** |  |  |  |  | 0.24 |
| 0-1 | 109 | (98%) | 104 | (95%) |  |
| 2 | 2 | (2%) | 5 | (5%) |  |
| **Residual disease** |  |  |  |  | 0.25 |
| None | 53 | (48%) | 39 | (36%) |  |
| ≤ 1 cm | 21 | (19%) | 27 | (25%) |  |
| > 1 cm | 27 | (24%) | 35 | (32%) |  |
| Not operated | 10 | (9%) | 8 | (7%) |  |
| **FIGO stage** |  |  |  |  | 0.01 |
| Ic | 14 | (13%) | 6 | (6%) |  |
| II | 13 | (12%) | 5 | (5%) |  |
| III | 66 | (59%) | 86 | (79%) |  |
| IV | 18 | (16%) | 12 | (11%) |  |
| **Tumor histology** |  |  |  |  | 0.63 |
| Serous | 79 | (71%) | 76 | (70%) |  |
| Endometrioid | 12 | (11%) | 15 | (14%) |  |
| Clear cell | 2 | (2%) | 4 | (4%) |  |
| Mucinous | 2 | (2%) | 0 | (0%) |  |
| Undifferentiated | 7 | (6%) | 5 | (5%) |  |
| Mixed or other | 7 | (6%) | 8 | (7%) |  |
| Missing info | 2 | (2%) | 1 | (1%) |  |
| **Grading** |  |  |  |  | 0.21 |
| G1 | 4 | (4%) | 3 | (3%) |  |
| G2 | 19 | (17%) | 11 | (10%) |  |
| G3 | 68 | (61%) | 86 | (79%) |  |
| Undifferentiated | 7 | (6%) | 5 | (5%) |  |
| Missing info | 13 | (12%) | 4 | (4%) |  |

## Figure S12. Progression-free survival


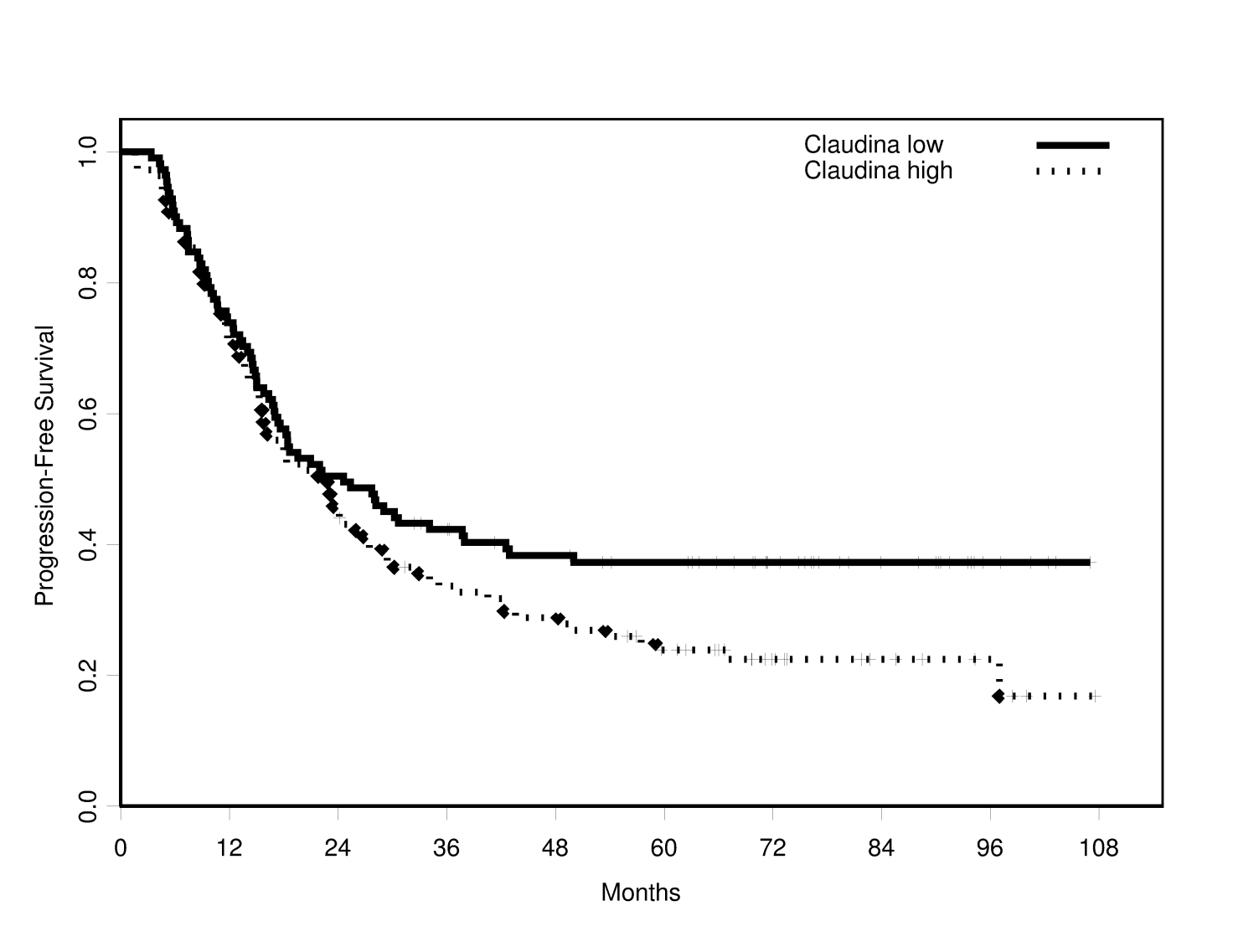


| **Claudin3 expression** | **N. pts** | **Events** | **Median PFS (months)** | **95%CI** |
| --- | --- | --- | --- | --- |
| Low | 111 | 69 | 24.6 | 17.6 - 42.5 |
| High | 109 | 84 | 21.8 | 15.8 - 27.5 |

## Figure S13. Overall survival


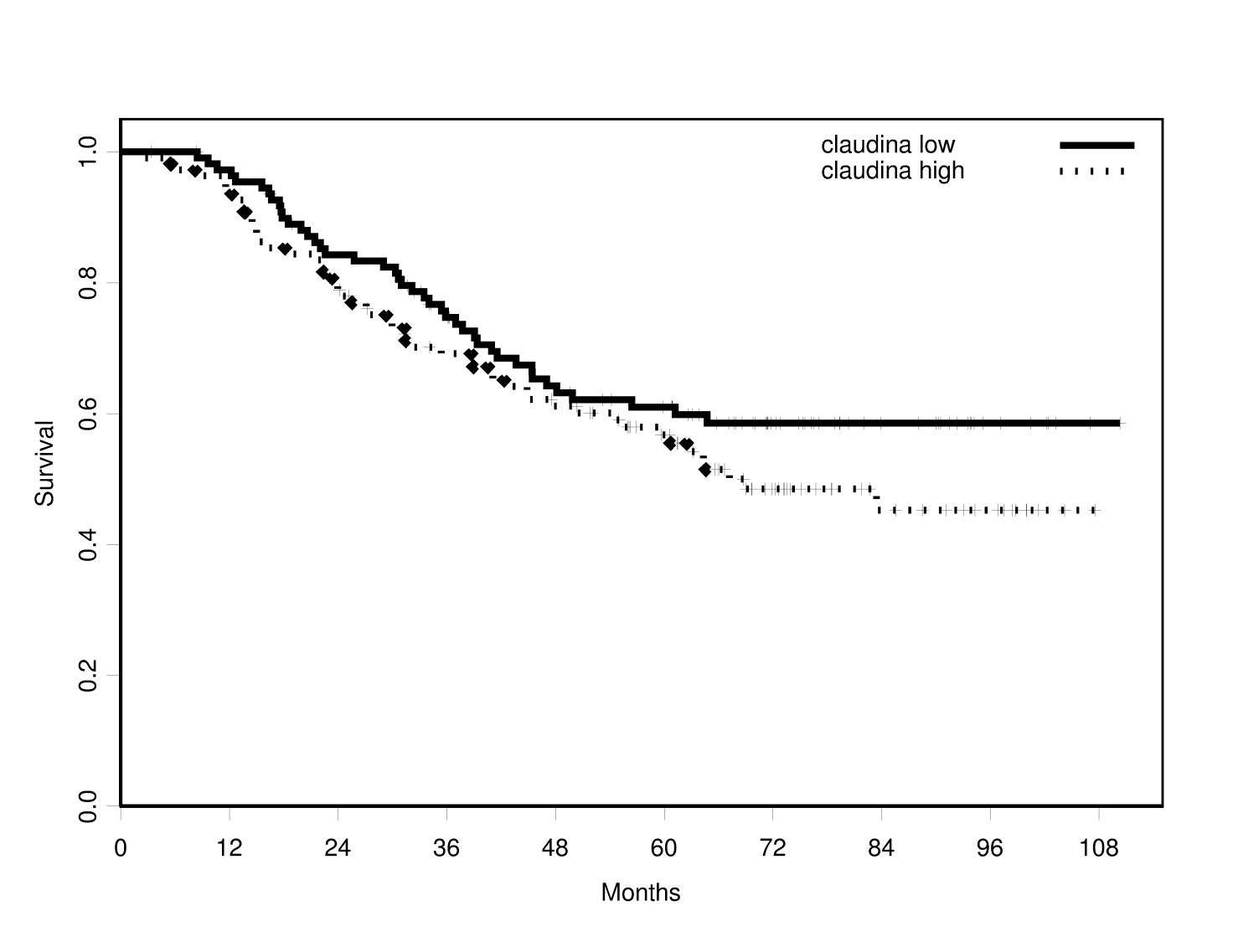


| **Claudin3 expression** | **N. pts** | **Events** | **Median OS (months)** | **95%CI** |
| --- | --- | --- | --- | --- |
| Low | 111 | 42 | n.a. | 64.7 - n.a. |
| High | 109 | 52 | 68.8 | 55.4 - n.a. |

# p53

## Table S10. Characteristics of patients according to p53 category

|  | **p53 low**  **(n=114)** | | **p53 high**  **(n=105)** | | **P value** |
| --- | --- | --- | --- | --- | --- |
| **Median age** (range) | 58 | (28-77) | 57 | (37-76) | 0.11 |
| Age < 70 yrs | 102 | (89%) | 90 | (86%) | 0.40 |
| Age > 70 yrs | 12 | (11%) | 15 | (14%) |  |
| **ECOG performance status** |  |  |  |  | 0.92 |
| 0-1 | 111 | (97%) | 102 | (97%) |  |
| 2 | 3 | (3%) | 3 | (3%) |  |
| **Residual disease** |  |  |  |  | 0.21 |
| None | 50 | (44%) | 41 | (39%) |  |
| ≤ 1 cm | 27 | (24%) | 21 | (20%) |  |
| > 1 cm | 25 | (22%) | 36 | (34%) |  |
| Not operated | 12 | (11%) | 7 | (7%) |  |
| **FIGO stage** |  |  |  |  | 0.06 |
| Ic | 15 | (13%) | 4 | (4%) |  |
| II | 7 | (6%) | 11 | (10%) |  |
| III | 79 | (69%) | 74 | (70%) |  |
| IV | 13 | (11%) | 16 | (15%) |  |
| **Tumor histology** |  |  |  |  | 0.50 |
| Serous | 80 | (70%) | 75 | (71%) |  |
| Endometrioid | 12 | (11%) | 15 | (14%) |  |
| Clear cell | 5 | (4%) | 1 | (1%) |  |
| Mucinous | 1 | (1%) | 1 | (1%) |  |
| Undifferentiated | 5 | (4%) | 8 | (8%) |  |
| Mixed or other | 8 | (7%) | 5 | (5%) |  |
| Missing information | 3 | (3%) | 0 | - |  |
| **Grading** |  |  |  |  | 0.17 |
| G1 | 3 | (3%) | 3 | (3%) |  |
| G2 | 22 | (19%) | 10 | (10%) |  |
| G3 | 74 | (65%) | 77 | (73%) |  |
| Undifferentiated | 5 | (4%) | 8 | (8%) |  |
| Missing information | 10 | (9%) | 7 | (7%) |  |

## Figure S14. Progression-free survival

**
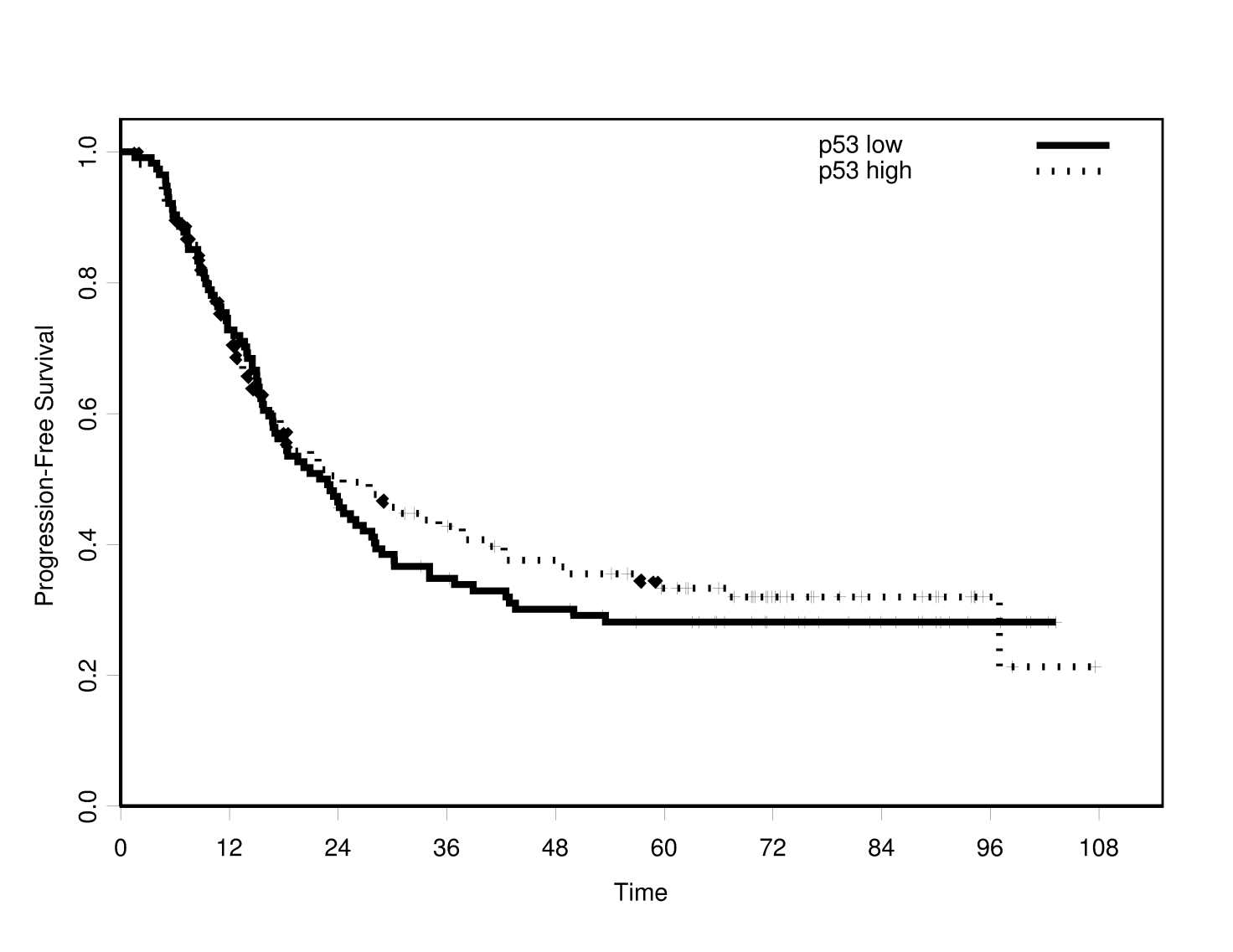
**

| **p53**  **expression** | **N. pts** | **Events** | **Median PFS (months)** | **95%CI** |
| --- | --- | --- | --- | --- |
| Low | 114 | 81 | 22.4 | (16.8 - 28-2) |
| High | 105 | 71 | 24.5 | (17.6 - 42.3) |

## Figure S15. Overall survival

**
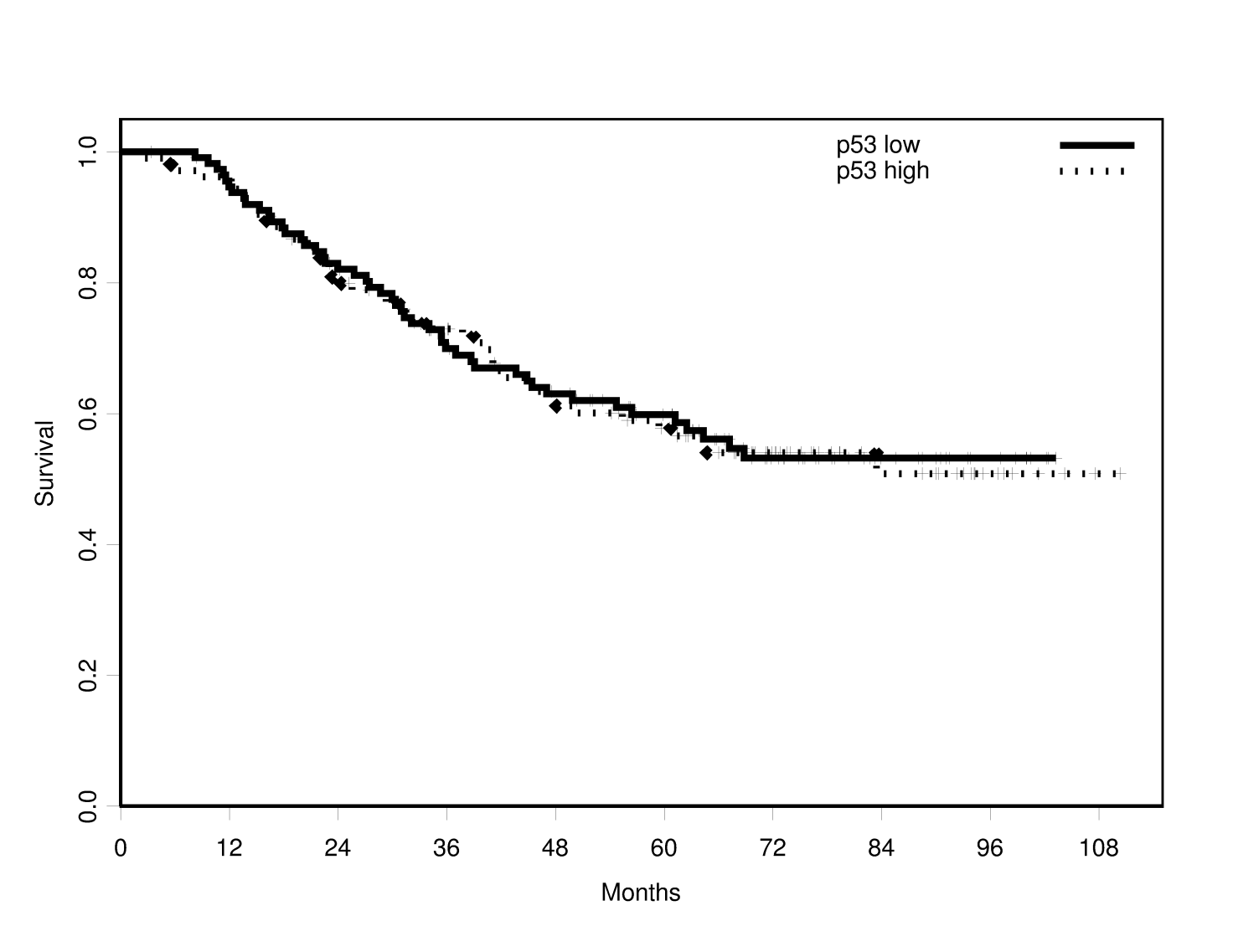
**

| **p53**  **expression** | **N. pts** | **Events** | **Median OS (months)** | **95%CI** |
| --- | --- | --- | --- | --- |
| Low | 114 | 48 | n.a. | 61.2 - n.a. |
| High | 105 | 46 | n.a. | 55.4 - n.a. |

# cFLIP

## Table S11. Characteristics of patients according to FLIP category

|  | **cFLIP negative**  **(n=129)** | | **cFLIP positive**  **(n=71)** | | **P value** |
| --- | --- | --- | --- | --- | --- |
| **Median age** (range) | 57 | (28 - 77) | 58 | (28 - 75) | 0.37 |
| Age < 70 yrs | 117 | (91%) | 59 | (83%) | 0.11 |
| Age > 70 yrs | 12 | (9%) | 12 | (17%) |  |
| **ECOG performance status** |  |  |  |  | 0.25 |
| 0-1 | 127 | (98%) | 68 | (96%) |  |
| 2 | 2 | (2%) | 3 | (4%) |  |
| **Residual disease** |  |  |  |  | 0.61 |
| None | 57 | (44%) | 26 | (37%) |  |
| ≤ 1 cm | 27 | (21%) | 20 | (28%) |  |
| > 1 cm | 36 | (28%) | 19 | (27%) |  |
| Not operated | 9 | (7%) | 6 | (9%) |  |
| **FIGO stage** |  |  |  |  | 0.46 |
| Ic | 8 | (6%) | 9 | (13%) |  |
| II | 11 | (9%) | 5 | (7%) |  |
| III | 94 | (73%) | 48 | (68%) |  |
| IV | 16 | (12%) | 9 | (13%) |  |
| **Tumor histology** |  |  |  |  | 0.99 |
| Serous | 91 | (71%) | 50 | (70%) |  |
| Endometrioid | 18 | (14%) | 9 | (13%) |  |
| Clear cell | 3 | (2%) | 3 | (4%) |  |
| Mucinous | 1 | (1%) | 1 | (1%) |  |
| Undifferentiated | 7 | (5%) | 4 | (6%) |  |
| Mixed or other | 7 | (5%) | 3 | (4%) |  |
| Missing information | 2 | (2%) | 1 | (1%) |  |
| **Grading** |  |  |  |  | 0.39 |
| G1 | 4 | (3%) | 2 | (3%) |  |
| G2 | 15 | (12%) | 14 | (20%) |  |
| G3 | 98 | (76%) | 48 | (68%) |  |
| G4 | 7 | (5%) | 6 | (8%) |  |
| Missing information | 5 | (4%) | 1 | (1%) |  |

## Figure S16. Progression-free survival

**
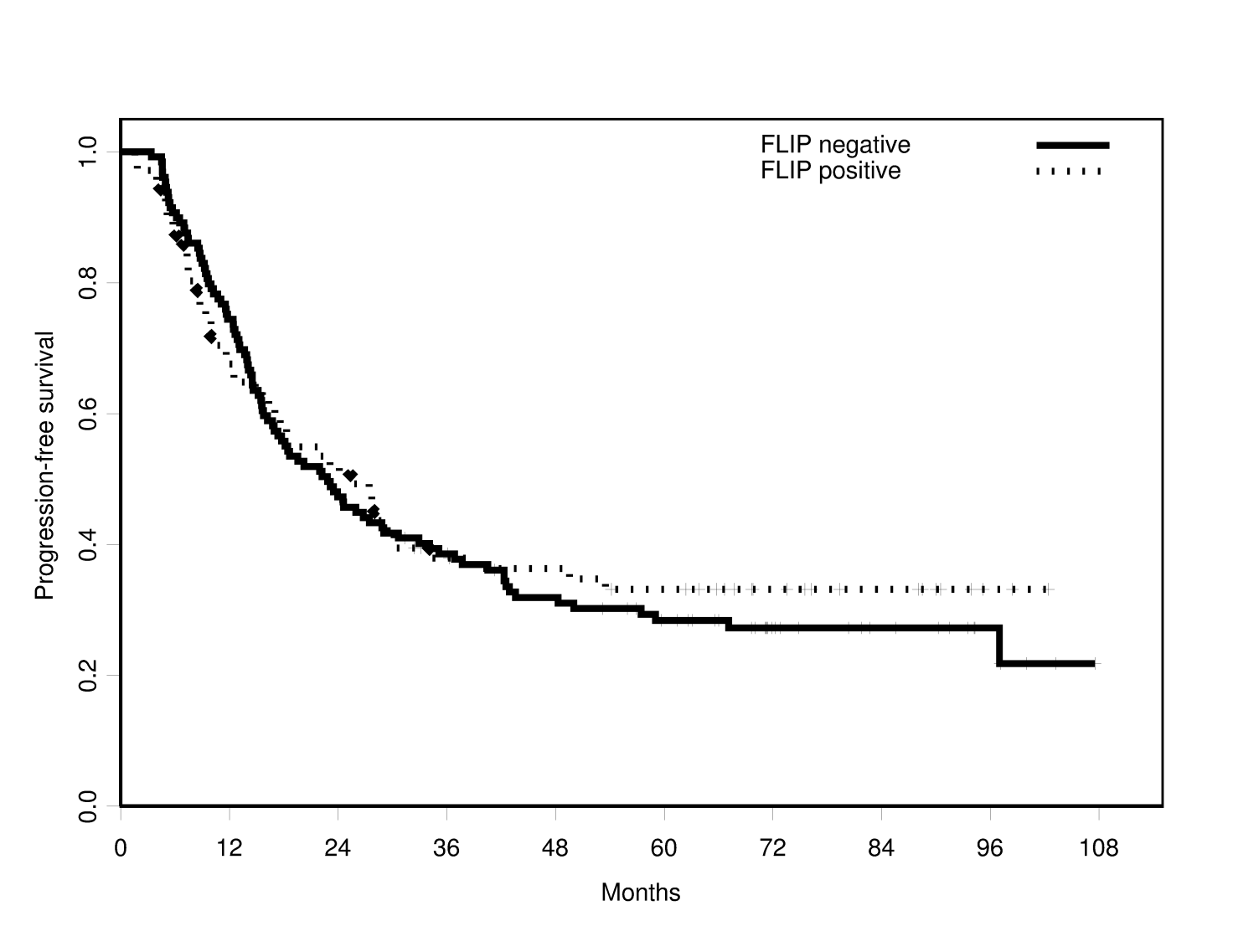
**

| **cFLIP expression** | **N. pts** | **Events** | **Median PFS (months)** | **95%CI** |
| --- | --- | --- | --- | --- |
| Negative | 129 | 93 | 22.8 | 16.9 - 32.9 |
| Positive | 71 | 47 | 25.4 | 17.0 - 38.9 |

## Figure S17. Overall survival

**
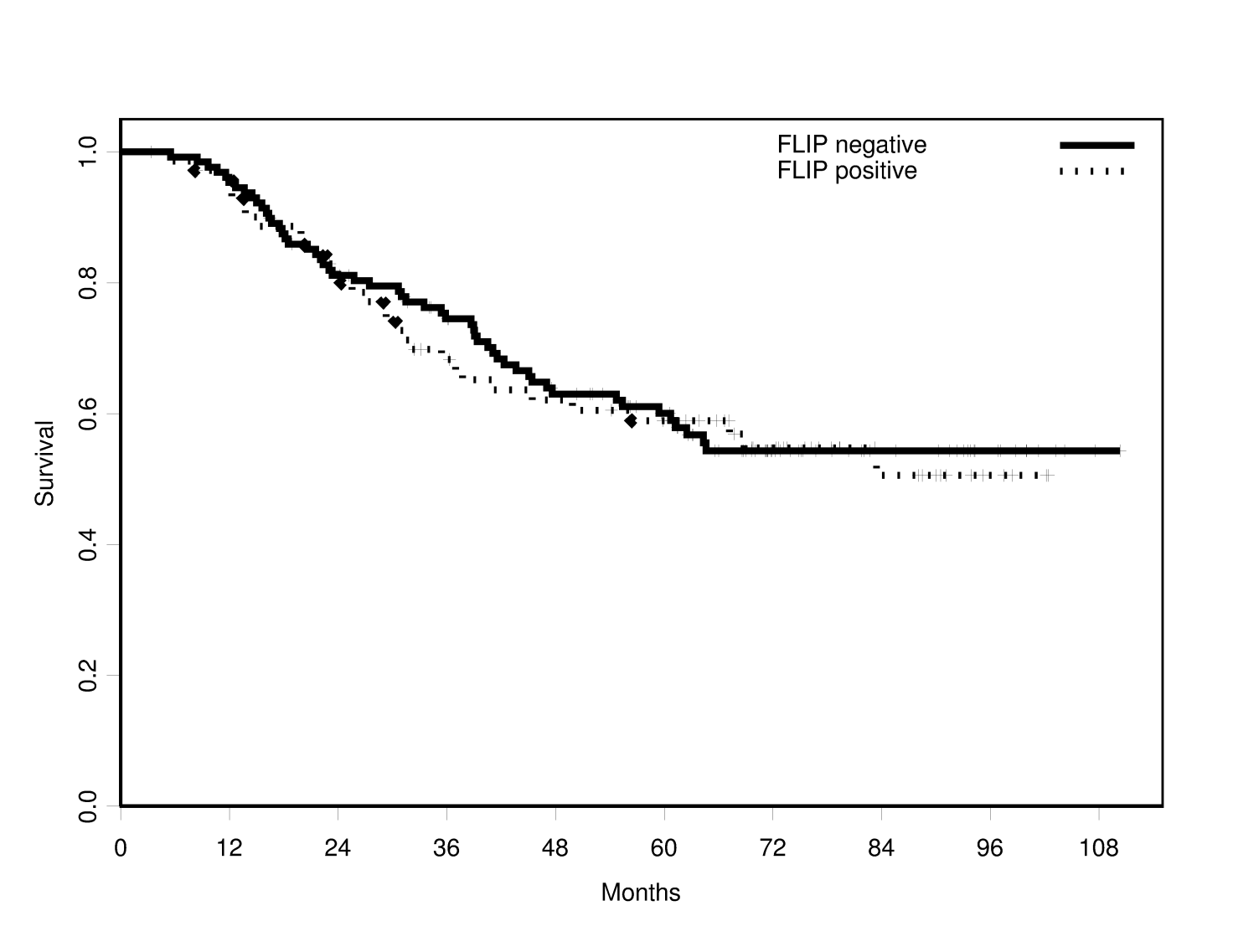
**

| **cFLIP expression** | **N. pts** | **Events** | **Median OS (months)** | **95%CI** |
| --- | --- | --- | --- | --- |
| Negative | 129 | 53 | n.a. | 61.2 - n.a. |
| Positive | 71 | 31 | n.a. | 49.9 - n.a. |

# TRAP1

## Table S12. Characteristics of patients according to TRAP1 category

|  | **TRAP1 neg**  **(n=85)** | | **TRAP1 pos**  **(n=116)** | | **P value** |
| --- | --- | --- | --- | --- | --- |
| **Median age** (range) | 58 | (32-77) | 57 | (28-76) | 0.98 |
| Age < 70 yrs | 75 | (88%) | 99 | (85%) | 0.55 |
| Age > 70 yrs | 10 | (12%) | 17 | (15%) |  |
| **ECOG performance status** |  |  |  |  | 0.65 |
| 0-1 | 83 | (98%) | 112 | (97%) |  |
| 2 | 2 | (2%) | 4 | (3%) |  |
| **Residual disease** |  |  |  |  | 0.87 |
| None | 37 | (44%) | 51 | (44%) |  |
| ≤ 1 cm | 20 | (24%) | 25 | (22%) |  |
| > 1 cm | 25 | (29%) | 33 | (28%) |  |
| Not operated | 3 | (4%) | 7 | (6%) |  |
| **FIGO stage** |  |  |  |  | 0.67 |
| Ic | 9 | (11%) | 11 | (10%) |  |
| II | 9 | (11%) | 8 | (7%) |  |
| III | 57 | (67%) | 78 | (67%) |  |
| IV | 10 | (12%) | 19 | (16%) |  |
| **Tumor histology** |  |  |  |  | 0.03 |
| Serous | 54 | (64%) | 90 | (78%) |  |
| Endometrioid | 14 | (16%) | 13 | (11%) |  |
| Clear cell | 2 | (2%) | 4 | (3%) |  |
| Mucinous | 1 | (1%) | 1 | (1%) |  |
| Undifferentiated | 7 | (8%) | - | - |  |
| Mixed or other | 6 | (7%) | 6 | (5%) |  |
| Missing information | 1 | (1%) | 2 | (2%) |  |
| **Grading** |  |  |  |  | 0.002 |
| G1 | 3 | (4%) | 3 | (3%) |  |
| G2 | 18 | (21%) | 13 | (11%) |  |
| G3 | 53 | (62%) | 91 | (78%) |  |
| Undifferentiated | 7 | (8%) | - | - |  |
| Missing information | 4 | (5%) | 9 | (8%) |  |

## Figure S18. Progression-free survival

**
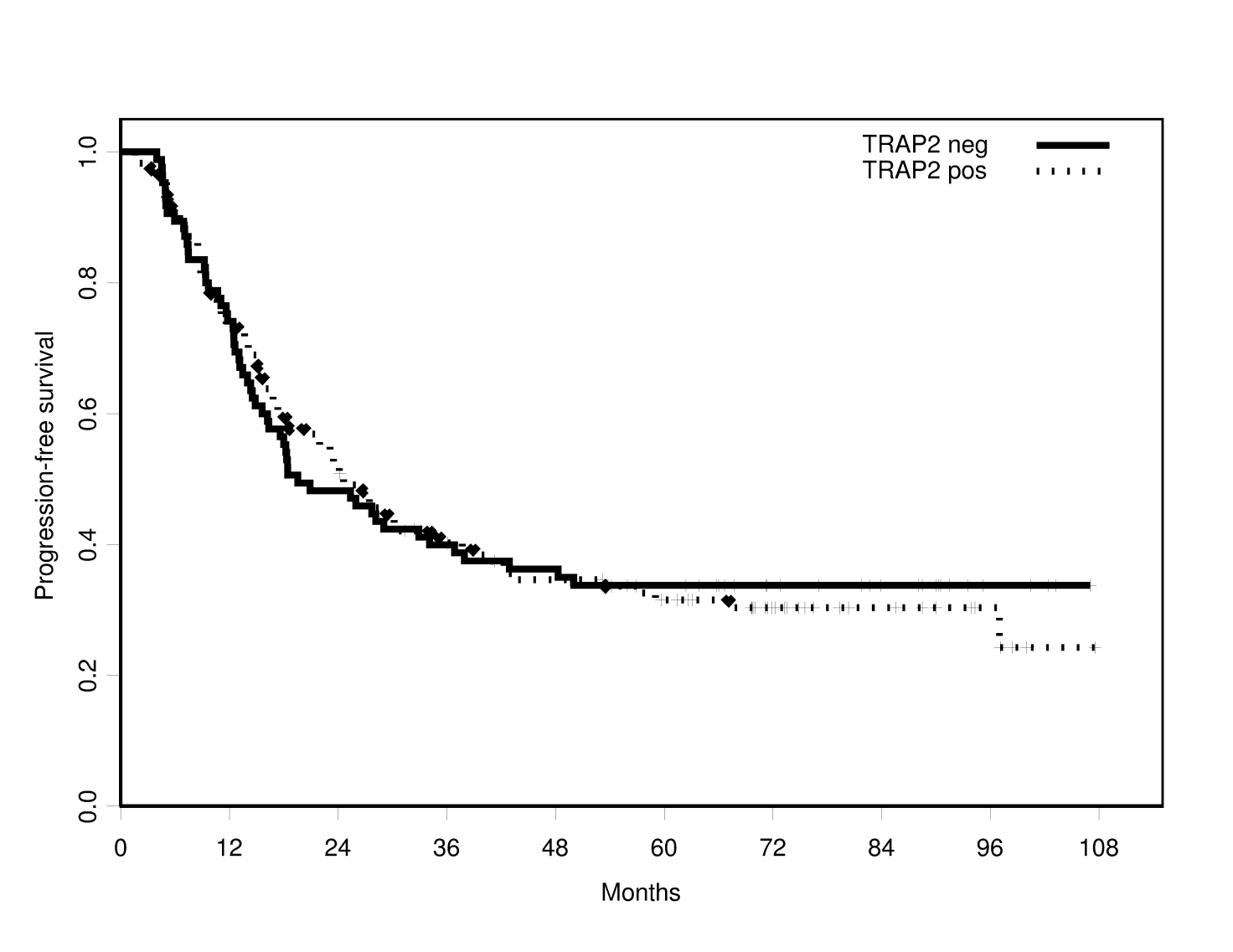
**

| **TRAP1 expression** | **N. pts** | **Events** | **Median PFS**  **(months)** | **95%CI** |
| --- | --- | --- | --- | --- |
| Negative | 85 | 56 | 19.6 | 16.2 - 38.0 |
| Positive | 116 | 80 | 24.5 | 18.7 - 37-7 |

## Figure S19. Overall survival

**
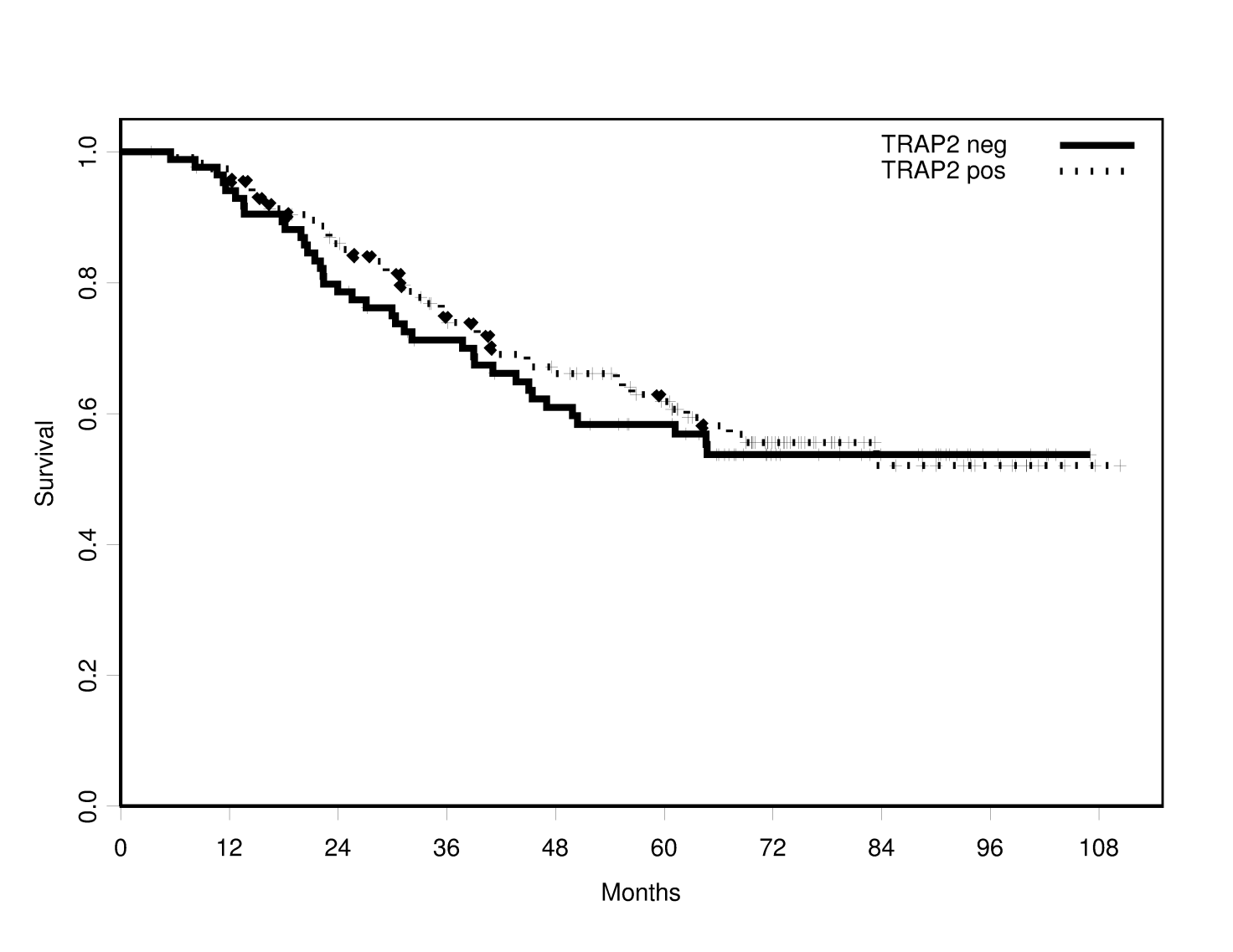
**

| **TRAP1 expression** | **N. pts** | **Events** | **Median OS (months)** | **95%CI** |
| --- | --- | --- | --- | --- |
| Negative | 85 | 37 | n.a. | 49.9 - n.a. |
| Positive | 116 | 47 | n.a. | 64.3 - n.a. |

# BAG3

## Table S13. Characteristics of patients according to BAG3 category

|  | **BAG3 0-1**  **(n=44)** | | **BAG3 2**  **(n=45)** | | **BAG3 3**  **(n=123)** | | **P value** |
| --- | --- | --- | --- | --- | --- | --- | --- |
| **Median age** (range) | 60 | (36-74) | 58 | (34-74) | 56 | (28-77) | 0.14 |
| Age < 70 yrs | 38 | (86%) | 39 | (87%) | 109 | (89%) | 0.90 |
| Age > 70 yrs | 6 | (14%) | 6 | (13%) | 14 | (11%) |  |
| **ECOG performance status** |  |  |  |  |  |  | 0.10 |
| 0-1 | 42 | (95%) | 42 | (93%) | 122 | (99%) |  |
| 2 | 2 | (5%) | 3 | (7%) | 1 | (1%) |  |
| **Residual disease** |  |  |  |  |  |  | 0.35 |
| None | 17 | (39%) | 25 | (56%) | 45 | (37%) |  |
| ≤ 1 cm | 9 | (20%) | 8 | (18%) | 31 | (25%) |  |
| > 1 cm | 14 | (32%) | 8 | (18%) | 39 | (32%) |  |
| Not operated | 4 | (9%) | 4 | (9%) | 8 | (7%) |  |
| **FIGO stage** |  |  |  |  |  |  | 0.15 |
| Ic | 3 | (7%) | 8 | (18%) | 7 | (6%) |  |
| II | 5 | (11%) | 3 | (7%) | 9 | (7%) |  |
| III | 28 | (64%) | 26 | (58%) | 92 | (75%) |  |
| IV | 8 | (18%) | 8 | (18%) | 15 | (12%) |  |
| **Tumor histology** |  |  |  |  |  |  | 0.32 |
| Serous | 27 | (61%) | 28 | (62%) | 93 | (76%) |  |
| Endometrioid | 10 | (23%) | 8 | (18%) | 9 | (7%) |  |
| Clear cell | 1 | (2%) | 1 | (2%) | 5 | (4%) |  |
| Mucinous | - | - | - | - | 2 | (2%) |  |
| Undifferentiated | 3 | (7%) | 3 | (7%) | 7 | (6%) |  |
| Mixed or other | 3 | (7%) | 4 | (9%) | 6 | (5%) |  |
| Missing info | - | - | 1 | (2%) | 1 | (1%) |  |
| **Grading** |  |  |  |  |  |  | 0.56 |
| G1 | 1 | (2%) | 3 | (7%) | 3 | (2%) |  |
| G2 | 8 | (18%) | 8 | (18%) | 14 | (11%) |  |
| G3 | 30 | (68%) | 26 | (58%) | 91 | (74%) |  |
| Undifferentiated | 3 | (7%) | 3 | (7%) | 7 | (6%) |  |
| Missing info | 2 | (5%) | 5 | (11%) | 8 | (7%) |  |

## Figure S20. Progression-free survival

**
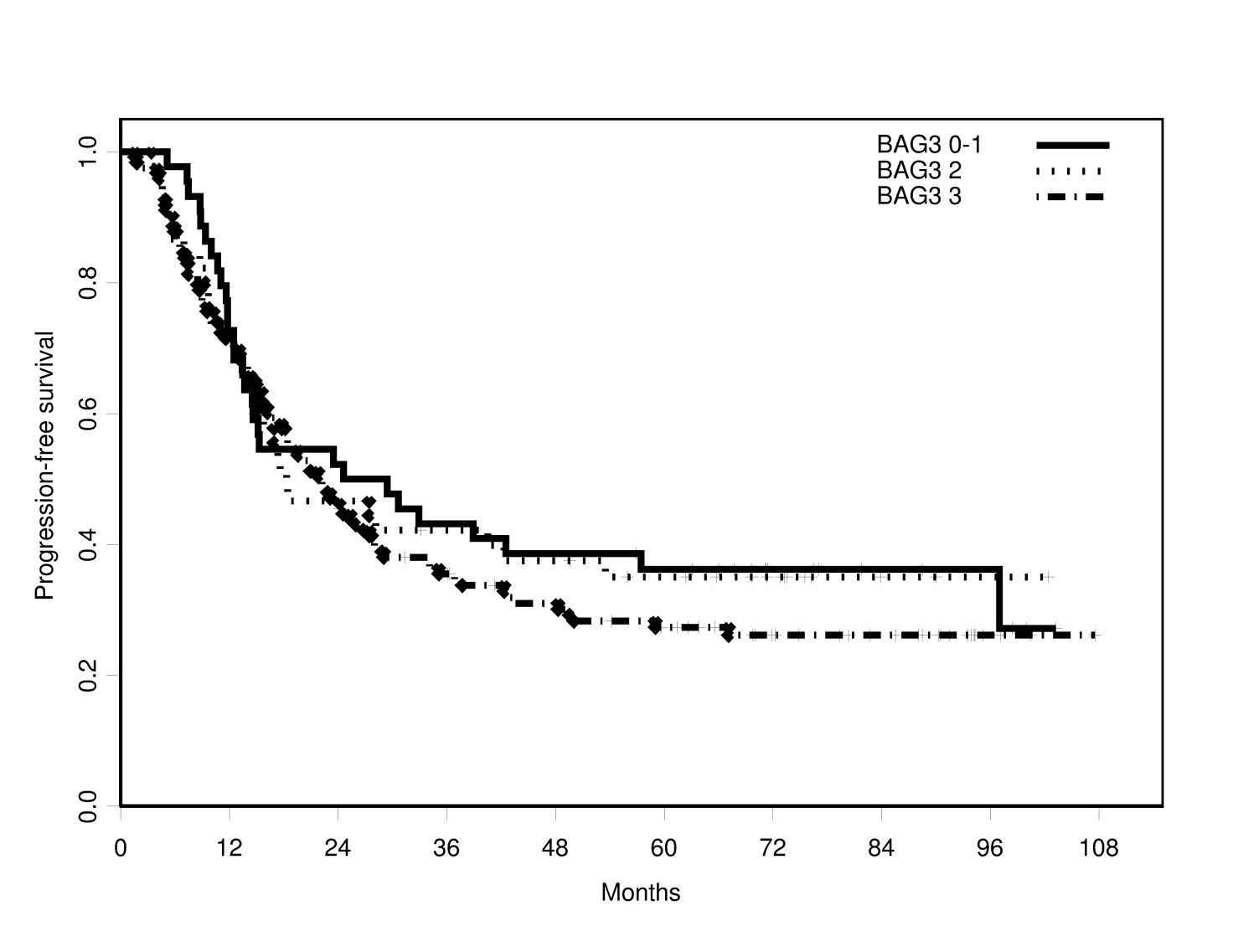
**

| **BAG3 expression** | **N. pts** | **Events** | **Median PFS (months)** | **95%CI** |
| --- | --- | --- | --- | --- |
| 0-1 | 44 | 29 | 29.4 | 14.5 - n.a. |
| 2 | 45 | 29 | 18.3 | 15.0 - n.a. |
| 3 | 123 | 89 | 22.0 | 17.8 - 28.0 |

## Figure S21. Overall survival

**
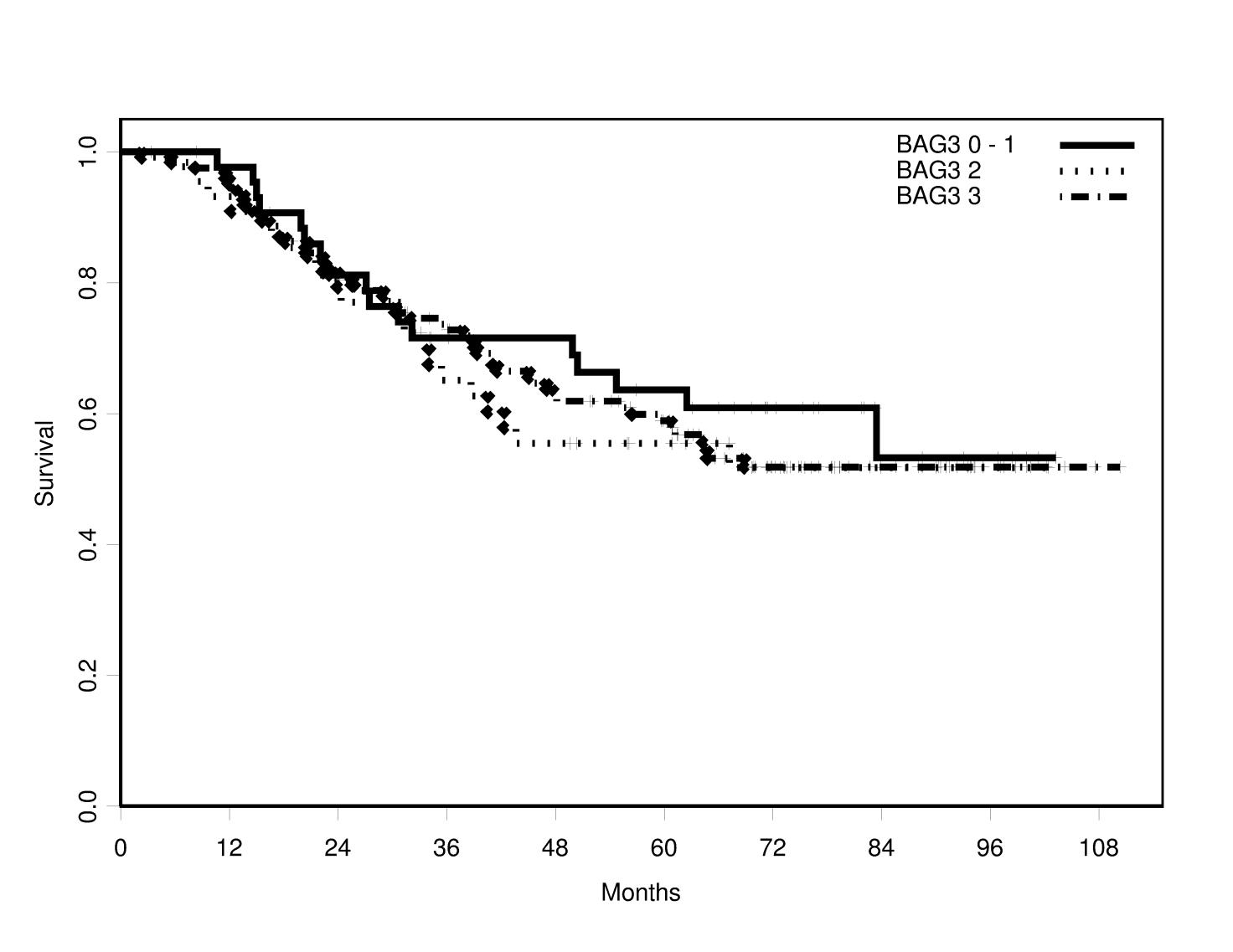
**

| **BAG3**  **expression** | **N. pts** | **Events** | **Median OS (months)** | **95%CI** |
| --- | --- | --- | --- | --- |
| 0-1 | 44 | 17 | n.a. | 62.5 - n.a. |
| 2 | 45 | 20 | n.a. | 38.7 - n.a. |
| 3 | 123 | 54 | n.a. | 60.7 - n.a. |

# HOXB13 (cytoplasm)

## Table S14. Characteristics of patients according to HOXB13 (cytoplasm) category

|  | **HOXB13**  **Cytoplasm negative**  **(n=109)** | | **HOXB13**  **Cytoplasm positive**  **(n=110)** | | **P value** |
| --- | --- | --- | --- | --- | --- |
| **Median age** (range) | 58 | (28-76) | 58 | (30-77) | 0.99 |
| Age < 70 yrs | 94 | (86%) | 97 | (88%) | 0.67 |
| Age > 70 yrs | 15 | (14%) | 13 | (12%) |  |
| **ECOG performance status** | |  |  |  | 0.69 |
| 0-1 | 105 | (96%) | 107 | (97%) |  |
| 2 | 4 | (4%) | 3 | (3%) |  |
| **Residual disease** |  |  |  |  | 0.24 |
| None | 48 | (44%) | 41 | (37%) |  |
| ≤ 1 cm | 26 | (24%) | 22 | (20%) |  |
| > 1 cm | 25 | (23%) | 39 | (35%) |  |
| Not operated | 10 | (9%) | 8 | (7%) |  |
| **FIGO stage** |  |  |  |  | 0.46 |
| Ic | 11 | (10%) | 8 | (7%) |  |
| II | 9 | (8%) | 9 | (8%) |  |
| III | 77 | (71%) | 73 | (66%) |  |
| IV | 12 | (11%) | 20 | (18%) |  |
| **Tumor histology** |  |  |  |  | 0.26 |
| Serous | 76 | (70%) | 78 | (71%) |  |
| Endometrioid | 13 | (12%) | 15 | (14%) |  |
| Clear cell | 2 | (2%) | 5 | (5%) |  |
| Mucinous | 1 | (1%) | - | - |  |
| Undifferentiated | 10 | (9%) | 3 | (3%) |  |
| Mixed or other | 6 | (6%) | 8 | (7%) |  |
| Missing info | 1 | (1%) | 1 | (1%) |  |
| **Grading** |  |  |  |  | 0.06 |
| G1 | 4 | (4%) | 3 | (3%) |  |
| G2 | 19 | (17%) | 12 | (11%) |  |
| G3 | 67 | (61%) | 85 | (77%) |  |
| Undifferentiated | 10 | (9%) | 3 | (3%) |  |
| Missing info | 9 | (8%) | 7 | (6%) |  |

## Figure S22. Progression-free survival


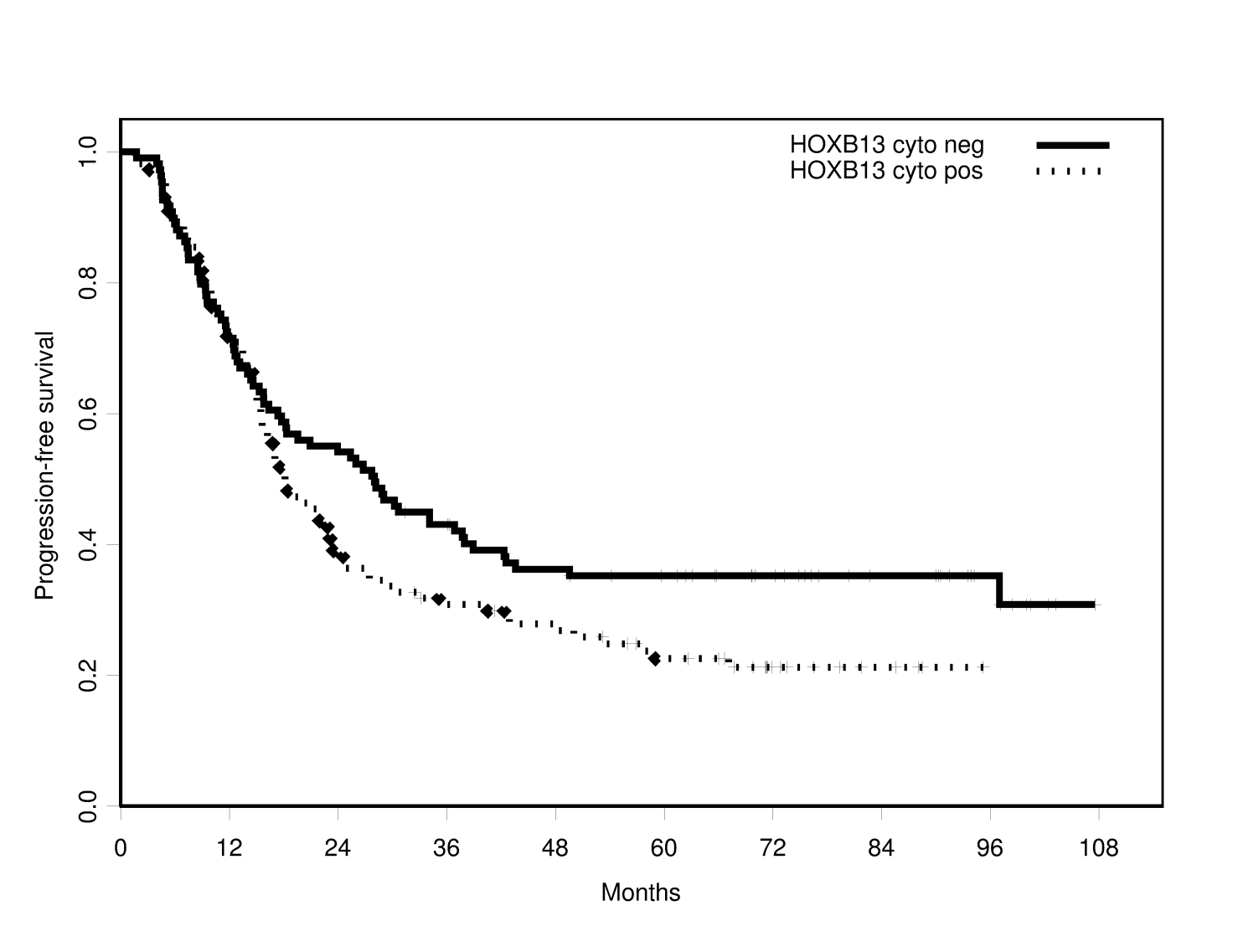


| **HOXB13**  **expression**  **(cytoplasm)** | **N. pts** | **Events** | **Median PFS (months)** | **95%CI** |
| --- | --- | --- | --- | --- |
| Negative | 109 | 71 | 28.0 | 18.2 - 38.9 |
| Positive | 110 | 85 | 18.4 | 15.6 - 23.5 |

## Figure S23. Overall survival

**
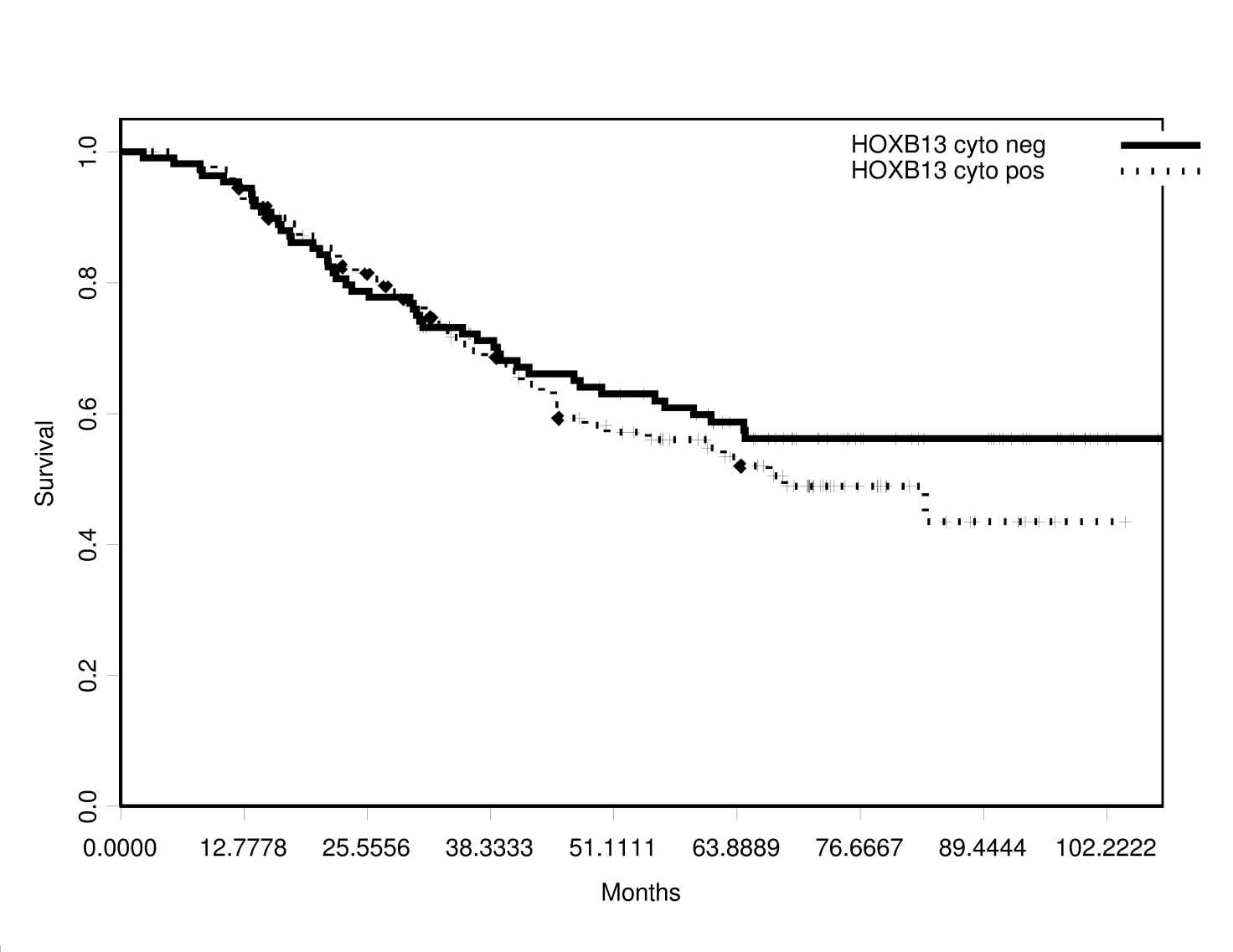
**

| **HOXB13 expression (cytoplasm)** | **N. pts** | **Events** | **Median OS (months)** | **95%CI** |
| --- | --- | --- | --- | --- |
| Negative | 109 | 45 | n.a. | 61.2 - n.a. |
| Positive | 110 | 51 | 68.8 | 48.1 - n.a. |

# HOXB13 (nucleus)

## Table S15. Characteristics of patients according to HOX B13 (nuclear expression) category

|  | **HOXB13**  **Nucleus negative**  **(n=117)** | | **HOXB13**  **Nucleus positive**  **(n=102)** | | **P value** |
| --- | --- | --- | --- | --- | --- |
| **Median age** (range) | 57 | (28-77) | 58 | (28-76) | 0.28 |
| Age < 70 yrs | 104 | (89%) | 87 | (85%) | 0.43 |
| Age > 70 yrs | 13 | (11%) | 15 | (15%) |  |
| **ECOG performance status** | | |  |  | 0.84 |
| 0-1 | 113 | (97%) | 99 | (97%) |  |
| 2 | 4 | (3%) | 3 | (3%) |  |
| **Residual disease** |  |  |  |  | 0.77 |
| None | 47 | (40%) | 42 | (41%) |  |
| ≤ 1 cm | 25 | (21%) | 23 | (23%) |  |
| > 1 cm | 37 | (32%) | 27 | (26%) |  |
| Not operated | 8 | (7%) | 10 | (10%) |  |
| **FIGO stage** |  |  |  |  | 0.73 |
| Ic | 12 | (10%) | 7 | (7%) |  |
| II | 8 | (7%) | 10 | (10%) |  |
| III | 80 | (68%) | 70 | (69%) |  |
| IV | 17 | (15%) | 15 | (15%) |  |
| **Tumor histology** |  |  |  |  | 0.05 |
| Serous | 81 | (69%) | 73 | (72%) |  |
| Endometrioid | 10 | (9%) | 18 | (18%) |  |
| Clear cell | 3 | (3%) | 4 | (4%) |  |
| Mucinous | 1 | (1%) | - | - |  |
| Undifferentiated | 10 | (9%) | 3 | (3%) |  |
| Mixed or other | 11 | (9%) | 3 | (3%) |  |
| Missing info | 1 | (1%) | 1 | (1%) |  |
| **Grading** |  |  |  |  | 0.19 |
| G1 | 4 | (3%) | 3 | (3%) |  |
| G2 | 19 | (16%) | 12 | (12%) |  |
| G3 | 75 | (64%) | 77 | (75%) |  |
| Undifferentiated | 10 | (9%) | 3 | (3%) |  |
| Missing info | 9 | (8%) | 7 | (7%) |  |

## Figure S24. Progression-free survival


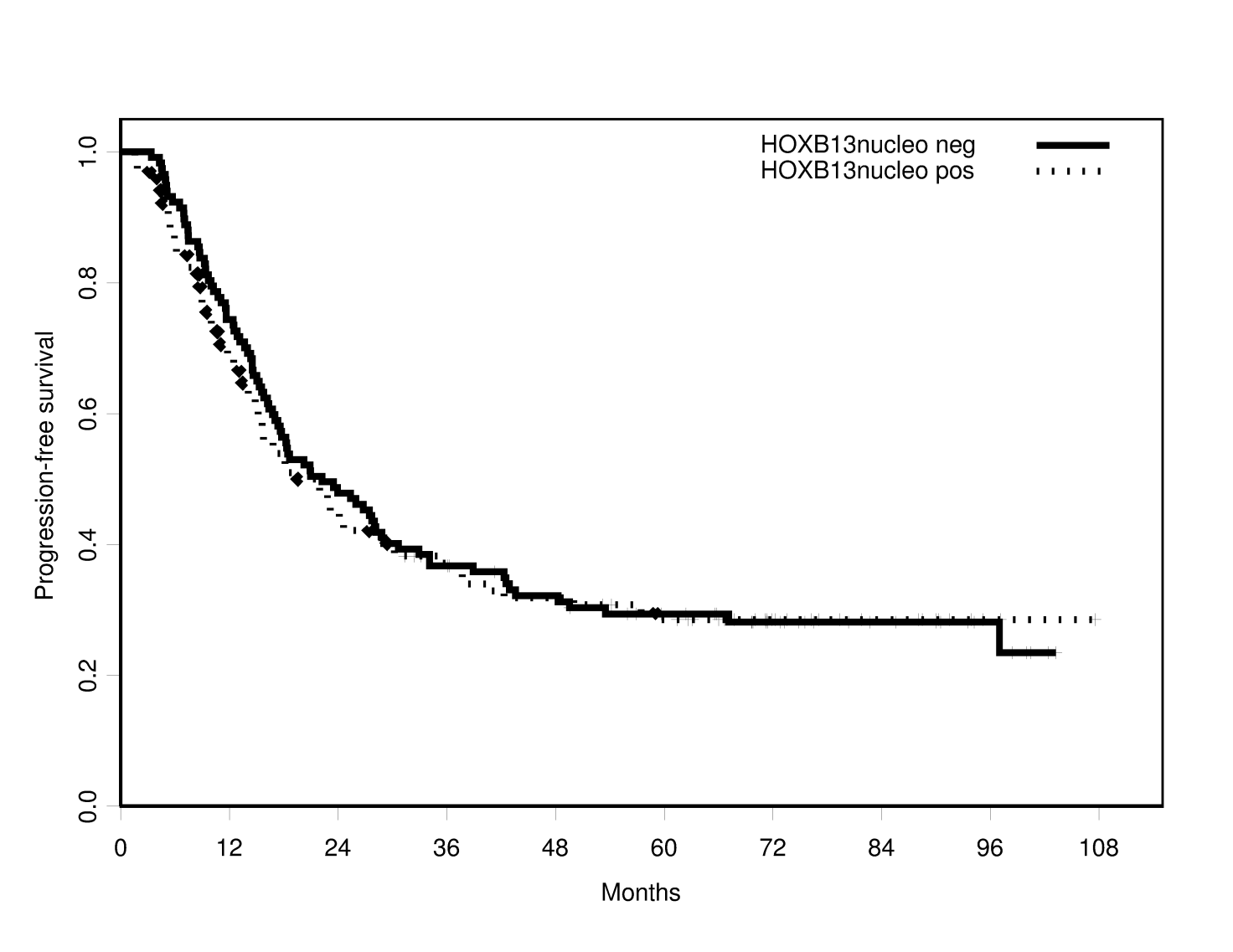


| **HOXB13**  **Nuclear expression** | **N. pts** | **Events** | **Median PFS (months)** | **95%CI** |
| --- | --- | --- | --- | --- |
| Negative | 117 | 84 | 22.2 | 17.4 - 30.7 |
| Positive | 102 | 72 | 20.7 | 15.6 - 30.2 |

## Figure S25. Overall survival

**
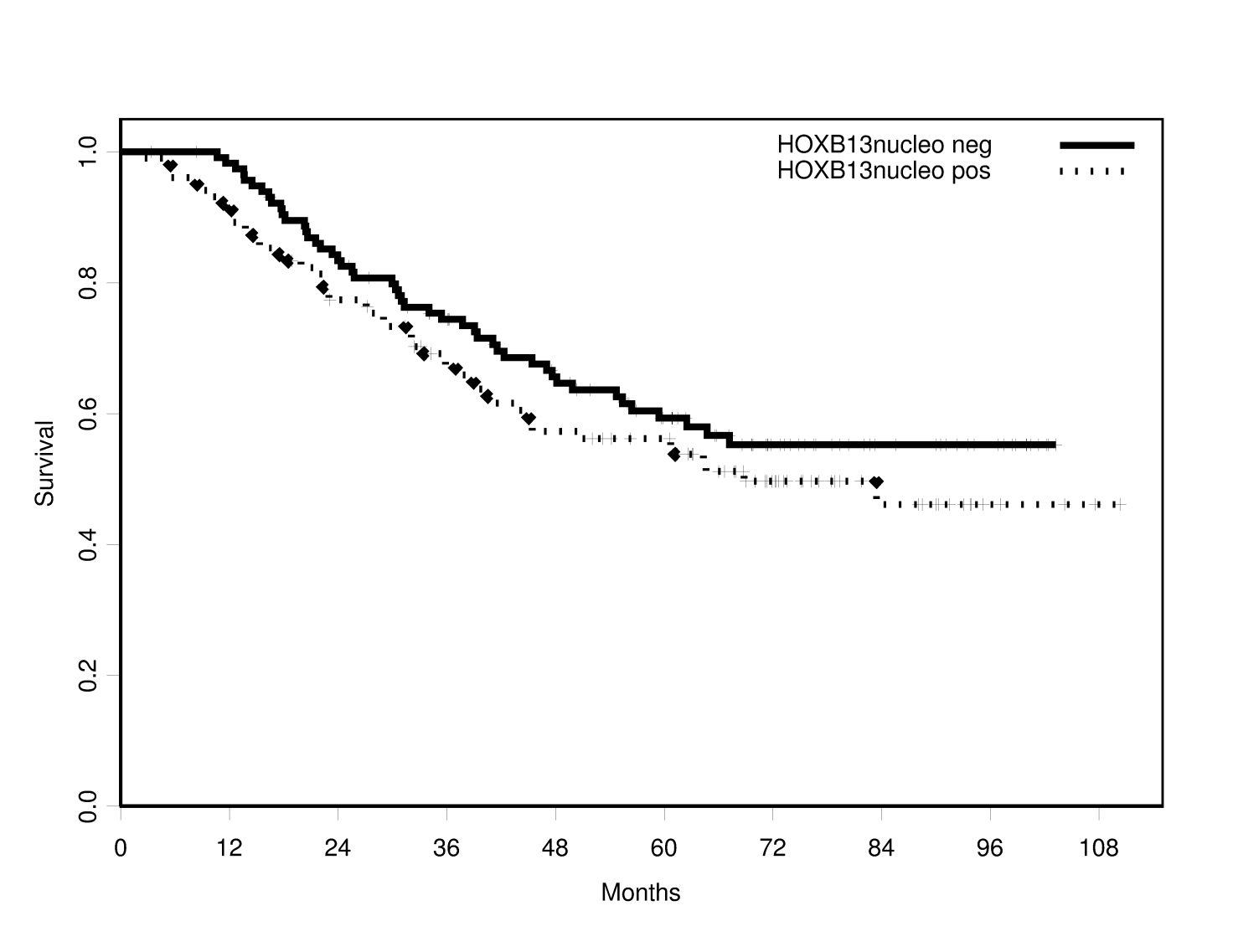
**

| **HOXB13 Nuclear expression** | **N. pts** | **Events** | **Median OS (months)** | **95%CI** |
| --- | --- | --- | --- | --- |
| Negative | 117 | 47 | n.a. | 62.5 - n.a. |
| Positive | 102 | 49 | 68.8 | 45.1 - n.a. |

# HMGA2

## Table S16. Characteristics of patients according to HMGA2 category

|  | **HMGA2 0-1**  **(n=163)** | | **HMGA2 2-3**  **(n=58)** | | **P value** |
| --- | --- | --- | --- | --- | --- |
| **Median age** (range) | 56 | (28-77) | 60 | (36-72) | 0.07 |
| Age < 70 yrs | 141 | (87%) | 53 | (91%) | 0.33 |
| Age > 70 yrs | 22 | (13%) | 5 | (9%) |  |
| **ECOG performance status** |  |  |  |  | 0.59 |
| 0-1 | 158 | (97%) | 57 | (98%) |  |
| 2 | 5 | (3%) | 1 | (2%) |  |
| **Residual disease** |  |  |  |  | 0.03 |
| None | 70 | (43%) | 22 | (38%) |  |
| ≤ 1 cm | 30 | (18%) | 19 | (33%) |  |
| > 1 cm | 52 | (32%) | 10 | (17%) |  |
| Not operated | 11 | (7%) | 7 | (12%) |  |
| **FIGO stage** |  |  |  |  | 0.35 |
| Ic | 12 | (7%) | 7 | (12%) |  |
| II | 16 | (10%) | 2 | (3%) |  |
| III | 112 | (69%) | 41 | (71%) |  |
| IV | 23 | (14%) | 8 | (14%) |  |
| **Tumor histology** |  |  |  |  | 0.79 |
| Serous | 115 | (71%) | 40 | (69%) |  |
| Endometrioid | 21 | (13%) | 6 | (10%) |  |
| Clear cell | 6 | (4%) | 1 | (2%) |  |
| Mucinous | 1 | (1%) | 1 | (2%) |  |
| Undifferentiated | 8 | (5%) | 5 | (9%) |  |
| Mixed or other | 10 | (6%) | 4 | (7%) |  |
| Missing information | 2 | (1%) | 1 | (2%) |  |
| **Grading** |  |  |  |  | 0.41 |
| G1 | 3 | (2%) | 2 | (3%) |  |
| G2 | 22 | (13%) | 10 | (17%) |  |
| G3 | 119 | (73%) | 35 | (60%) |  |
| Undifferentiated | 8 | (5%) | 5 | (9%) |  |
| Missing information | 11 | (7%) | 6 | (10%) |  |

## Figure S26. Progression-free survival

**
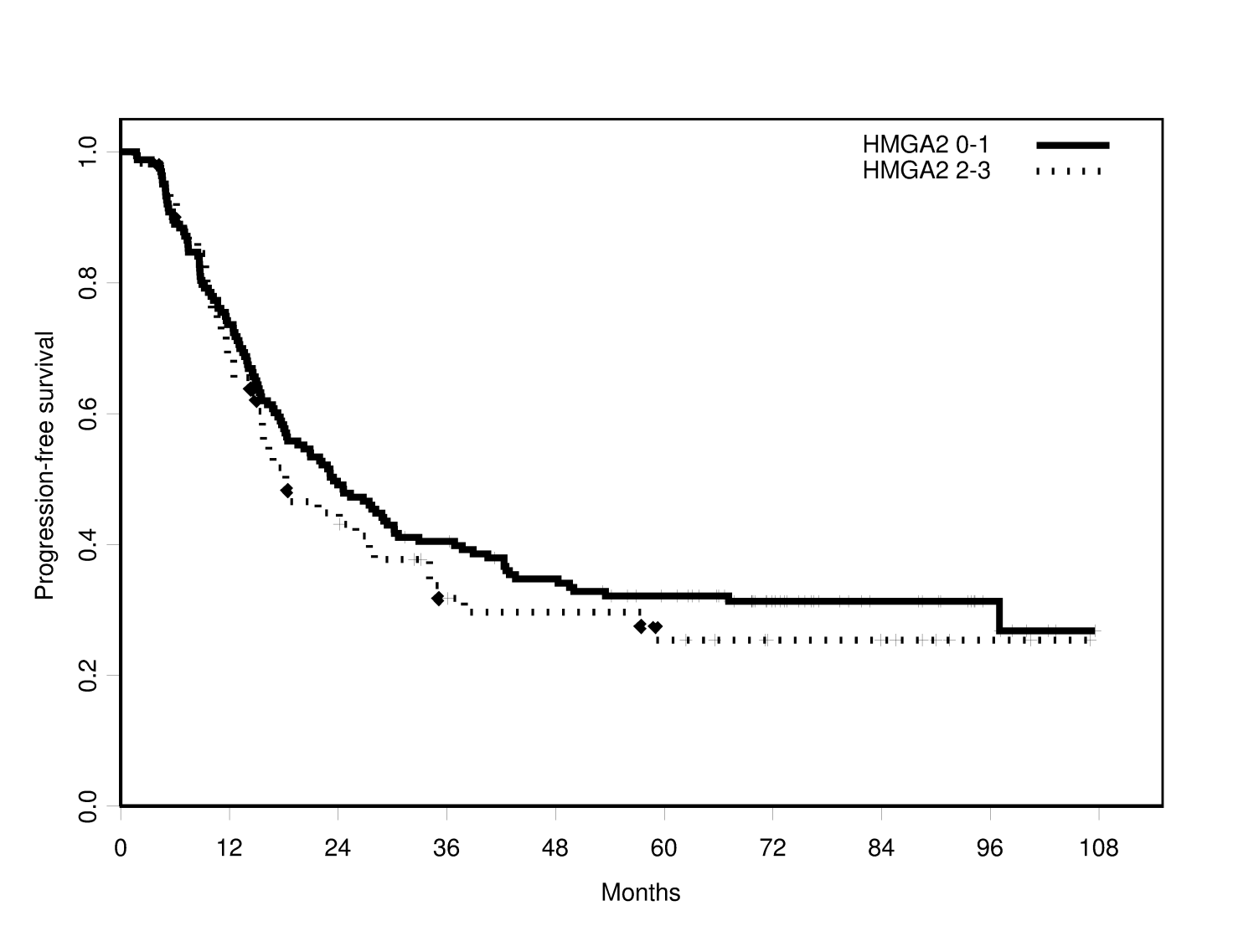
**

| **HMGA2 expression** | **N. pts** | **Events** | **Median PFS (months)** | **95%CI** |
| --- | --- | --- | --- | --- |
| 0-1 | 163 | 112 | 23.5 | 18.2 - 30.7 |
| 2-3 | 58 | 42 | 18.2 | 15.0 - 34.1 |

## Figure S27. Overall survival

**
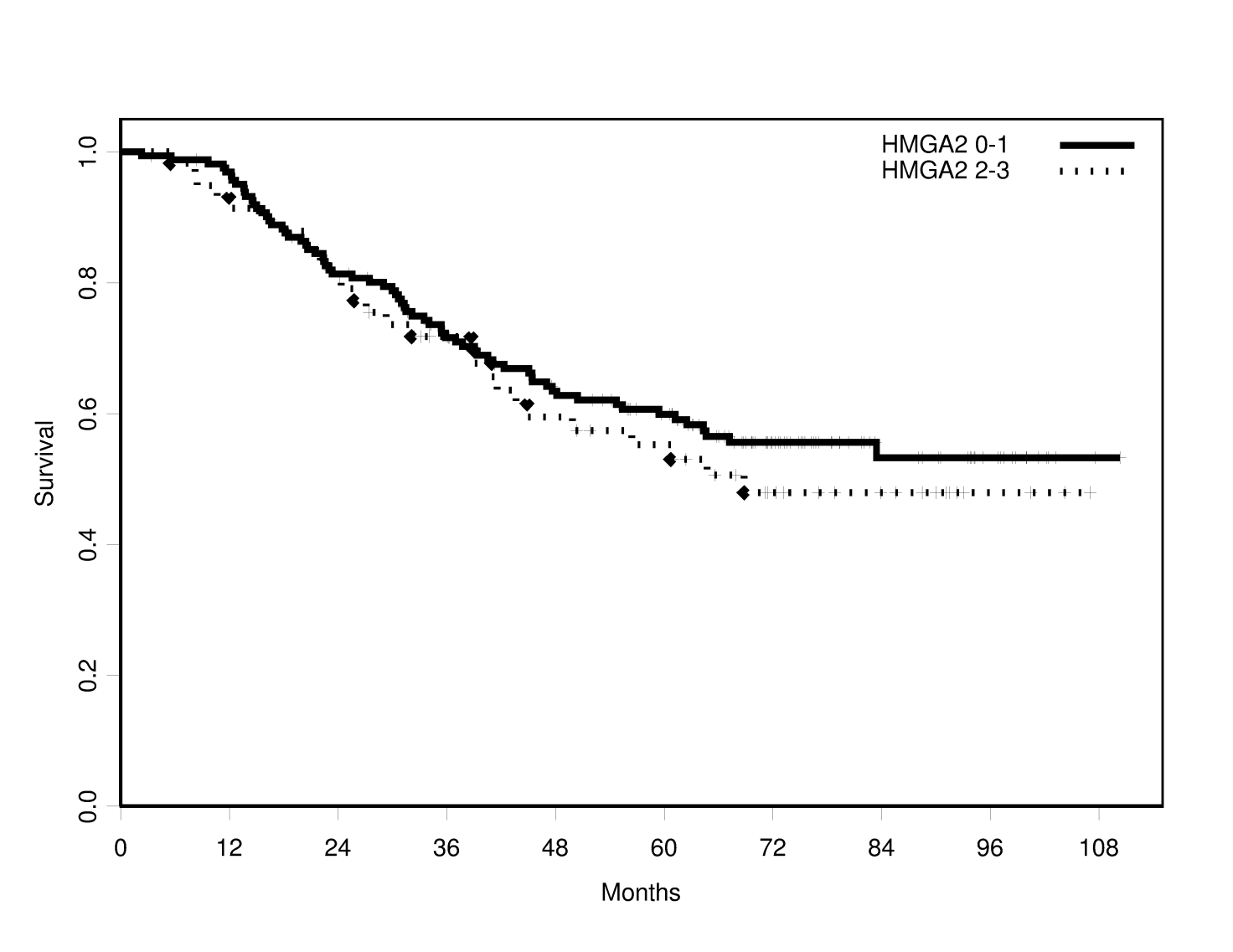
**

| **HMGA2 Expression** | **N. pts** | **Events** | **Median OS (months)** | **95%CI** |
| --- | --- | --- | --- | --- |
| 0-1 | 163 | 68 | n.a. | 64.3 - n.a. |
| 2-3 | 58 | 27 | 68.8 | 43.7 - n.a. |

# CDK6 (intensity)

## Table S17. Characteristics of patients according to CDK6 intensity category

|  | **CDK6 low/moderate intensity**  **(n=47)** | | **CDK6**  **high**  **intensity**  **(n=105)** | | **CDK6**  **very high intensity**  **(n=71)** | | **P value** |
| --- | --- | --- | --- | --- | --- | --- | --- |
| **Median age** (range) | 58 | (37-76) | 57 | (28-77) | 55 | (30-75) | 0.70 |
| Age < 70 yrs | 42 | (89%) | 90 | (86%) | 65 | (92%) | 0.48 |
| Age > 70 yrs | 5 | (11%) | 15 | (14%) | 6 | (8%) |  |
| **ECOG performance status** |  |  |  |  |  |  | 0.60 |
| 0-1 | 46 | (98%) | 101 | (96%) | 70 | (99%) |  |
| 2 | 1 | (2%) | 4 | (4%) | 1 | (1%) |  |
| **Residual disease** |  |  |  |  |  |  | 0.28 |
| None | 19 | (40%) | 46 | (44%) | 28 | (39%) |  |
| ≤ 1 cm | 9 | (19%) | 28 | (27%) | 12 | (17%) |  |
| > 1 cm | 13 | (28%) | 27 | (26%) | 23 | (32%) |  |
| Not operated | 6 | (13%) | 4 | (4%) | 8 | (11%) |  |
| **FIGO stage** |  |  |  |  |  |  | 0.08 |
| Ic | 3 | (6%) | 12 | (11%) | 5 | (7%) |  |
| II | 4 | (9%) | 11 | (10%) | 3 | (4%) |  |
| III | 30 | (64%) | 75 | (71%) | 49 | (69%) |  |
| IV | 10 | (21%) | 7 | (7%) | 14 | (20%) |  |
| **Tumor histology** |  |  |  |  |  |  | 0.18 |
| Serous | 28 | (60%) | 74 | (70%) | 55 | (77%) |  |
| Endometrioid | 7 | (15%) | 13 | (12%) | 7 | (10%) |  |
| Clear cell | 1 | (2%) | 5 | (5%) | 1 | (1%) |  |
| Mucinous | - | - | 2 | (2%) | - | - |  |
| Undifferentiated | 5 | (11%) | 6 | (6%) | 2 | (3%) |  |
| Mixed or other | 6 | (13%) | 3 | (3%) | 5 | (7%) |  |
| Missing info | - | - | 2 | (2%) | 1 | (1%) |  |
| **Grading** |  |  |  |  |  |  | 0.10 |
| G1 | 2 | (4%) | - | - | 5 | (7%) |  |
| G2 | 8 | (17%) | 14 | (13%) | 9 | (13%) |  |
| G3 | 30 | (64%) | 76 | (72%) | 48 | (68%) |  |
| Undifferentiated | 5 | (11%) | 6 | (6%) | 2 | (3%) |  |
| Missing info | 2 | (4%) | 9 | (9%) | 7 | (10%) |  |

## Figure S28. Progression-free survival

**
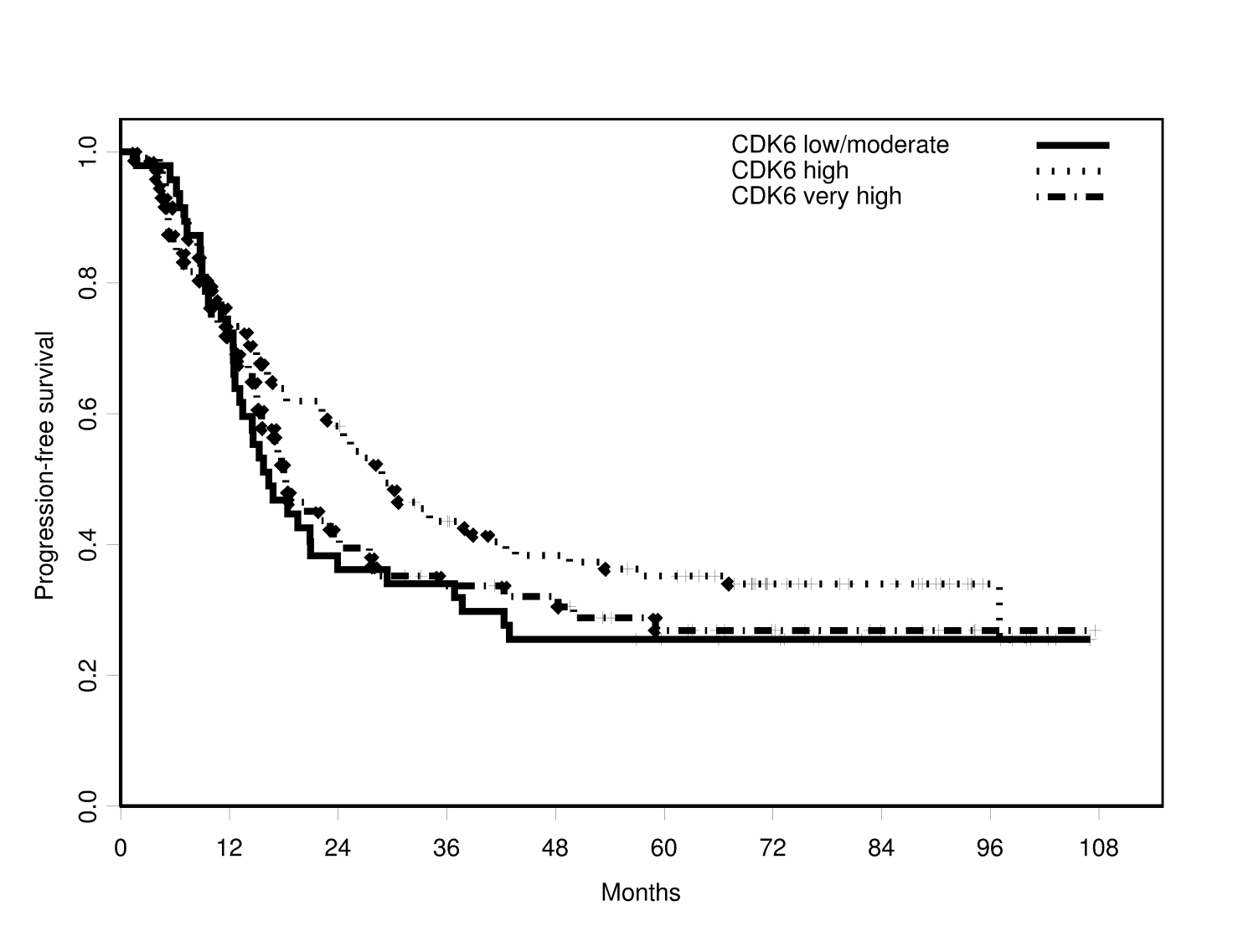
**

| **CDK6**  **intensity** | **N. pts** | **Events** | **Median PFS (months)** | **95%CI** |
| --- | --- | --- | --- | --- |
| Low/moderate | 47 | 35 | 16.3 | 13.2 - 36.9 |
| High | 105 | 69 | 29.0 | 23.1 - 43.6 |
| Very high | 71 | 51 | 18.2 | 15.6 - 27.7 |

## Figure S29. Overall survival

**
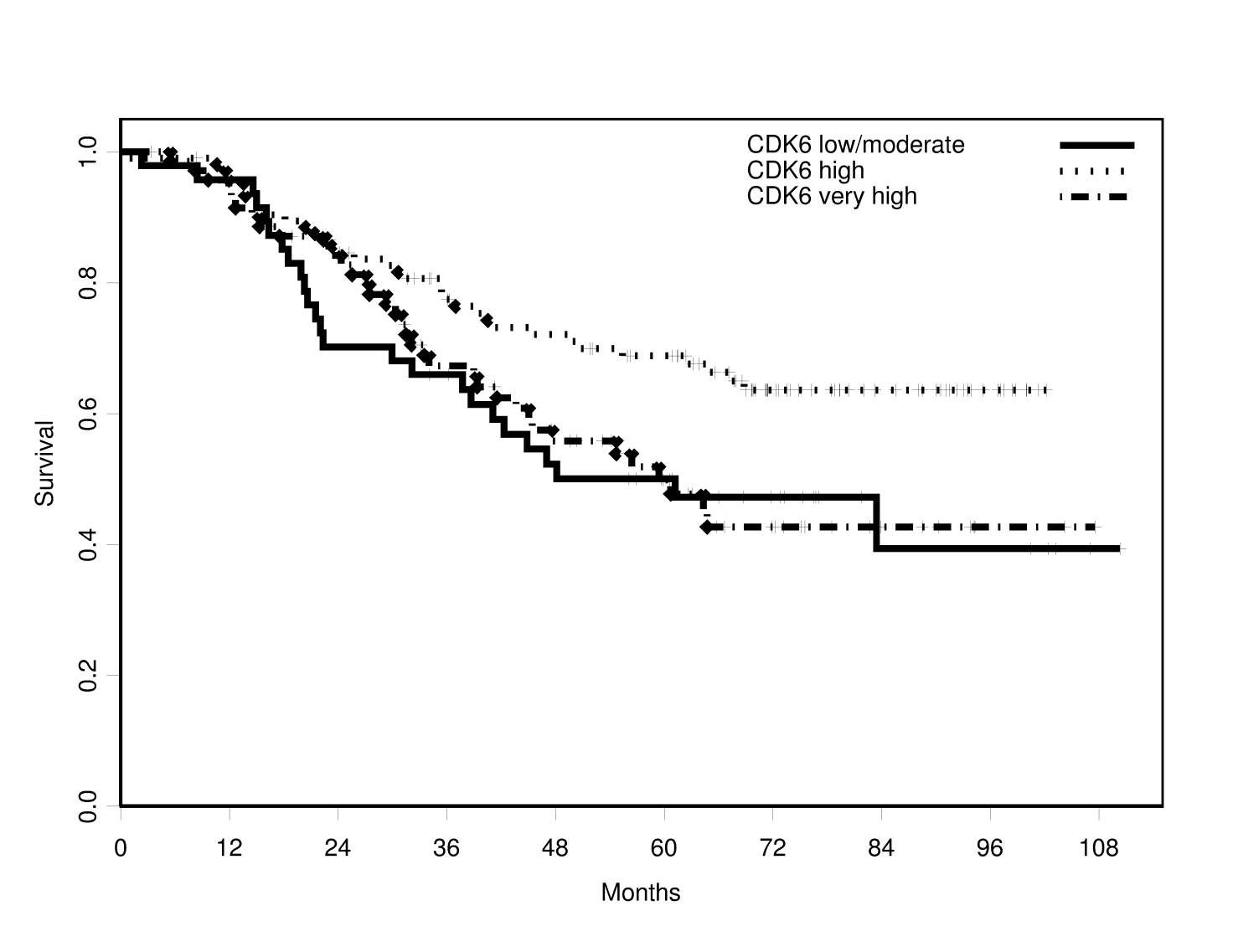
**

| **CDK6**  **intensity** | **N. pts** | **Events** | **Median OS**  **(months)** | **95%CI** |
| --- | --- | --- | --- | --- |
| Low/moderate | 47 | 25 | 61.2 | 38.7 - n.a. |
| High | 105 | 35 | n.a. | n.a. - n.a. |
| Very high | 71 | 35 | 59.4 | 45.1 - n.a. |

# CDK6 (localization)

## Table S18. Characteristics of patients according to CDK6 localization category

|  | **CDK6 cytoplasm**  **(n=156)** | | **CDK6 cytoplasm**  **membrane**  **(n=51)** | | **CDK6 cytoplasm**  **nucleus**  **(n=16)** | | **P value** |
| --- | --- | --- | --- | --- | --- | --- | --- |
| **Median age** (range) | 57 | (28-77) | 57 | (30-75) | 62 | (38-71) | 0.77 |
| Age < 70 yrs | 136 | (87%) | 47 | (92%) | 14 | (88%) | 0.63 |
| Age > 70 yrs | 20 | (13%) | 4 | (8%) | 2 | (12%) |  |
| **ECOG performance status** |  |  |  |  |  |  | 0.69 |
| 0-1 | 152 | (97%) | 49 | (96%) | 16 | (100%) |  |
| 2 | 4 | (3%) | 2 | (4%) | - |  |  |
| **Residual disease** |  |  |  |  |  |  | 0.27 |
| None | 58 | (37%) | 28 | (55%) | 7 | (44%) |  |
| ≤ 1 cm | 37 | (24%) | 8 | (16%) | 4 | (25%) |  |
| > 1 cm | 45 | (29%) | 13 | (25%) | 5 | (31%) |  |
| Not operated | 16 | (10%) | 2 | (4%) | - | - |  |
| **FIGO stage** |  |  |  |  |  |  | 0.26 |
| Ic | 10 | (6%) | 9 | (18%) | 1 | (6%) |  |
| II | 14 | (9%) | 3 | (6%) | 1 | (6%) |  |
| III | 110 | (71%) | 31 | (61%) | 13 | (81%) |  |
| IV | 22 | (14%) | 8 | (16%) | 1 | (6%) |  |
| **Tumor histology** |  |  |  |  |  |  | 0.57 |
| Serous | 110 | (71%) | 35 | (69%) | 12 | (75%) |  |
| Endometrioid | 20 | (13%) | 6 | (12%) | 1 | (6%) |  |
| Clear cell | 3 | (2%) | 3 | (6%) | 1 | (6%) |  |
| Mucinous | 1 | (1%) | 1 | (2%) | - | - |  |
| Undifferentiated | 12 | (8%) | 1 | (2%) | - | - |  |
| Mixed or other | 8 | (5%) | 4 | (8%) | 2 | (12%) |  |
| Missing info | 2 | (1%) | 1 | (2%) | - | - |  |
| **Grading** |  |  |  |  |  |  | 0.08 |
| G1 | 3 | (2%) | 4 | (8%) | - | - |  |
| G2 | 25 | (16%) | 5 | (10%) | 1 | (6%) |  |
| G3 | 103 | (66%) | 36 | (71%) | 15 | (94%) |  |
| Undifferentiated | 12 | (8%) | 1 | (2%) | - | - |  |
| Missing info | 13 | (8%) | 5 | (10%) | - | - |  |

## Figure S30. Progression-free survival

**
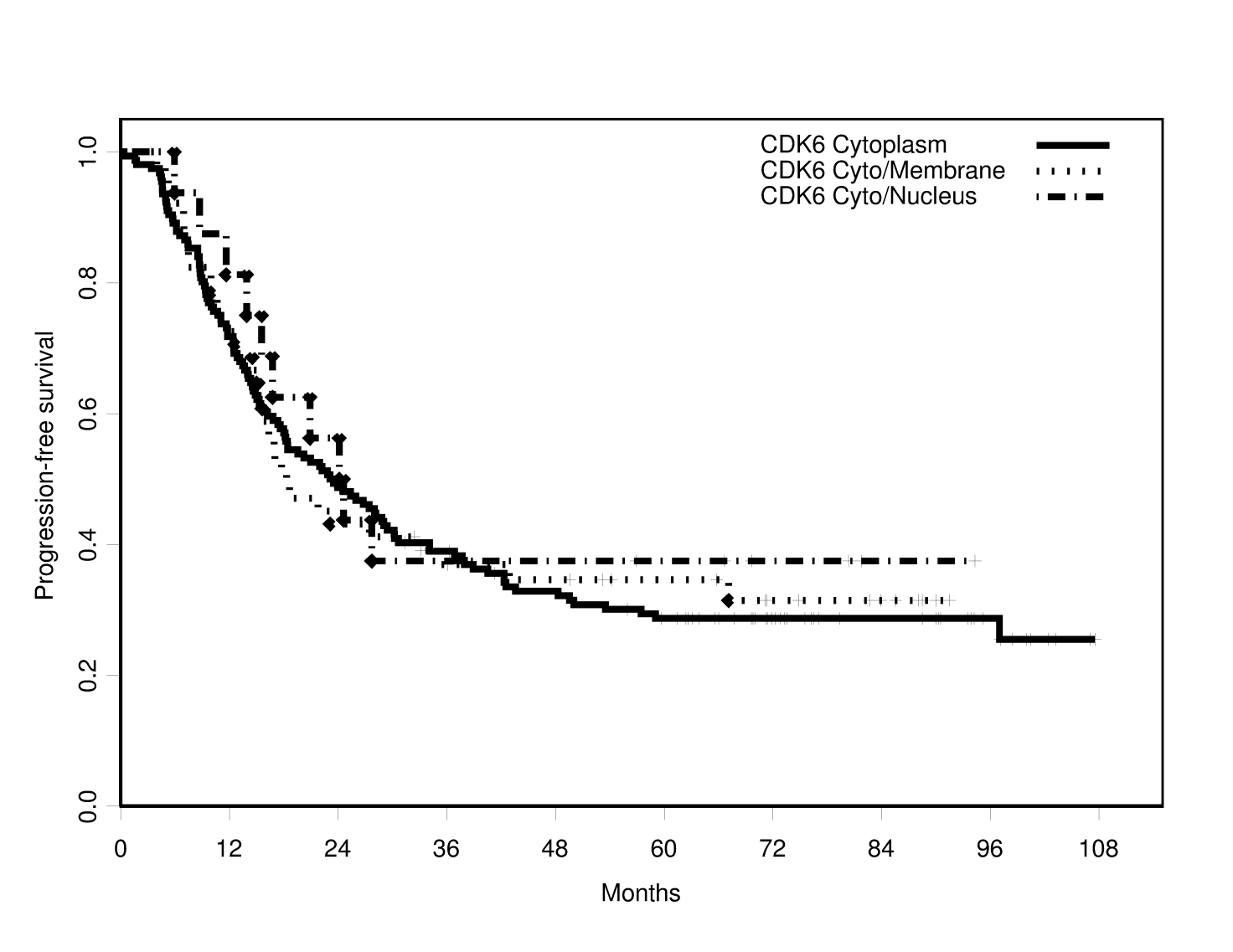
**

| **CDK6**  **localization** | **N. pts** | **Events** | **Median PFS (months)** | **95%CI** |
| --- | --- | --- | --- | --- |
| Cytoplasm | 156 | 111 | 23.5 | 18.0 - 30.2 |
| Cytoplasm / Membrane | 51 | 34 | 18.3 | 15.6 - 67.1 |
| Cytoplasm / Nucleus | 16 | 10 | 24.4 | 15.6 - n.a. |

## Figure S31. Overall survival

**
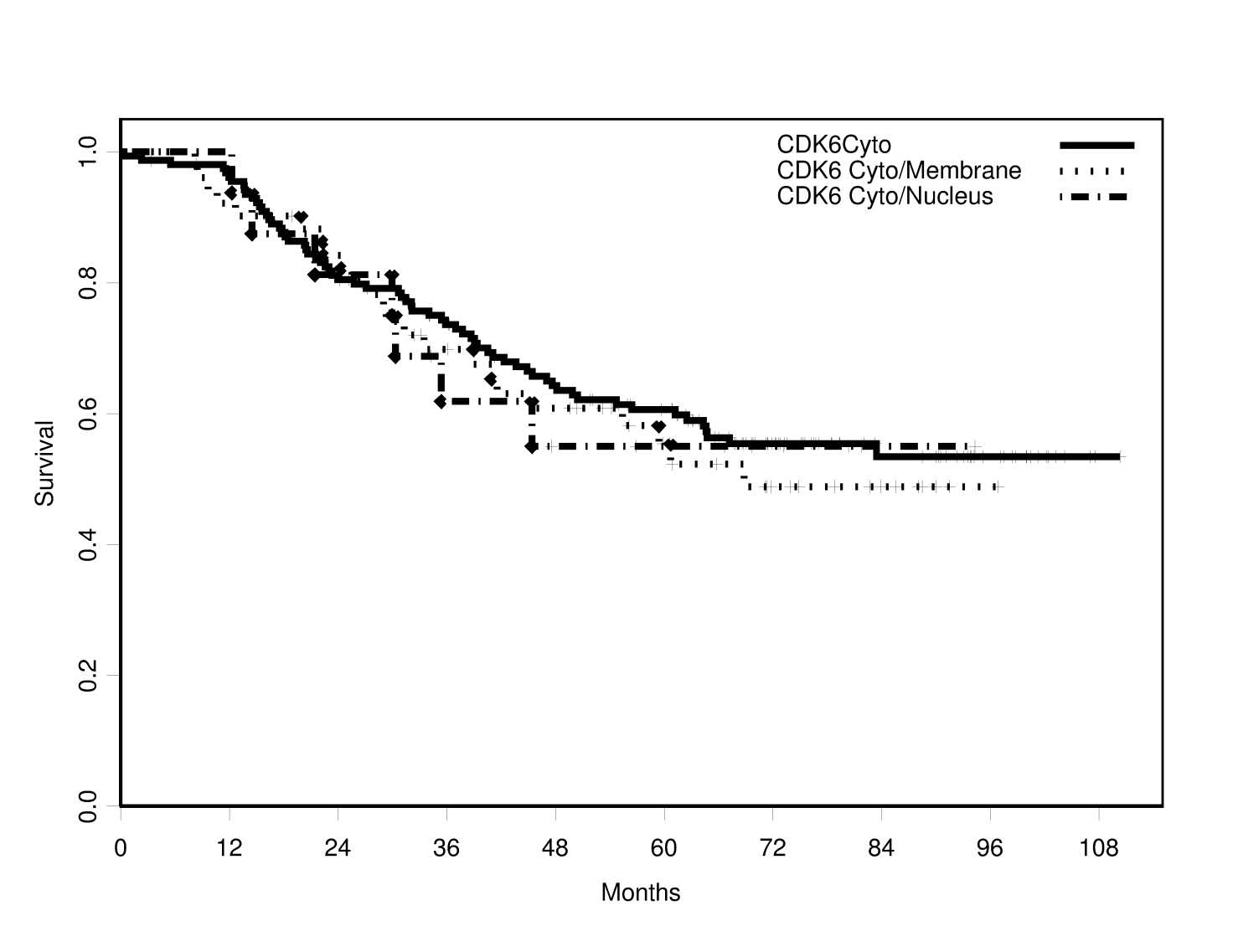
**

| **CDK6**  **localization** | **N. pts** | **Events** | **Median OS (months)** | **95%CI** |
| --- | --- | --- | --- | --- |
| Cytoplasm | 156 | 65 | n.a. | 64.6 - n.a. |
| Cytoplasm / Membrane | 51 | 23 | 68.8 | 45.1 - n.a. |
| Cytoplasm / Nucleus | 16 | 7 | n.a. | 30.3 - n.a. |

# Leptin receptor

## Table S19. Characteristics of patients according to Leptin receptor category

|  | **Leptin receptor**  **<10%**  **(n=78)** | | **Leptin receptor**  **10-50%**  **(n=35)** | | **Leptin receptor**  **>50%**  **(n=106)** | | **P value** |
| --- | --- | --- | --- | --- | --- | --- | --- |
| **Median age** (range) | 54 | (28-77) | 61 | (36-74) | 59 | (28-75) | 0.20 |
| Age < 70 yrs | 67 | (86%) | 32 | (91%) | 93 | (88%) | 0.71 |
| Age > 70 yrs | 11 | (14%) | 3 | (9%) | 13 | (12%) |  |
| **ECOG performance status** |  |  |  |  |  |  | 0.59 |
| 0-1 | 77 | (99%) | 34 | (97%) | 102 | (96%) |  |
| 2 | 1 | (1%) | 1 | (3%) | 4 | (4%) |  |
| **Residual disease** |  |  |  |  |  |  | 0.40 |
| None | 31 | (40%) | 17 | (49%) | 45 | (42%) |  |
| ≤ 1 cm | 20 | (26%) | 9 | (26%) | 19 | (18%) |  |
| > 1 cm | 22 | (28%) | 5 | (14%) | 35 | (33%) |  |
| Not operated | 5 | (6%) | 4 | (11%) | 7 | (7%) |  |
| **FIGO stage** |  |  |  |  |  |  | 0.67 |
| Ic | 8 | (10%) | 2 | (6%) | 10 | (9%) |  |
| II | 9 | (12%) | 2 | (6%) | 7 | (7%) |  |
| III | 51 | (65%) | 24 | (69%) | 77 | (73%) |  |
| IV | 10 | (13%) | 7 | (20%) | 12 | (11%) |  |
| **Tumor histology** |  |  |  |  |  |  |  |
| Serous | 53 | (68%) | 24 | (69%) | 76 | (72%) | 0.21 |
| Endometrioid | 11 | (14%) | 4 | (11%) | 13 | (12%) |  |
| Clear cell | 2 | (3%) | 1 | (3%) | 3 | (3%) |  |
| Mucinous | - |  | 2 | (6%) | - |  |  |
| Undifferentiated | 6 | (8%) | 3 | (9%) | 4 | (4%) |  |
| Mixed or other | 6 | (8%) | 1 | (3%) | 7 | (7%) |  |
| Missing info | - |  | - |  | 3 | (3%) |  |
| **Grading** |  |  |  |  |  |  |  |
| G1 | 2 | (3%) | 2 | (6%) | 3 | (3%) | 0.55 |
| G2 | 14 | (18%) | 6 | (17%) | 12 | (11%) |  |
| G3 | 53 | (68%) | 20 | (57%) | 78 | (74%) |  |
| Undifferentiated | 6 | (8%) | 3 | (9%) | 4 | (4%) |  |
| Missing info | 3 | (4%) | 4 | (11%) | 9 | (9%) |  |

## Figure S32. Progression-free survival


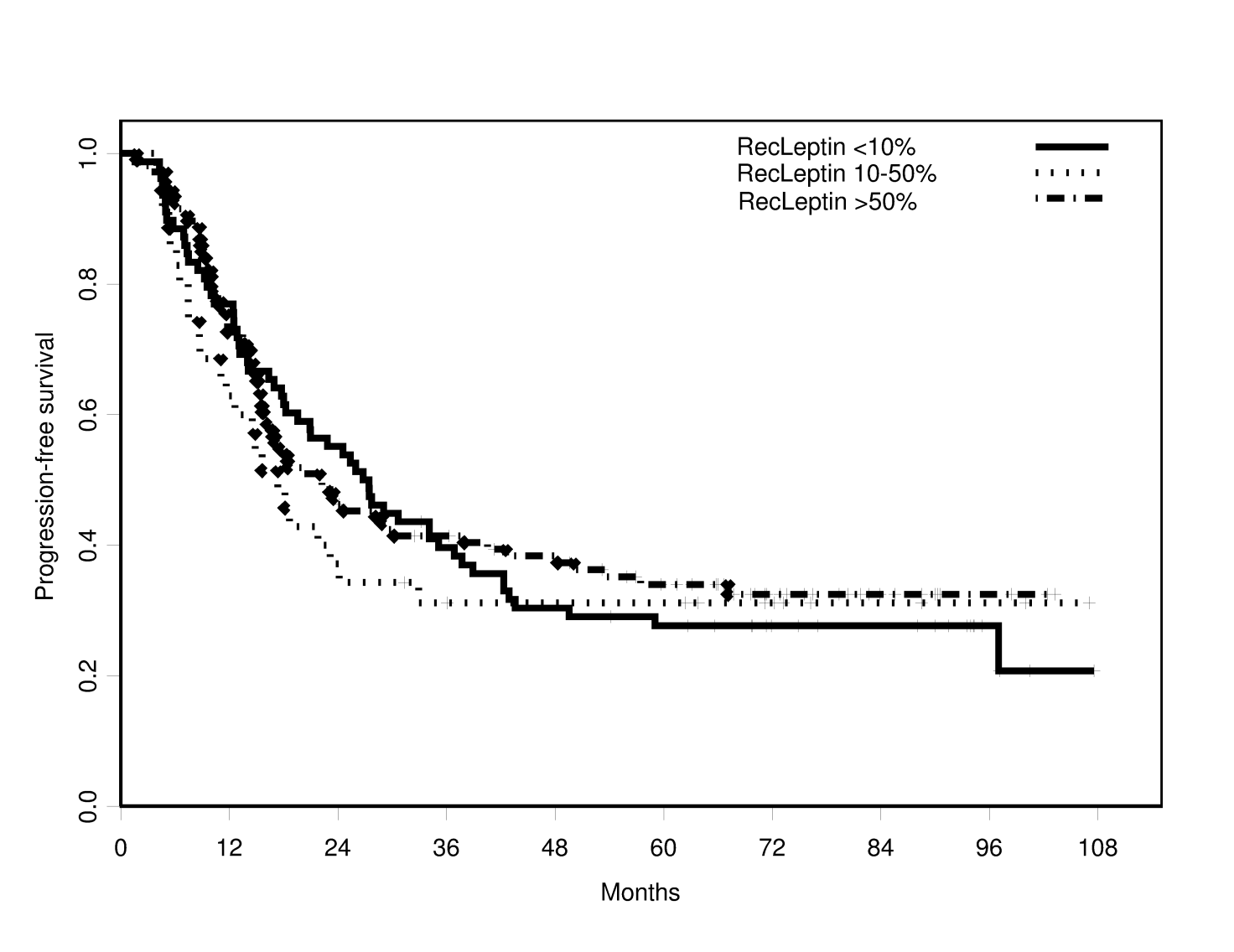


| **Leptin receptor**  **expression** | **N. pts** | **Events** | **Median PFS (months)** | **95%CI** |
| --- | --- | --- | --- | --- |
| <10% | 78 | 57 | 26.8 | 19.6 - 37.7 |
| 10-50% | 35 | 24 | 17.4 | 11.6 - n.a. |
| >50% | 106 | 70 | 22.2 | 16.2 - 40.5 |

## Figure S33. Overall survival

**
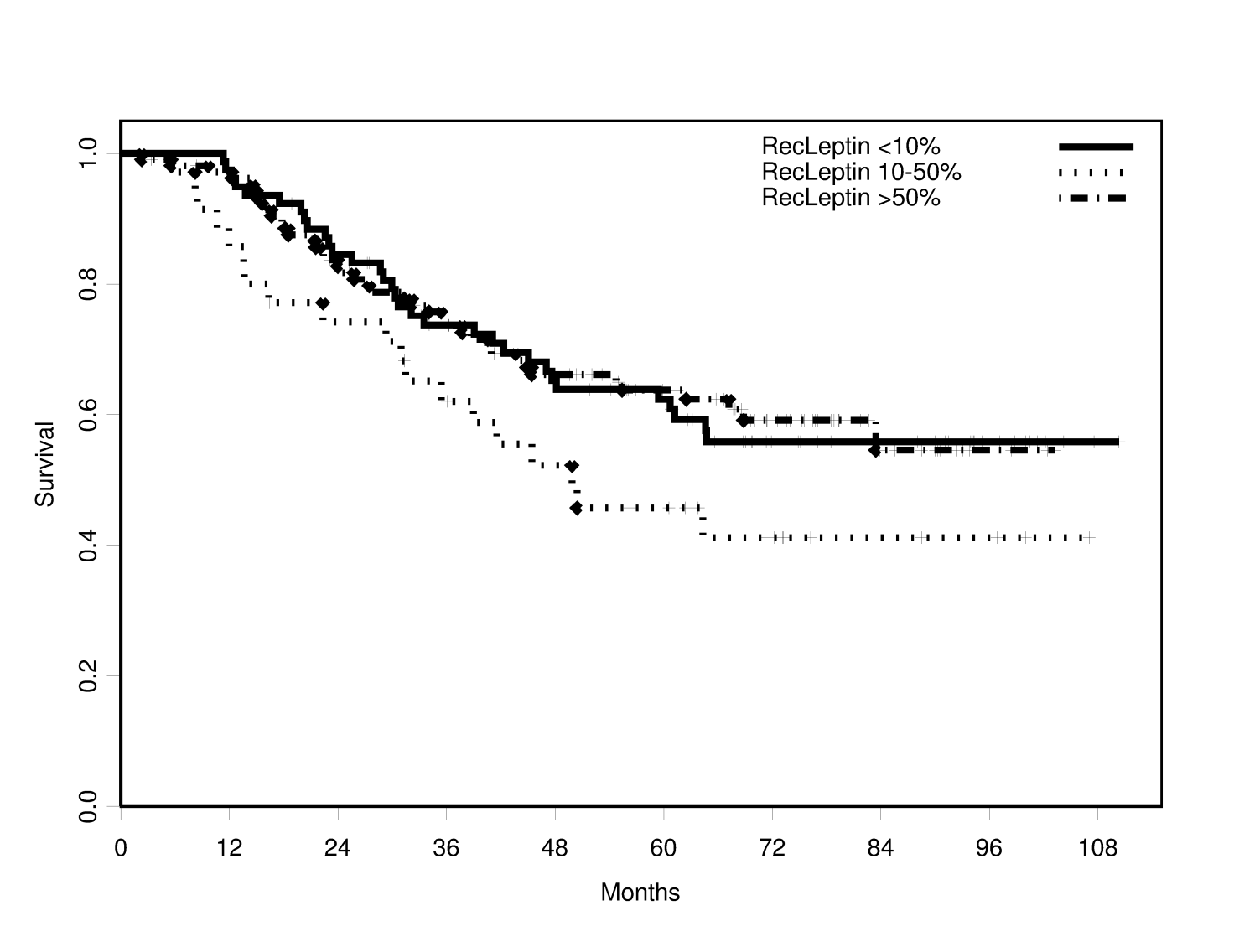
**

| **Leptin receptor expression** | **N. pts** | **Events** | **Median OS (months)** | **95%CI** |
| --- | --- | --- | --- | --- |
| <10% | 78 | 32 | n.a. | 61.2 - n.a. |
| 10-50% | 35 | 19 | 49.9 | 35.4 - n.a. |
| >50% | 106 | 40 | n.a. | 68.8 - n.a. |

# pAMPK

## Table S20. Characteristics of patients according to pAMPK category

|  | **pAMPK neg**  **(n=65)** | | **pAMPK pos**  **(n=88)** | | **P value** |
| --- | --- | --- | --- | --- | --- |
| **Median age** (range) | 54 | (28-76) | 58 | (30-77) | 0.04 |
| Age < 70 yrs | 58 | (89%) | 76 | (86%) | 0.60 |
| Age > 70 yrs | 7 | (11%) | 12 | (14%) |  |
| **ECOG performance status** |  |  |  |  | 0.19 |
| 0-1 | 64 | (98%) | 83 | (94%) |  |
| 2 | 1 | (2%) | 5 | (6%) |  |
| **Residual disease** |  |  |  |  | 0.08 |
| None | 28 | (43%) | 40 | (45%) |  |
| ≤ 1 cm | 20 | (31%) | 18 | (20%) |  |
| > 1 cm | 16 | (25%) | 20 | (23%) |  |
| Not operated | 1 | (2%) | 10 | (11%) |  |
| **FIGO stage** |  |  |  |  | 0.19 |
| Ic | 5 | (8%) | 11 | (12%) |  |
| II | 3 | (5%) | 10 | (11%) |  |
| III | 45 | (69%) | 58 | (66%) |  |
| IV | 12 | (18%) | 9 | (10%) |  |
| **Tumor histology** |  |  |  |  | 0.14 |
| Serous | 46 | (71%) | 61 | (69%) |  |
| Endometrioid | 5 | (8%) | 15 | (17%) |  |
| Clear cell | 1 | (2%) | 2 | (2%) |  |
| Mucinous | - |  | 1 | (1%) |  |
| Undifferentiated | 5 | (8%) | 4 | (5%) |  |
| Mixed or other | 8 | (12%) | 3 | (3%) |  |
| Missing information | - |  | 2 | (2%) |  |
| **Grading** |  |  |  |  | 0.31 |
| G1 | - |  | 4 | (5%) |  |
| G2 | 9 | (14%) | 15 | (17%) |  |
| G3 | 48 | (74%) | 58 | (66%) |  |
| Undifferentiated | 5 | (8%) | 4 | (5%) |  |
| Missing information | 3 | (5%) | 7 | (8%) |  |

## Figure S34. Progression-free survival

**
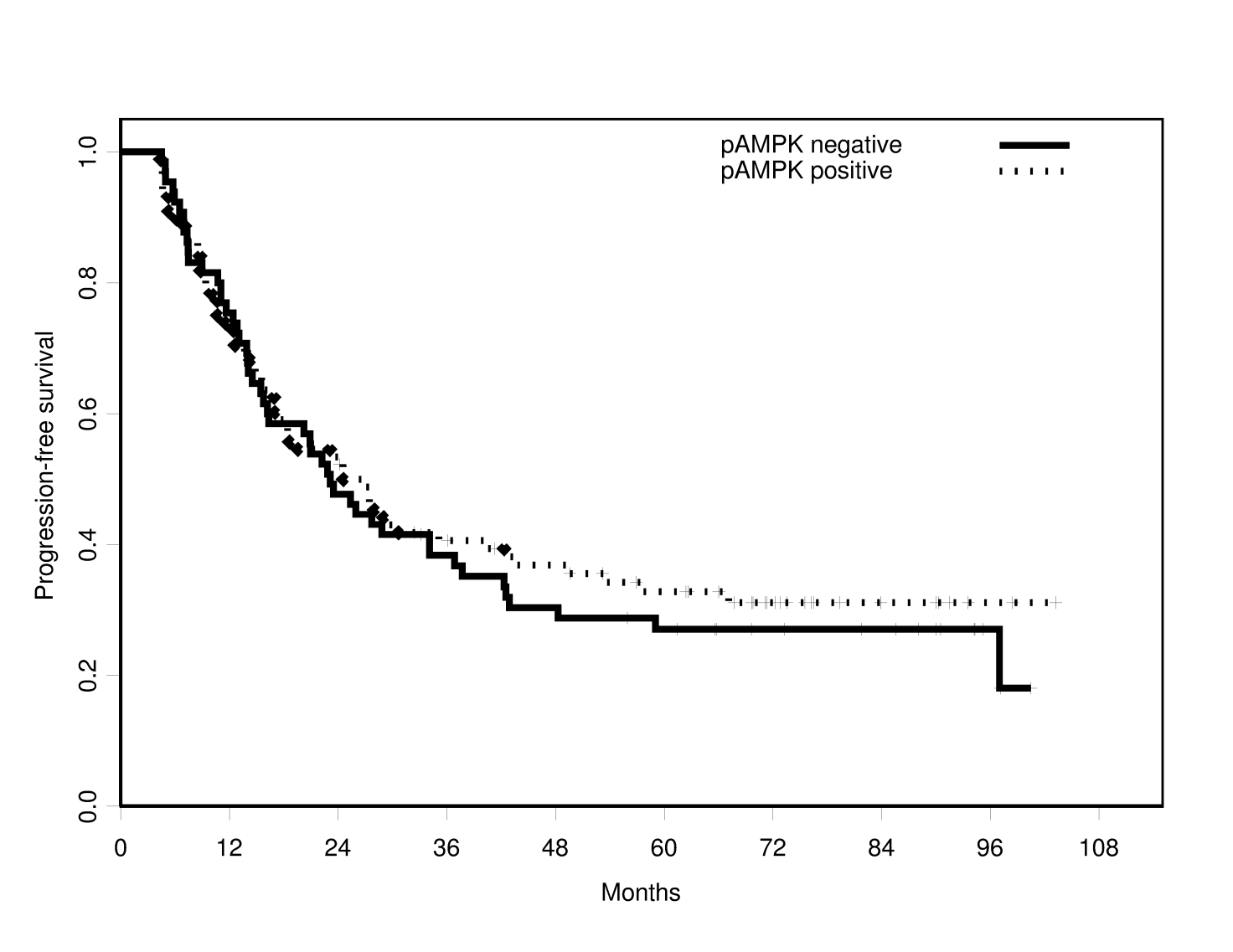
**

| **pAMPK expression** | **N. pts** | **Events** | **Median PFS (months)** | **95%CI** |
| --- | --- | --- | --- | --- |
| Negative | 65 | 48 | 23.1 | 16.2 - 37-7 |
| Positive | 88 | 59 | 24.6 | 17.4 - 42.3 |

## Figure S35. Overall survival

**
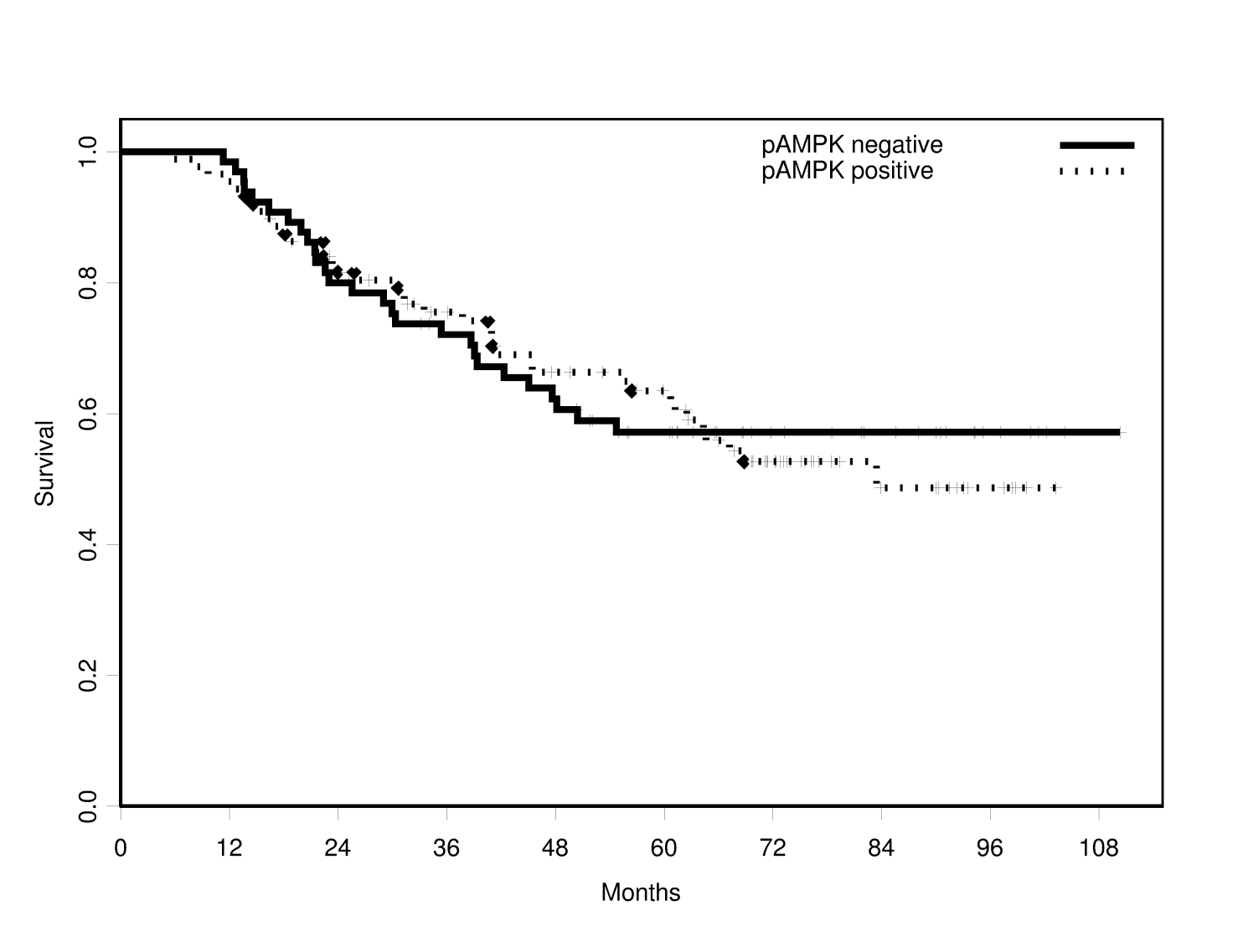
**

| **pAMPK expression** | **N. pts** | **Events** | **Median OS (months)** | **95%CI** |
| --- | --- | --- | --- | --- |
| Negative | 65 | 27 | n.a. | 48.1 - n.a. |
| Positive | 88 | 38 | 83.4 | 62.5 - n.a. |

# pACC

## Table S21. Characteristics of patients according to pACC category

|  | **pACC neg**  **(n=78)** | | **pACC pos**  **(n=92)** | | **P value** |
| --- | --- | --- | --- | --- | --- |
| **Median age** (range) | 55 | (28-74) | 58 | (30-77) | 0.02 |
| Age < 70 yrs | 73 | (94%) | 78 | (85%) | 0.07 |
| Age > 70 yrs | 5 | (6%) | 14 | (15%) |  |
| **ECOG performance status** |  |  |  |  | 0.15 |
| 0-1 | 77 | (99%) | 87 | (95%) |  |
| 2 | 1 | (1%) | 5 | (5%) |  |
| **Residual disease** |  |  |  |  | 0.08 |
| None | 32 | (41%) | 32 | (35%) |  |
| ≤ 1 cm | 24 | (31%) | 21 | (23%) |  |
| > 1 cm | 20 | (26%) | 28 | (30%) |  |
| Not operated | 2 | (3%) | 11 | (12%) |  |
| **FIGO stage** |  |  |  |  | 0.51 |
| Ic | 7 | (9%) | 8 | (9%) |  |
| II | 5 | (6%) | 3 | (3%) |  |
| III | 57 | (73%) | 64 | (70%) |  |
| IV | 9 | (12%) | 17 | (18%) |  |
| **Tumor histology** |  |  |  |  | 0.88 |
| Serous | 59 | (76%) | 65 | (71%) |  |
| Endometrioid | 6 | (8%) | 4 | (4%) |  |
| Clear cell | 2 | (3%) | 7 | (8%) |  |
| Mucinous | 1 | (1%) | 1 | (1%) |  |
| Undifferentiated | 4 | (5%) | 5 | (5%) |  |
| Mixed or other | 6 | (8%) | 4 | (4%) |  |
| Missing information | - | - | 3 | (3%) |  |
| **Grading** |  |  |  |  | 0.94 |
| G1 | 3 | (4%) | 2 | (2%) |  |
| G2 | 14 | (18%) | 14 | (15%) |  |
| G3 | 55 | (71%) | 61 | (66%) |  |
| Undifferentiated | 4 | (5%) | 5 | (5%) |  |
| Missing information | 2 | (3%) | 10 | (11%) |  |

## Figure S36. Progression-free survival

**
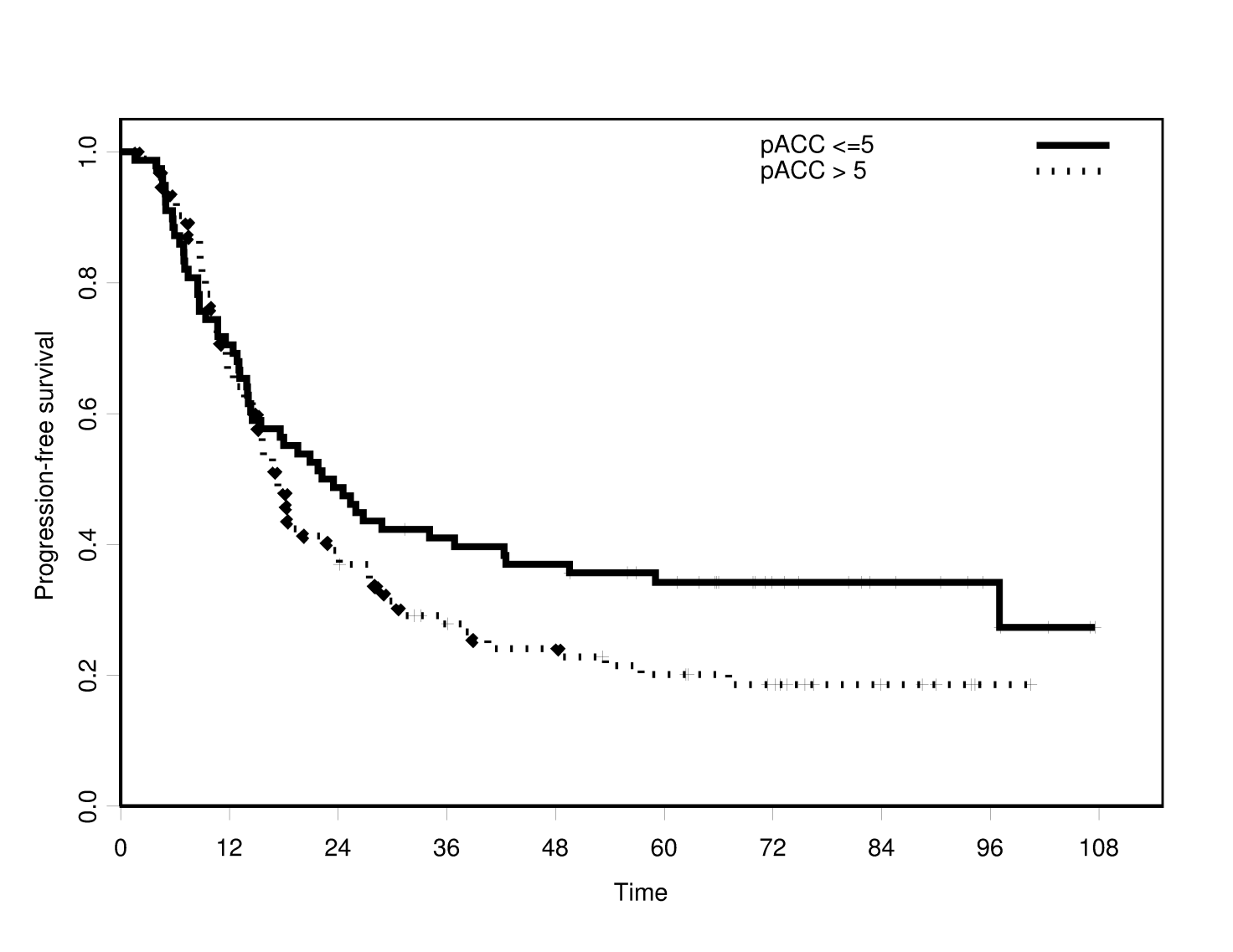
**

| **pACC expression** | **N. pts** | **Events** | **Median PFS (months)** | **95%CI** |
| --- | --- | --- | --- | --- |
| Negative | 78 | 52 | 22.2 | 14.5 - 42.5 |
| Positive | 92 | 73 | 17.0 | 15.0 - 23.9 |

## Figure S37. Overall survival

**
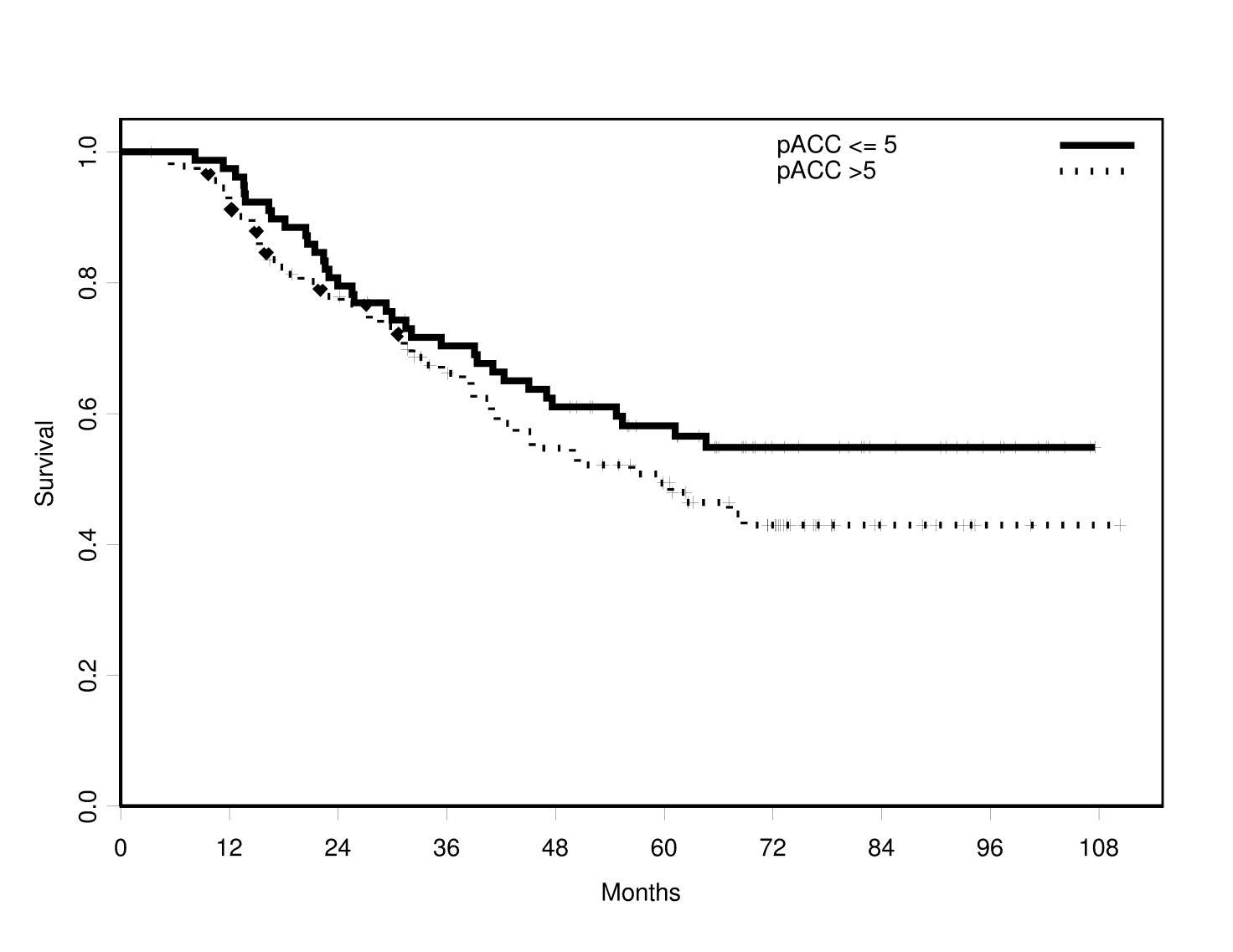
**

| **pACC expression** | **N. pts** | **Events** | **Median OS (months)** | **95%CI** |
| --- | --- | --- | --- | --- |
| Negative | 78 | 34 | n.a. | 54.7 - n.a. |
| Positive | 92 | 47 | 59.4 | 41.5 - n.a. |

# Stathmin

## Table S22. Characteristics of patients according to Stathmin category

|  | **Stathmin**  **Negative/ moderate**  **(n=150)** | | **Stathmin high**  **intensity**  **(n=64)** | | **P value** |
| --- | --- | --- | --- | --- | --- |
| **Median age** (range) | 57 | (28-76) | 58 | (37-77) | 0.12 |
| Age < 70 yrs | 136 | (91%) | 51 | (80%) | 0.03 |
| Age > 70 yrs | 14 | (9%) | 13 | (20%) |  |
| **ECOG performance status** |  |  |  |  | 0.85 |
| 0-1 | 146 | (97%) | 62 | (97%) |  |
| 2 | 4 | (3%) | 2 | (3%) |  |
| **Residual disease** |  |  |  |  | 0.89 |
| None | 59 | (39%) | 28 | (44%) |  |
| ≤ 1 cm | 34 | (23%) | 15 | (23%) |  |
| > 1 cm | 45 | (30%) | 16 | (25%) |  |
| Not operated | 12 | (8%) | 5 | (8%) |  |
| **FIGO stage** |  |  |  |  | 0.57 |
| Ic | 15 | (10%) | 3 | (5%) |  |
| II | 12 | (8%) | 6 | (9%) |  |
| III | 101 | (67%) | 47 | (73%) |  |
| IV | 22 | (15%) | 8 | (12%) |  |
| **Tumor histology** |  |  |  |  | 0.19 |
| Serous | 106 | (71%) | 44 | (69%) |  |
| Endometrioid | 15 | (10%) | 12 | (19%) |  |
| Clear cell | 6 | (4%) | - | - |  |
| Mucinous | 2 | (1%) | - | - |  |
| Undifferentiated | 9 | (6%) | 3 | (5%) |  |
| Mixed or other | 11 | (7%) | 3 | (5%) |  |
| Missing info | 1 | (7%) | 2 | (3%) |  |
| **Grading** |  |  |  |  | 0.79 |
| G1 | 5 | (3%) | 1 | (2%) |  |
| G2 | 23 | (15%) | 7 | (11%) |  |
| G3 | 102 | (68%) | 47 | (73%) |  |
| Undifferentiated | 9 | (6%) | 3 | (5%) |  |
| Missing info | 11 | (7%) | 6 | (9%) |  |

## Figure S38. Progression-free survival

**
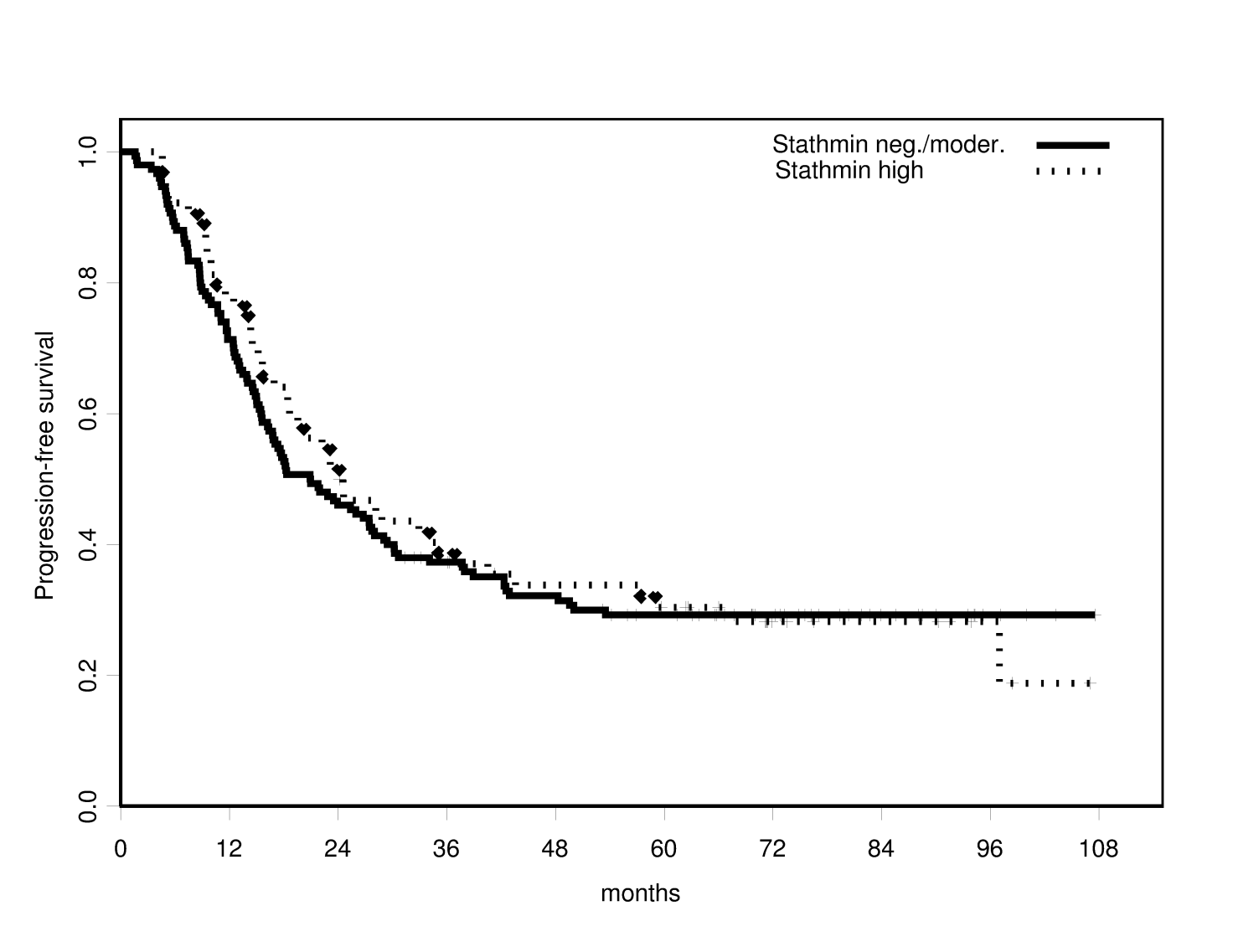
**

| **Stathmin**  **intensity** | **N. pts** | **Events** | **Median PFS (months)** | **95%CI** |
| --- | --- | --- | --- | --- |
| Negative / Moderate | 150 | 105 | 21.0 | 16.3 - 29.0 |
| High | 64 | 46 | 24.3 | 18.7 - 40.5 |

## Figure S39. Overall survival

**
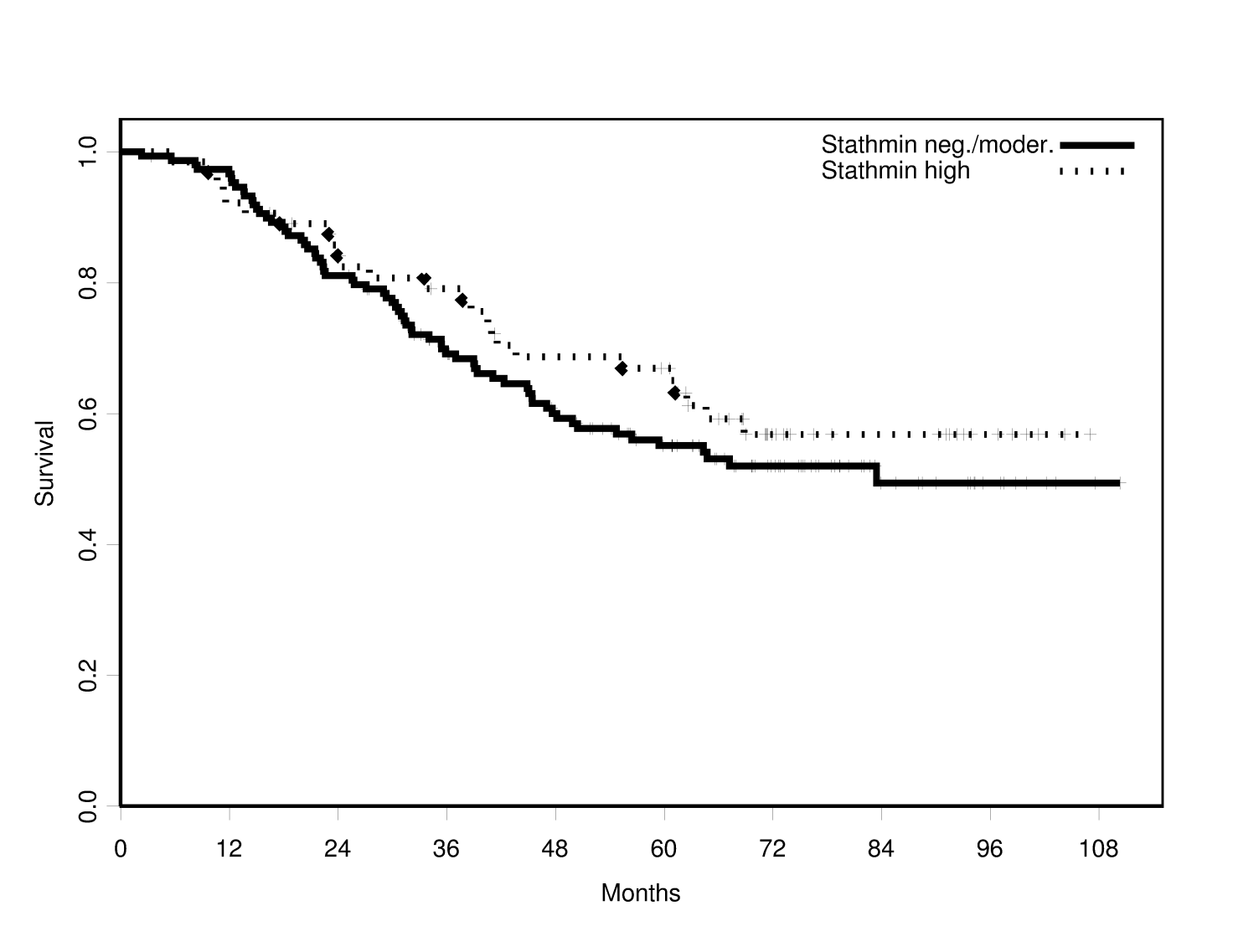
**

| **Stathmin**  **intensity** | **N. pts** | **Events** | **Median OS (months)** | **95%CI** |
| --- | --- | --- | --- | --- |
| Negative /Moderate | 150 | 67 | 83.4 | 54.7 - n.a. |
| High | 64 | 25 | n.a. | 62.5 - n.a. |

# DNA-PK

## Table S23. Characteristics of patients according to DNA-PK category

|  | **DNA-PK**  **Negative/moderate**  **(n=131)** | | **DNA-PK high**  **intensity**  **(n=85)** | | **P value** |
| --- | --- | --- | --- | --- | --- |
| **Median age** (range) | 56 | (28-75) | 58 | (34-77) | 0.006 |
| Age < 70 yrs | 120 | (92%) | 70 | (82%) | 0.04 |
| Age > 70 yrs | 11 | (8%) | 15 | (18%) |  |
| **ECOG performance status** |  |  |  |  | 0.59 |
| 0-1 | 128 | (98%) | 82 | (96%) |  |
| 2 | 3 | (2%) | 3 | (4%) |  |
| **Residual disease** |  |  |  |  | 0.001 |
| None | 66 | (50%) | 24 | (28%) |  |
| ≤ 1 cm | 31 | (24%) | 17 | (20%) |  |
| > 1 cm | 27 | (21%) | 35 | (41%) |  |
| Not operated | 7 | (5%) | 9 | (11%) |  |
| **FIGO stage** |  |  |  |  | 0.29 |
| Ic | 15 | (11%) | 4 | (5%) |  |
| II | 12 | (9%) | 6 | (7%) |  |
| III | 88 | (67%) | 61 | (72%) |  |
| IV | 16 | (12%) | 14 | (16%) |  |
| **Tumor histology** |  |  |  |  | 0.051 |
| Serous | 90 | (69%) | 63 | (74%) |  |
| Endometrioid | 13 | (10%) | 14 | (16%) |  |
| Clear cell | 5 | (4%) | 2 | (2%) |  |
| Mucinous | 2 | (2%) | - | - |  |
| Undifferentiated | 12 | (9%) | - | - |  |
| Mixed or other | 8 | (6%) | 4 | (5%) |  |
| Missing info | 1 | (1%) | 2 | (2%) |  |
| **Grading** |  |  |  |  | 0.02 |
| G1 | 5 | (4%) | 2 | (2%) |  |
| G2 | 19 | (15%) | 11 | (13%) |  |
| G3 | 89 | (68%) | 62 | (73%) |  |
| Undifferentiated | 12 | (9%) | - | - |  |
| Missing info | 6 | (5%) | 10 | (12%) |  |

## Figure S40. Progression-free survival

**
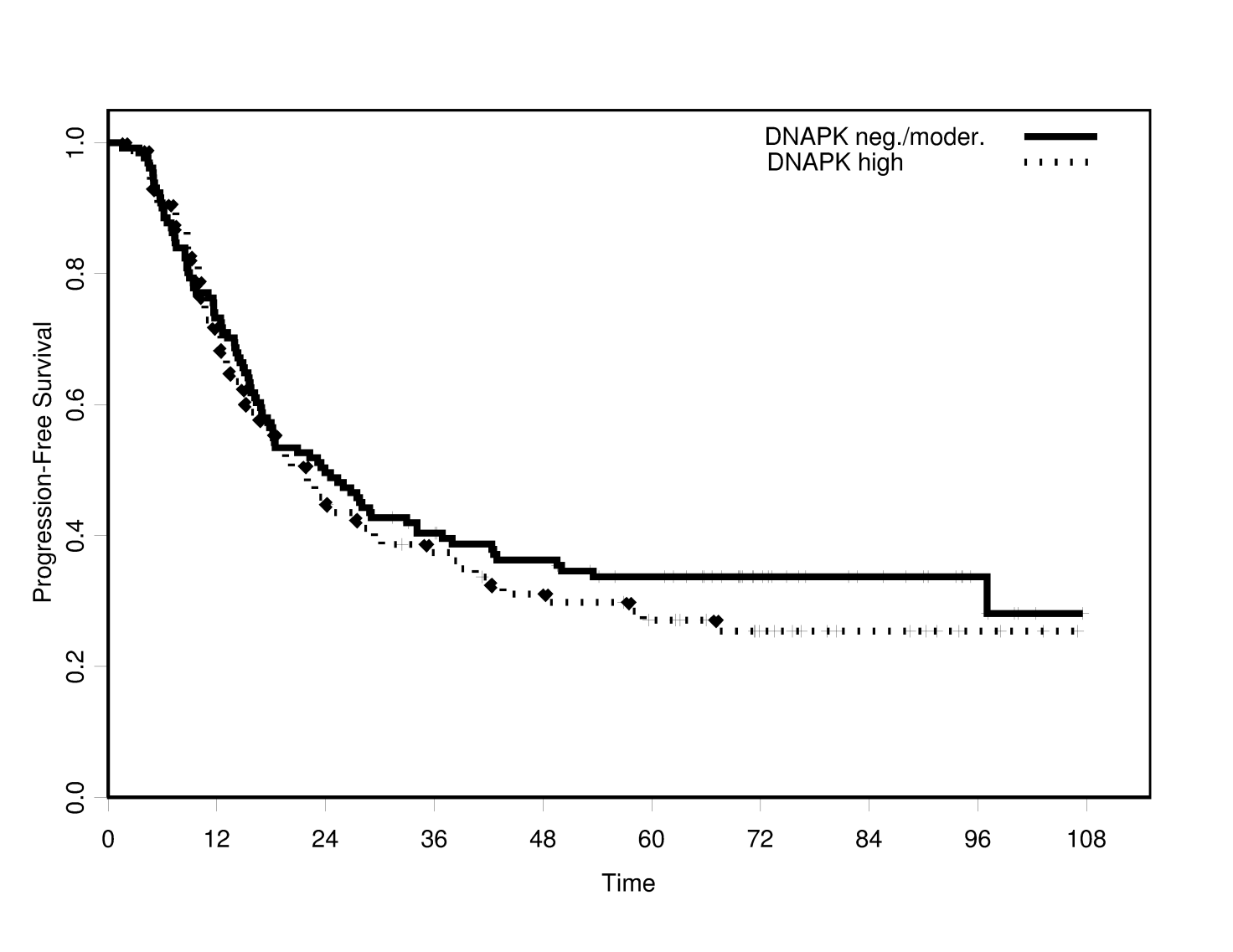
**

| **DNA-PK intensity** | **N. pts** | **Events** | **Median PFS (months)** | **95%CI** |
| --- | --- | --- | --- | --- |
| Negative / moderate | 131 | 87 | 23.9 | 17.6 - 34.1 |
| High | 85 | 62 | 21.8 | 15.6 - 35.1 |

## Figure S41. Overall survival

**
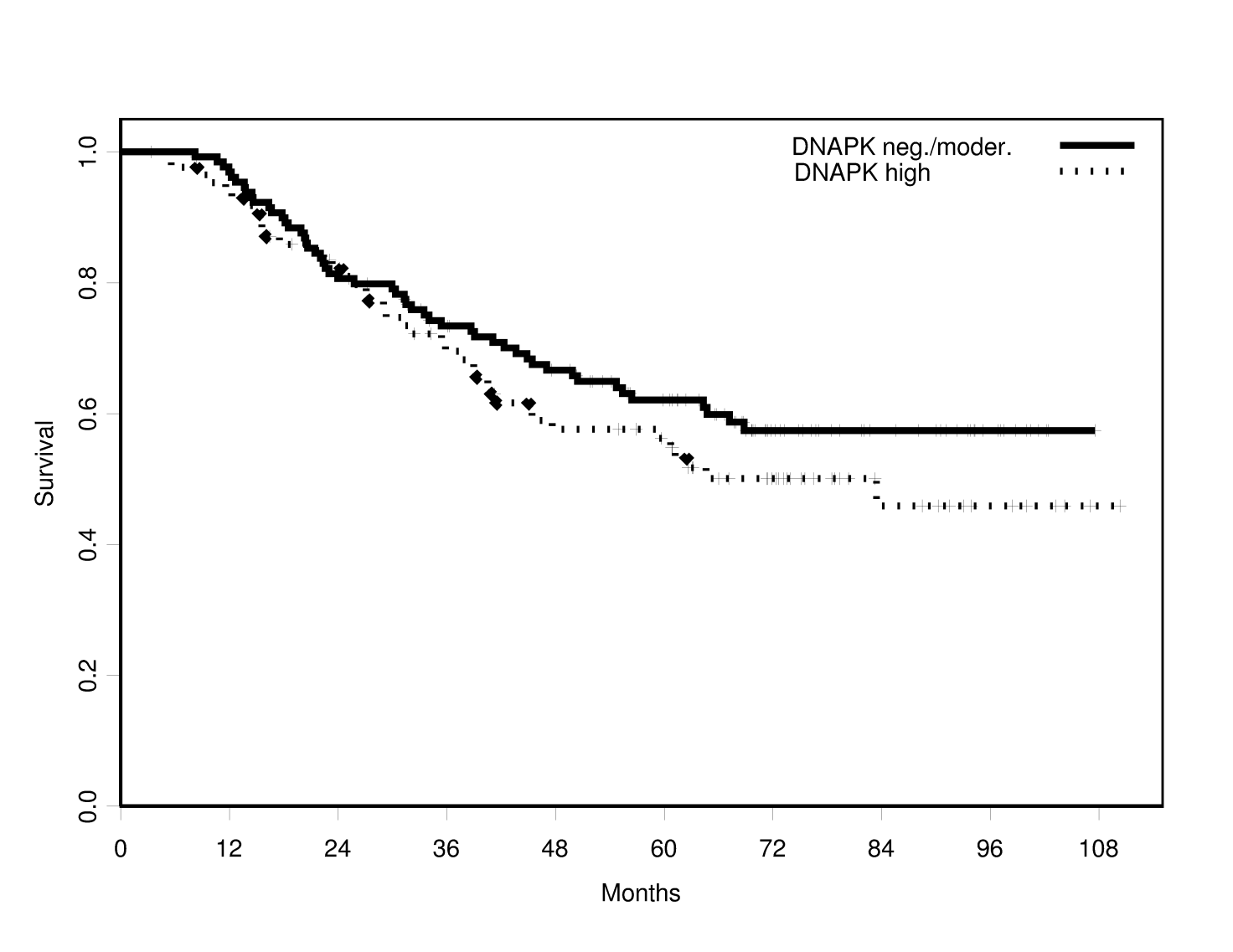
**

| **DNA-PK intensity** | **N. pts** | **Events** | **Median OS (months)** | **95%CI** |
| --- | --- | --- | --- | --- |
| Negative / moderate | 131 | 51 | n.a. | 68.8 - n.a. |
| High | 85 | 40 | 83.4 | 45.4 - n.a. |

#

# Investigators who participated in MITO2

## Writing committee (alphabetical order)

Gustavo Baldassarre, Daniela Califano, Silvana Canevari, Massimo Di Maio, Gabriella Ferrandina, Ciro Gallo, Stefano Indraccolo, Domenica Lorusso, Francesco Perrone, Sandro Pignata.

## Centralization, preparation and validation of TMA

Simona Losito, Renato Franco, Giosuè Scognamiglio, Concetta Aiello, Giuliana Canzanella (Istituto Nazionale per lo Studio e la Cura dei Tumori “Fondazione Giovanni Pascale”, IRCCS, Napoli).

## Analysis of ALCAM, MCAM, CAV1, cFLIP

Delia Mezzanzanica, Marina Bagnoli, Silvana Canevari, Ludmila Liliac (Fondazione IRCCS Istituto Nazionale dei Tumori, Dept Experimental Oncology and Molecular Medicine, Unit of Molecular Therapies, Milano).

## Analysis of Stathmin, DNA-PK, CDK6

Gustavo Baldassarre, Vincenzo Canzonieri, Monica Schiappacassi (CRO, National Cancer Institute, Aviano [PN]).

## Analysis of pAMPK, pACC

Stefano Indraccolo, Elisabetta Zulato, Giovanni Esposito (U.O.C. Immunologia e Diagnostica Molecolare Oncologica - Istituto Oncologico Veneto - IRCCS, Padova).

## Analysis of p53

Gabriella Ferrandina, Enrica Martinelli (Laboratory of Antineoplastic Pharmacology

Department of Gynecology and Obstetrics, Catholic University, Rome), Gian Franco Zannoni (Pathology, Catholic University, Rome).

## Analysis of TRAP1

Franca Esposito, Diana Arzeni (Dipartimento di Medicina Molecolare e Biotecnologie Mediche, Università di Napoli Federico II, Napoli).

## Analysis of HMGA2, BAG3

Gennaro Chiappetta, Daniela Califano, Francesca Galdiero, Daniela Russo (Istituto Nazionale per lo Studio e la Cura dei Tumori “Fondazione Giovanni Pascale”, IRCCS, Napoli).

## Analysis of Claudin3, Leptin receptor, HOXB13

Simona Losito, Renato Franco, Giosuè Scognamiglio (Istituto Nazionale per lo Studio e la Cura dei Tumori “Fondazione Giovanni Pascale”, IRCCS, Napoli).

## Statistical analysis

Ciro Gallo, Simona Signoriello, Paolo Chiodini, Giuseppe Signoriello (Cattedra di Statistica Medica, Seconda Università, Napoli); Massimo Di Maio (Unità Sperimentazioni Cliniche, Istituto Nazionale per lo Studio e la Cura dei Tumori “Fondazione Giovanni Pascale”, IRCCS, Napoli, present address: Università di Torino).

## Pathologists

Simona Losito, Renato Franco, Giosuè Scognamiglio, Concetta Aiello, (Istituto Nazionale per lo Studio e la Cura dei Tumori “Fondazione Giovanni Pascale”, IRCCS, Napoli); Vincenzo Canzonieri (CRO, National Cancer Institute, Aviano [PN]); Gian Franco Zannoni (Pathology, Catholic University, Rome); Maria Antonia Carosi (Istituto Nazionale Tumori Regina Elena, Roma); Elena Cicerone (Ospedale S. Giovanni Calibita Fatebenefratelli, Roma); Mattia Barbareschi (Ospedale S. Chiara, Trento); Gianni Raulli (Ospedale S.Maria delle Croci, Ravenna); Giulia Sindici (Anatomia Patologica Ospedale Civile Spirito Santo, Pescara); Ilaria Franceschetti (Ospedale S. Bortolo ULSS 6, Vicenza).

## Clinical Investigators

Sandro Pignata, Carmela Pisano, Gaetano Facchini, Giovanni Salvatore Bruni, Lucia Cannella, Davide Leopardo, Stefano Greggi, Francesco Iodice (deceased), Gennaro Casella (Dipartimento Uro-Ginecologico, Istituto Nazionale per lo Studio e la Cura dei Tumori “Fondazione Giovanni Pascale”-IRCCS, Napoli); Giovanni Scambia, Domenica Lorusso, Vanda Salutari, Antonia Testa, Rosa De Vincenzo, Alessia di Legge, Claudia Masi, Valeria Masciullo, Mirella Di Stefano (Policlinico Universitario Gemelli, Università Cattolica del Sacro Cuore, Roma); Gabriella Ferrandina, Francesco Legge, Giacomo Corrado, Marco Petrillo, Aida Distefano, Manuela Ludovisi (Centro di Ricerca e Formazione ad Alta Tecnologia nelle Scienze Biomediche, Università Cattolica del Sacro Cuore, Campobasso); Antonella Savarese, Francesco Cognetti, Maria Antonia Carosi, Gianluigi Ferretti, Enrico Vizza, Emanuela Mancini (Istituto Nazionale Tumori Regina Elena, Roma); Roberto Sorio, Simona Scalone, Giorgio Giorda, Elio Campagnutta (Centro di Riferimento Oncologico, Aviano [PN]); Enrico Breda, Antonella Mecozzi, Emanuela Proietti, Loredana Rossi, Angelo Fedele Scinto (Ospedale S. Giovanni Calibita Fatebenefratelli, Roma); Vittorio Gebbia, Eugenia Baiardi, Carlo Arcara, Antonio Testa (Casa di Cura La Maddalena, Palermo); Pietro Musso, Rosa Demma, Francesco Maria Re (A.R.N.A.S. Civico Di Cristina Benfratelli, Palermo); Luigi Frigerio, Luisa Busci, Antonella Villa (present address: Clinica Mangiagalli, Milano), Laura Carlini (present address: Ospedale Bolognini, Seriate [BG]; (Ospedali Riuniti, Bergamo); Pietro Del Medico, Mario Nardi, Mario Raffaele, Domenico Azzarello (Ospedale Bianchi Melacrino Morelli, Reggio Calabria); Alessandra Vernaglia Lombardi, Carmine Malzoni, Mario Malzoni, Giuseppina Farnetano (Casa di Cura Malzoni Villa dei Platani, Avellino); Antonio Febbraro, Claudia Corbo, Ilaria Spagnoletti (Ospedale Fatebenefratelli, Benevento); Paolo Scollo, Giuseppe Scibilia, Gabriella D'Agate (Ospedale Cannizzaro**,** Catania); Enzo Galligioni, Antonella Ferro, Viviana Murgia, (Ospedale S. Chiara, Trento); Stefano Tamberi, Laura Amaducci, Carmelo Bucolo, (Ospedale Civile, Faenza); Alba Brandes, Simonetta Rimondini, Stefania Bartolini (Ospedale Bellaria, Bologna); Alberto Ravaioli, Giovanni Oliverio, Enzo Pasquini (Ospedale degli Infermi, Rimini, Ospedale Cervesi, Cattolica [RN]); Maria Rosaria Valerio, Mario Lomauro, Nicola Gebbia (Policlinico Giaccone, Università, Palermo); Enrico Aitini, Giovanna Cavazzini, Francesca Adami (Ospedale Carlo Poma, Mantova); Donato Natale, Bruna Fornarini, Dante Orlando (Osp. S. Massimo**,** Penne [PE]); Fabrizio Artioli, Laura Scaltriti, Lorenzo Aguzzoli (Ospedale Ramazzini, Carpi [MO]); Emanuele Naglieri, Agnese Latorre, Sabina Delcuratolo (Istituto Tumori “Giovanni Paolo II”, Bari); Cesare Gridelli, Filomena Del Gaizo (Ospedale S.Giuseppe Moscati, Avellino); Alfonso M. D'Arco, Antonietta Fabbrocini (Ospedale Civile Umberto I, Nocera Inferiore [SA]); Isabel Henriques, Ondina Campos (CHC Maternidade Bissaya-Barreto, Coimbra - Portugal); Bruno Massidda, Valeria Pusceddu (Policlinico Universitario Monserrato, Cagliari); Rossella Lauria, Valeria Forestieri (Università Federico II, Napoli); Marina Marzola, Pamela Pizzutilo (Arcispedale Sant'Anna, Ferrara); Rocco De Vivo, (Ospedale S. Bortolo ULSS 6, Vicenza); Giovanni Lo Re (Ospedale S.Maria degli Angeli, Pordenone); Saverio Danese (Ospedale S. Anna, Torino); Nada Riva (Istituto Romagnolo per lo Studio e la cura dei Tumori, Meldola [FC]); Vito Lorusso (Ospedale Vito Fazzi, Lecce); Gabriella Landi (Oncologia Medica Senologica - Istituto Nazionale Tumori, Napoli); Giuseppe Nettis (Ospedale Miulli, Acquaviva delle Fonti [BA]); Filomena Narducci (Ospedale SS.Trinità Sora [FR]); Rosalbino Biamonte (Ospedale Mariano Santo, Cosenza); Vincenzo Montesarchio (Ospedale Cotugno, Napoli); Giancarlo Balbi (Clinica Ostetrica e Ginecologica, Seconda Università, Napoli); Cosimo Sacco (Azienda Ospedaliero Universitaria, Udine); Vincenzo Arigliano (Ospedale di Bentivoglio [BO]); Francesco Perrone, Massimo Di Maio (present address: Università di Torino), Maria Carmela Piccirillo, Gennaro Daniele, Jane Bryce, Antonia Del Giudice (Unità Sperimentazioni Cliniche, Istituto Nazionale per lo Studio e la Cura dei Tumori “Fondazione G.Pascale”, IRCCS, Napoli).

## Data managers

Giuliana Canzanella, Cristiana De Luca, Teresa Ribecco, Marilena Martino, Federika Crudele, Manuela Florio, Fiorella Romano, Giovanni de Matteis, Balbina Apice, Rosa Nunziata (Napoli); Agnese Provenziani (Roma); Gianna Tabaro (Aviano [PN]); Paolo Russo (Palermo); Antonella Falzea (Reggio Calabria); Alessandra Piancastelli, Emanuela Montanari (Faenza [RA]); Giuliana Drudi, Barbara Venturini (Rimini, Cattolica [RN]); Patrizia Morselli, Beatrice Vivorio (Mantova); Roberta Guerzoni, Giorgia Razzini (Carpi [MO]); Valentina Barbato, Rita Ambrosio (Avellino); Sabrina Prisco (Nocera Inferiore [SA]); Elena Raisi (Ferrara); Bruna Stupar (Vicenza); Michela Ballardini (Meldola [FC]); Luciana Petrucelli (Lecce); Marica Gaiardo (Udine).

## Research nurses

Jane Bryce, Marzia Falanga, Immacolata Gargiulo (Napoli); Ilaria Orsini, Tonia Marra, Chiara Frediani, Alessandra Focaccetti (Roma); Annamaria Colussi, Sara Rosalen (Aviano [PN]); Michela Pilati (Rimini, Cattolica [RN]); Paola Costantini (Udine).

# References

1. Hecht JL, Kotsopoulos J, Gates MA et al. Validation of tissue microarray technology in ovarian cancer: results from the Nurses' Health Study. Cancer Epidemiol Biomarkers Prev 2008; 17: 3043-3050.

2. Rosen DG, Huang X, Deavers MT et al. Validation of tissue microarray technology in ovarian carcinoma. Mod Pathol 2004; 17: 790-797.

3. Carbotti G, Orengo AM, Mezzanzanica D et al. Activated leukocyte cell adhesion molecule soluble form: a potential biomarker of epithelial ovarian cancer is increased in type II tumors. Int J Cancer 2013; 132: 2597-2605.

4. Mezzanzanica D, Fabbi M, Bagnoli M et al. Subcellular localization of activated leukocyte cell adhesion molecule is a molecular predictor of survival in ovarian carcinoma patients. Clin Cancer Res 2008; 14: 1726-1733.

5. Piazza T, Cha E, Bongarzone I et al. Internalization and recycling of ALCAM/CD166 detected by a fully human single-chain recombinant antibody. J Cell Sci 2005; 118: 1515-1525.

6. Rosso O, Piazza T, Bongarzone I et al. The ALCAM shedding by the metalloprotease ADAM17/TACE is involved in motility of ovarian carcinoma cells. Mol Cancer Res 2007; 5: 1246-1253.

7. Aldovini D, Demichelis F, Doglioni C et al. M-CAM expression as marker of poor prognosis in epithelial ovarian cancer. Int J Cancer 2006; 119: 1920-1926.

8. Bagnoli M, Tomassetti A, Figini M et al. Downmodulation of caveolin-1 expression in human ovarian carcinoma is directly related to alpha-folate receptor overexpression. Oncogene 2000; 19: 4754-4763.

9. Lin X, Shang X, Manorek G, Howell SB. Regulation of the Epithelial-Mesenchymal Transition by Claudin-3 and Claudin-4. PLoS One 2013; 8: e67496.

10. Heinzelmann-Schwarz VA, Gardiner-Garden M, Henshall SM et al. Overexpression of the cell adhesion molecules DDR1, Claudin 3, and Ep-CAM in metaplastic ovarian epithelium and ovarian cancer. Clin Cancer Res 2004; 10: 4427-4436.

11. Choi YL, Kim J, Kwon MJ et al. Expression profile of tight junction protein claudin 3 and claudin 4 in ovarian serous adenocarcinoma with prognostic correlation. Histol Histopathol 2007; 22: 1185-1195.

12. Liu L, Gou M, Yi T et al. Antitumor effects of heparin-polyethyleneimine nanogels delivering claudin-3-targeted short hairpin RNA combined with low-dose cisplatin on ovarian cancer. Oncol Rep 2014; 31: 1623-1628.

13. Cancer Genome Atlas Research N. Integrated genomic analyses of ovarian carcinoma. Nature 2011; 474: 609-615.

14. Ferrandina G, Fagotti A, Salerno MG et al. p53 overexpression is associated with cytoreduction and response to chemotherapy in ovarian cancer. Br J Cancer 1999; 81: 733-740.

15. Karamurzin Y, Leitao MM, Jr., Soslow RA. Clinicopathologic analysis of low-stage sporadic ovarian carcinomas: a reappraisal. The American journal of surgical pathology 2013; 37: 356-367.

16. Oren M, Rotter V. Mutant p53 gain-of-function in cancer. Cold Spring Harbor perspectives in biology 2010; 2: a001107.

17. Mezzanzanica D, Balladore E, Turatti F et al. CD95-mediated apoptosis is impaired at receptor level by cellular FLICE-inhibitory protein (long form) in wild-type p53 human ovarian carcinoma. Clin Cancer Res 2004; 10: 5202-5214.

18. Sonego M, Schiappacassi M, Lovisa S et al. Stathmin regulates mutant p53 stability and transcriptional activity in ovarian cancer. EMBO Mol Med 2013; 5: 707-722.

19. Bagnoli M, Ambrogi F, Pilotti S et al. c-FLIPL expression defines two ovarian cancer patient subsets and is a prognostic factor of adverse outcome. Endocr Relat Cancer 2009; 16: 443-453.

20. Bagnoli M, Balladore E, Luison E et al. Sensitization of p53-mutated epithelial ovarian cancer to CD95-mediated apoptosis is synergistically induced by cisplatin pretreatment. Mol Cancer Ther 2007; 6: 762-772.

21. Bagnoli M, Canevari S, Mezzanzanica D. Cellular FLICE-inhibitory protein (c-FLIP) signalling: a key regulator of receptor-mediated apoptosis in physiologic context and in cancer. Int J Biochem Cell Biol 2010; 42: 210-213.

22. Sciacovelli M, Guzzo G, Morello V et al. The mitochondrial chaperone TRAP1 promotes neoplastic growth by inhibiting succinate dehydrogenase. Cell Metab 2013; 17: 988-999.

23. Matassa DS, Agliarulo I, Amoroso MR et al. TRAP1-dependent regulation of p70S6K is involved in the attenuation of protein synthesis and cell migration: Relevance in human colorectal tumors. Mol Oncol 2014; 8: 1482-1494.

24. Aust S, Bachmayr-Heyda A, Pateisky P et al. Role of TRAP1 and estrogen receptor alpha in patients with ovarian cancer -a study of the OVCAD consortium. Mol Cancer 2012; 11: 69.

25. Festa M, Del Valle L, Khalili K et al. BAG3 protein is overexpressed in human glioblastoma and is a potential target for therapy. Am J Pathol 2011; 178: 2504-2512.

26. Franco R, Scognamiglio G, Salerno V et al. Expression of the anti-apoptotic protein BAG3 in human melanomas. J Invest Dermatol 2012; 132: 252-254.

27. Rosati A, Bersani S, Tavano F et al. Expression of the antiapoptotic protein BAG3 is a feature of pancreatic adenocarcinoma and its overexpression is associated with poorer survival. Am J Pathol 2012; 181: 1524-1529.

28. Rosati A, Graziano V, De Laurenzi V et al. BAG3: a multifaceted protein that regulates major cell pathways. Cell Death Dis 2011; 2: e141.

29. Chiappetta G, Ammirante M, Basile A et al. The antiapoptotic protein BAG3 is expressed in thyroid carcinomas and modulates apoptosis mediated by tumor necrosis factor-related apoptosis-inducing ligand. J Clin Endocrinol Metab 2007; 92: 1159-1163.

30. Chiappetta G, Basile A, Arra C et al. BAG3 down-modulation reduces anaplastic thyroid tumor growth by enhancing proteasome-mediated degradation of BRAF protein. J Clin Endocrinol Metab 2012; 97: E115-120.

31. Marra L, Cantile M, Scognamiglio G et al. Deregulation of HOX B13 expression in urinary bladder cancer progression. Curr Med Chem 2013; 20: 833-839.

32. Miao J, Wang Z, Provencher H et al. HOXB13 promotes ovarian cancer progression. Proc Natl Acad Sci U S A 2007; 104: 17093-17098.

33. Yamashita T, Tazawa S, Yawei Z et al. Suppression of invasive characteristics by antisense introduction of overexpressed HOX genes in ovarian cancer cells. Int J Oncol 2006; 28: 931-938.

34. Mahajan A, Liu Z, Gellert L et al. HMGA2: a biomarker significantly overexpressed in high-grade ovarian serous carcinoma. Mod Pathol 2010; 23: 673-681.

35. Shell S, Park SM, Radjabi AR et al. Let-7 expression defines two differentiation stages of cancer. Proc Natl Acad Sci U S A 2007; 104: 11400-11405.

36. Califano D, Pignata S, Losito NS et al. High HMGA2 expression and high body mass index negatively affect the prognosis of patients with ovarian cancer. J Cell Physiol 2014; 229: 53-59.

37. Grossel MJ, Hinds PW. From cell cycle to differentiation: an expanding role for cdk6. Cell Cycle 2006; 5: 266-270.

38. Handschick K, Beuerlein K, Jurida L et al. Cyclin-dependent kinase 6 is a chromatin-bound cofactor for NF-kappaB-dependent gene expression. Mol Cell 2014; 53: 193-208.

39. Su D, Smith SM, Preti M et al. Stathmin and tubulin expression and survival of ovarian cancer patients receiving platinum treatment with and without paclitaxel. Cancer 2009; 115: 2453-2463.

40. Zhu X, Li Y, Xie C et al. miR-145 sensitizes ovarian cancer cells to paclitaxel by targeting Sp1 and Cdk6. Int J Cancer 2014; 135: 1286-1296.

41. Gao J, Tian J, Lv Y et al. Leptin induces functional activation of cyclooxygenase-2 through JAK2/STAT3, MAPK/ERK, and PI3K/AKT pathways in human endometrial cancer cells. Cancer Sci 2009; 100: 389-395.

42. Shackelford DB, Shaw RJ. The LKB1-AMPK pathway: metabolism and growth control in tumour suppression. Nat Rev Cancer 2009; 9: 563-575.

43. Hardie DG, Ross FA, Hawley SA. AMPK: a nutrient and energy sensor that maintains energy homeostasis. Nat Rev Mol Cell Biol 2012; 13: 251-262.

44. Sanli T, Steinberg GR, Singh G, Tsakiridis T. AMP-activated protein kinase (AMPK) beyond metabolism: a novel genomic stress sensor participating in the DNA damage response pathway. Cancer Biol Ther 2014; 15: 156-169.

45. Domenech E, Maestre C, Esteban-Martinez L et al. AMPK and PFKFB3 mediate glycolysis and survival in response to mitophagy during mitotic arrest. Nature cell biology 2015; 17: 1304-1316.

46. Ji C, Yang B, Yang YL et al. Exogenous cell-permeable C6 ceramide sensitizes multiple cancer cell lines to Doxorubicin-induced apoptosis by promoting AMPK activation and mTORC1 inhibition. Oncogene 2010; 29: 6557-6568.

47. Zulato E, Bergamo F, De Paoli A et al. Prognostic significance of AMPK activation in advanced stage colorectal cancer treated with chemotherapy plus bevacizumab. Br J Cancer 2014; 111: 25-32.

48. Buckendahl AC, Budczies J, Fiehn O et al. Prognostic impact of AMP-activated protein kinase expression in ovarian carcinoma: correlation of protein expression and GC/TOF-MS-based metabolomics. Oncol Rep 2011; 25: 1005-1012.

49. Jeon SM, Chandel NS, Hay N. AMPK regulates NADPH homeostasis to promote tumour cell survival during energy stress. Nature 2012; 485: 661-665.

50. Wu J, Ji F, Di W et al. Activation of acetyl-coenzyme A carboxylase is involved in Taxol-induced ovarian cancer cell death. Oncol Lett 2011; 2: 543-547.

51. Belletti B, Baldassarre G. Stathmin: a protein with many tasks. New biomarker and potential target in cancer. Expert Opin Ther Targets 2011; 15: 1249-1266.

52. Balachandran R, Welsh MJ, Day BW. Altered levels and regulation of stathmin in paclitaxel-resistant ovarian cancer cells. Oncogene 2003; 22: 8924-8930.

53. Karst AM, Levanon K, Duraisamy S et al. Stathmin 1, a marker of PI3K pathway activation and regulator of microtubule dynamics, is expressed in early pelvic serous carcinomas. Gynecol Oncol 2011; 123: 5-12.

54. Iyama T, Wilson DM, 3rd. DNA repair mechanisms in dividing and non-dividing cells. DNA Repair (Amst) 2013; 12: 620-636.

55. Lechpammer M, Resnick MB, Sabo E et al. The diagnostic and prognostic utility of claudin expression in renal cell neoplasms. Mod Pathol 2008; 21: 1320-1329.

56. Chiappetta G, Basile A, Barbieri A et al. The anti-apoptotic BAG3 protein is expressed in lung carcinomas and regulates small cell lung carcinoma (SCLC) tumor growth. Oncotarget 2014; 5: 6846-6853.

57. Kim YR, Oh KJ, Park RY et al. HOXB13 promotes androgen independent growth of LNCaP prostate cancer cells by the activation of E2F signaling. Mol Cancer 2010; 9: 124.

58. Canzonieri V, Barzan L, Franchin G et al. Alteration of G1/S transition regulators influences recurrences in head and neck squamous carcinomas. J Cell Physiol 2012; 227: 233-238.

59. Garofalo C, Koda M, Cascio S et al. Increased expression of leptin and the leptin receptor as a marker of breast cancer progression: possible role of obesity-related stimuli. Clin Cancer Res 2006; 12: 1447-1453.
